# Malaria vaccine protection against intradermal or venous parasites: a randomized phase 2b human challenge trial

**DOI:** 10.1038/s41591-025-04107-6

**Published:** 2026-01-06

**Authors:** Melissa C. Kapulu, Francesca Orenge, Domtila Kimani, Elizabeth Kibwana, Hillary Kibet, Mary Mutahi, Mehreen S. Datoo, Duncan Bellamy, Janet Musembi, Omar Ngoto, Hamisi Rashid, Stellamaris Akinyi, Mwaganyuma H. Mwatasa, Lydia Nyamako, Kelvias Keter, Rose Gatheru, Agnes Mutiso, Jennifer Musyoki, Jedidah Mwacharo, Yonas Abebe, Eric R. James, Peter F. Billingsley, Caroline Ngetsa, Moses Mosobo, Johnstone Makale, Brian Tawa, Kevin Wamae, Lynette I. Ochola-Oyier, Alison Lawrie, Fernando Ramos-Lopez, Rachel Roberts, Thomas L. Richie, B. Kim Lee Sim, Stephen L. Hoffman, Katie J. Ewer, Adrian V. S. Hill, Mainga Hamaluba, Philip Bejon

**Affiliations:** 1 Centre for Geographic Medicine Research (Coast), Kenya Medical Research Institute-Welcome Trust Research Programme, Kilifi, Kenya; 2 Centre for Tropical Medicine and Global Health, Nuffield Department of Medicine, University Oxford, Oxford, UK; 3 Jenner Institute, University of Oxford, Oxford, UK; 4 Sanaria Inc., Rockville, MD USA; 5 Department of Modernising Medical Microbiology, Nuffield Department of Medicine, University Oxford, Oxford, UK

**Keywords:** Infection, Parasite host response, Malaria, Vaccines

## Abstract

Two licensed vaccines block *Plasmodium falciparum* malaria sporozoites through anticircumsporozoite protein antibodies. In animal models, intradermal (ID) sporozoites are more readily blocked than intravenous sporozoites. We hypothesized that this complicates human studies, where infectious mosquito bites deliver a mixture of ID and venous sporozoites. Here, to test whether vaccine efficacy varies by route of inoculation, we undertook a phase 2b open, randomized controlled trial, recruiting healthy volunteers in Kenya for randomization to the circumsporozoite protein-based R21/Matrix-M vaccine (*n* = 38), thrombospondin-related adhesive protein fused to a multi-epitope string (ME-TRAP)-based vaccines (*n* = 24) or to control (*n* = 18). We enrolled 37 of these volunteers to controlled human malaria infection (CHMI) using ID or direct venous injection (DVI) of sporozoites, with PCR monitoring of parasitemia. Systemic and local postvaccination adverse events and systemic CHMI-related events were detected in 4.8%, 12.9% and 72.9% of volunteers, respectively, most commonly fever, headache and fatigue. No serious or severe adverse events were seen. Seven of 8 (88%) control volunteers and 11 of 12 (92%) ME-TRAP vaccinated volunteers, but none of the 12 R21 vaccinated volunteers receiving ID challenge met the prespecified treatment criteria for the primary endpoint. However, five of five R21 vaccinated volunteers receiving DVI sporozoites met the primary endpoint (*P* < 0.0005 by log rank across all groups). Secondary efficacy outcomes were similar; that is, R21 vaccinated volunteers receiving ID challenge did not meet any secondary endpoints, whereas volunteers for other groups met secondary endpoints between days 7 and 21 (*P* < 0.0005, *P* = 0.0001, *P* = 0.0007 and *P* < 0.0005 by log rank for time to parasitemia >20, >500, >1,000 and >10,000, respectively). R21/Matrix-M was highly protective against CHMI using ID. inoculation of sporozoites, but not against DVI sporozoites. CHMI is used in clinical development to select efficacious vaccines and to define correlates of efficacy. Correlates of efficacy for antibodies to sporozoites should also be assessed by separate DVI and ID challenges. The study was registered with ClinicalTrials.gov (NCT03947190) and PACTR (PACTR202108505632810).

## Abstract (web summary)

The R21/Matrix-M vaccine, but not the ME-TRAP vaccine, was protective against intradermal challenge with *Plasmodium falciparum* sporozoites, while neither was protective against direct venous inoculation, potentially explaining previously observed differences in protection.

## Main

*Plasmodium falciparum* (Pf) malaria remains a pressing public health problem. The Pf life cycle includes (1) sporozoites, injected by infected mosquitoes during feeding and then invade hepatocytes and (2) merozoites, which develop in hepatocytes and then initiate blood stage infection through repeated red blood cell invasion. Two malaria vaccines have recently been licensed (that is, RTS,S/AS01 (ref. ^1^) and R21/Matrix-M^2^), both of which induce immunity to the major antigen on the sporozoite surface, that is, the circumsporozoite protein (CSP). The passive transfer of anti-CSP antibodies is also partially protective^3^.

RTS,S/AS01 and R21/Matrix-M are both recommended by the World Health Organization (WHO) for use in young children and are predicted to have major health benefits on morbidity and mortality when given as a three-dose primary course between 5 and 9 months of age with a booster at 2 years^4^. Over the first year of follow-up, efficacy is reported at 78% for R21/Matrix-M^2^ and 56% for RTS,S/AS01 (ref. ^5^). Efficacy appears to wane over time as antibodies fall^6,7^ but can be restored with booster doses for both vaccines^1,8^. Reductions in mortality were seen in a 4- year implementation trial of RTS,S/AS01 (ref. ^9^).

It is unclear why antibody-based protection remains partial despite high levels of anti-CSP antibodies induced by vaccines or high concentrations of circulating monoclonal antibodies. Empirically, protection appears to be ‘leaky’, with given antibody levels being inconsistently protective^10^.

Controlled human malaria infection (CHMI) involves exposing healthy volunteers to challenge with malaria parasites, using either laboratory-reared infectious mosquitoes or cryopreserved parasites^11^. The challenge controls the dose, timing and genotype of the exposure, in contrast to the heterogeneity of exposures seen in field studies^12^. CHMI studies were undertaken in the initial clinical development of RTS,S/AS01E and R21/Matrix-M using infectious mosquito bites, where efficacy was estimated at 50%^13^ and 64–82%^14^, respectively.

Infectious mosquito bites mostly deliver Pf sporozoites into dermal layers and sporadically into capillaries^15^. Direct venous injection (DVI) of sporozoites infects human hosts with sevenfold fewer sporozoites than intradermal (ID) injection^16,17^, with many sporozoites failing to reach capillaries^18^. Furthermore, in animal models, dermal sporozoites are more readily blocked by anti-CSP antibodies compared to intravenous sporozoites^19,20^. We therefore hypothesized that vaccine protection might vary according the route of inoculation (that is, ID versus DVI).

To our knowledge, there are no CHMI studies that compare anti-CSP antibodies against different routes of inoculation of sporozoites in human volunteers. We undertook a CHMI efficacy study in volunteers vaccinated with R21/Matrix-M in Kilifi on the Kenyan Coast, comparing DVI versus ID challenge.

An alternative vaccination strategy to the induction of anti-CSP antibodies is the induction of T cells using viral vectors^21^. Previous studies of T cell-inducing vaccinations with a recombinant chimpanzee adenovirus (ChAd63) then modified vaccina Ankara (MVA), both encoding the Pf antigens full-length pre-erythrocytic antigen thrombospondin-related adhesive protein (TRAP) fused to a multi-epitope (ME) string (ME-TRAP), have shown efficacy against CHMI among UK adults^22^, no efficacy in West African children^23^, but efficacy in Kenyan adults^24^. We therefore included an additional group to retest the efficacy of ChAd63/MVA ME-TRAP in Kenyan adults to test whether this was a chance finding. T cell immunity is directed at liver stages, and there was therefore no biological reason to expect the route of sporozoite inoculation to influence efficacy, hence we restricted ME-TRAP vaccinated volunteers to ID challenge.

CHMI studies in for clinical vaccine development are typically done using infectious mosquito bites. Arguably, this reflects the challenge experienced in field studies more closely than the artificial injection of cryopreserved sporozoites and predicts outcomes in subsequent field studies^25^. However, where sensitivity to the anti-CSP antibodies induced by vaccination may vary for ID and for intravenous sporozoites, then the mixture of inoculation routes in infectious mosquito bites introduces noise and precludes a definitive measurement of correlates of efficacy. To test whether vaccine efficacy varies by route of sporozoite inoculation for R21/Matrix-M, we randomized R21 vaccinated volunteers to CHMI using ID or DVI of sporozoites.

## Results

### Participant demographics

**Fig. 1:**
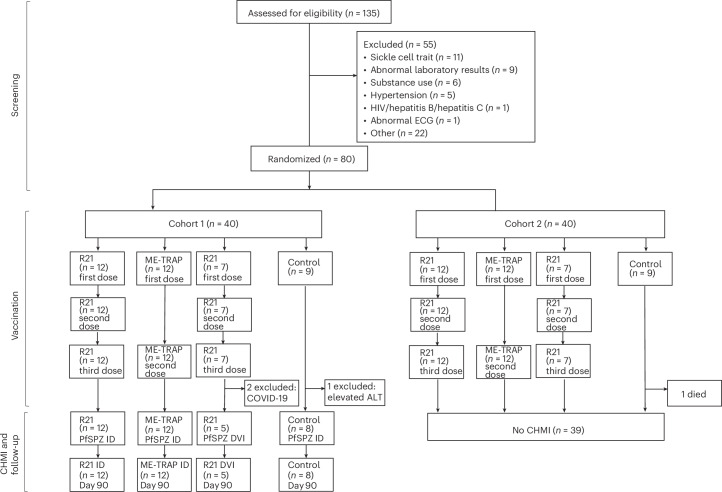
CONSORT diagram showing volunteer flow. Out of 135 volunteers screened, 80 volunteers who were eligible and met the enrollment criteria were randomized into one of four groups across two enrollment cohorts 4 weeks apart. Of the 55 excluded volunteers, those with abnormal laboratory results included low hemoglobin levels (<10 g dl^−1^ for females (*n* = 6)), thrombocytopenia (*n* = 2) and elevated levels of ALTs (*n* = 1). Other exclusions included being eligible but did not turn up for screening results (*n* = 4), not being on any method of contraception (*n* = 3), number required attained (*n* = 3), did not meet location of residence criteria (*n* = 2) and did not attend enrollment visit (*n* = 2). Other reasons, all with a frequency of one (*n* = 8) were written informed consent not signed, unstable blood pressure values, positive pregnancy test, abdominal hernia, neurofibromatosis, allergy to ibuprofen, failed test of understanding and history of schizophrenia. Seven volunteers had more than one screening failure and these were elevated laboratory values (*n* = 4), abdominal mass (*n* = 1), abnormal electrocardiogram (ECG) (*n* = 1) and substance use (*n* = 1). First dose, second dose and third dose indicate the respective vaccine doses.

One hundred thirty-five volunteers were screened for eligibility and 80 were randomized and vaccinated between 20 July 2022 and 27 December 2022. All completed scheduled vaccinations and the first cohort (that is, *n* = 40) were eligible for CHMI undertaken from 27 November 2022 to 17 December 2022. Three did not proceed to CHMI owing to SARS-CoV-2 positive results (*n* = 2) and increase in liver enzymes (alanine aminotransferase (ALT), *n* = 1), so that 37 completed CHMI (Fig. 1). One volunteer was qPCR positive for malaria parasites before vaccination, and all were qPCR negative for malaria parasites before CHMI. There was a predominance of young male volunteers (28/40, 70%) with a mean age of 28.3 years (Table 1). Volunteers were from the Mijikenda ethnic group. The male predominance among volunteers appears to relate to established gender roles^26^.

**Table 1:** Baseline characteristics based on vaccine enrollment and CHMI group

	Challenged				Not challenged				Historical control DVI (*n* = 34)
	R21 ID (*n* = 12)	R21 DVI (*n* = 7)	ME-TRAP ID (*n* = 12)	Control ID (*n* = 9)	R21 ID (*n* = 12)	R21 DVI (*n* = 7)	ME-TRAP ID (*n* = 12)	Control ID (*n* = 9)	
Age in years, mean (s.d.)	28.2 (6.1)	26.1 (4.1)	28.8 (5.1)	30.8 (8.1)	27.4 (6.8)	25.4 (6.0)	28.1 (7.1)	26.7 (3.5)	28.0 (8.0)
Male, % (*n*/*N*)	83.3% (10/12)	71.4% (5/7)	66.7% (8/12)	55.6% (5/9)	83.3% (10/12)	71.4% (5/7)	58.3% (7/12)	77.8% (7/9)	79% (27/34)
Female, % (*n*/*N*)	16.7% (2/12)	28.6% (2/7)	33.3% (4/12)	44.4% (4/9)	16.7% (2/12)	28.6% (2/7)	41.7% (5/12)	22.2% (2/9)	21% (7/34)
BMI, kg m^−2^, mean (s.d.)	21.69 (1.7)	19.72 (1.8)	23.88 (4.8)	20.55 (1.9)	21.74 (2.4)	20.71 (1.8)	23.29 (3.9)	19.79 (1.5)	31.0 (3.5)

Data are presented either as mean (s.d.), percentage and (*n*/*N*) or median and (min–max). BMI, body mass index; *n*, number of healthy volunteers enrolled to each group.

The prespecified primary endpoint was time to meeting treatment criteria (that is, reaching the parasitaemia threshold of 500 parasites μl^−1^ or any parasitaemia plus important clinical symptoms). Prespecified secondary endpoints were (1) immunology (specifically antibody responses to CSP measured by enzyme-linked immunosorbent assay (ELISA) and T cell responses to ME-TRAP peptides measured by ELISPOT) and (2) efficacy (time to parasitemia >20, >500, >1,000 and >10,000, respectively). All these prespecified outcomes are reported here and are not being published elsewhere.

### Primary outcomes

**Fig. 2:**
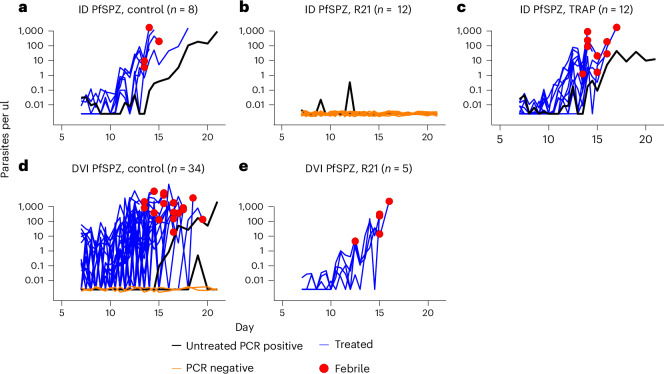
Falciparum qPCR outcome following CHMI. **a**–**e**, The qPCR results (*y* axis, log transformed) by time after inoculation (*x* axis) showing ID challenge with PfSPZ, control group (*n* = 8) (**a**); ID challenge with PfSPZ, vaccinated with R21/Matrix-M (*n* = 12) (**b**); ID challenge with PfSPZ, vaccinated with viral vectors encoding ME-TRAP (*n* = 12) (**c**); DVI challenge with PfSPZ, control group (*n* = 34) (**d**); and DVI challenge with PfSPZ, vaccinated with R21/Matrix-M (*n* = 5). Parasitemia was determined by asexual 18S ribosomal RNA gene qPCR done in Kilifi. The blue lines represent individuals who reached the required malaria diagnosis criteria, green lines represent individuals who did not meet the criteria for diagnosis but were qPCR positive, orange lines represent individuals who were qPCR negative throughout monitoring and red dots denote individuals who were febrile based on the criteria for diagnosis.

CHMI was conducted 28 days after the last vaccination. All 8 unvaccinated control volunteers and all 12 volunteers vaccinated with ME-TRAP became PCR positive for malaria parasites following ID challenge with cryopreserved sporozoites produced by Sanaria (PfSPZ), with observed typical parasite growth curves on qPCR. Seven of eight control volunteers (88%) and 11 of 12 ME-TRAP volunteers (92%) met the criteria for the primary endpoint and received malaria treatment during CHMI, and one control and one ME-TRAP volunteer remained just below the parasitaemia threshold and so did not meet the primary endpoint (Fig. 2a,c and Table 2).

**Table 2:** Malaria diagnosis outcome by vaccination and challenge group

qPCR outcome	Control ID *n* = 8	R21 ID *n* = 12	ME-TRAP ID *n* = 12	Historical control DVI (*n* = 34)	R21 DVI *n* = 5
PCR negative, % (*n*/*N*)	0% (0/8)	75% (9/12)	0% (0/12)	5.9% (2/34)	0% (0/5)
Untreated PCR positive, % (*n*/*N*)	12.5% (1/8)	25% (3/12)	8.3% (1/12)	2.9% (1/34)	0% (0/5)
Treated, febrile, % (*n*/*N*)	50.0% (4/8)	0% (0/12)	75% (9/12)	55.9% (19/34)	100% (5/5)
Treated, nonfebrile, % (*n*/*N*)	37.5% (3/8)	0% (0/12)	17% (2/12)	35.3% (12/34)	0% (0/12)

Data are presented as the percentage and *n*/*N*. Treated, nonfebrile refers to volunteers who met parasitemia thresholds for treatment without developing objective fever; treated, febrile refers to volunteers who met lower parasitemia thresholds and were treated owing to the presence of objective fever and/or other clinical signs.

In contrast, none of the 12 volunteers vaccinated with R21 met the primary endpoint following challenge with ID PfSPZ. Three out of the nine volunteers were briefly positive by PCR for malaria parasites but with no evidence of parasite growth. Hence they were protected and did not meet the primary endpoint (Fig. 2b and Table 1).

Historically unvaccinated control volunteers who had previously undergone CHMI with PfSPZ challenge by DVI showed similar growth rates to PfSPZ challenge ID volunteers, with 31 out of 34 (91%) volunteers meeting the primary endpoint, 2 out of 34 being PCR negative (6%) and 1 out of 34 being PCR positive but not meeting the primary endpoint (3%) (Fig. 2d). However, when the five volunteers vaccinated with R21 underwent CHMI with PfSPZ challenge by DVI sporozoites, all five showed typical growth curves of parasites and met the primary endpoint (Fig. 2e).

**Fig. 3:**
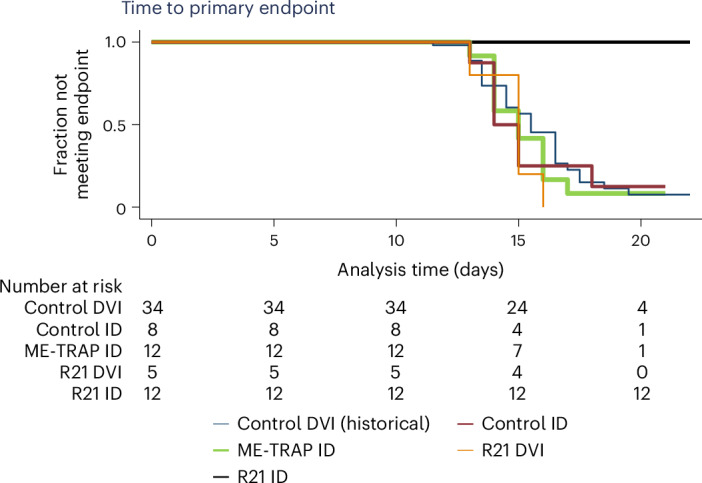
Time to primary endpoint survival analysis. Kaplan–Meier curve of the fraction of volunteers that did not meet the primary efficacy endpoint criteria for treatment (that is, 500 parasites per µl, or any parasite density plus clinically significant symptoms) following CHMI. *P* < 0.0005 by log-rank testing across all groups.

Hence, none of the volunteers vaccinated by R21 who received the ID PfSPZ challenge met a primary endpoint, whereas volunteers for all other groups met primary endpoints between days 12 and 21 (*P* < 0.0005 by log-rank survival across all groups; Fig. 3). Parasites were genotyped for AMA1, confirming the challenge parasite strain (NF54) in all cases.

#### Secondary outcomes

**Extended Data Fig. 1:**
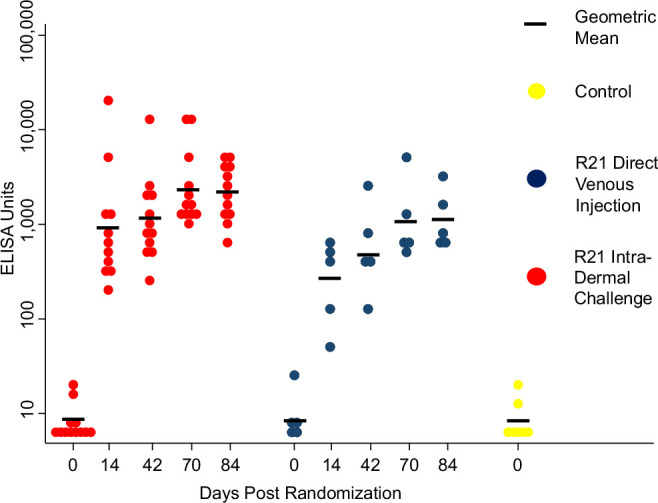
NANP vaccine-induced antibody responses over time. NANP IgG ELISA units are shown over time with individual scatter dot plots per vaccination group. Peak responses were geometric means of 2,152 and 1,113 and minimum to maximum ranges of 750-5,500 vs 610-3,300 for ID vs DVI challenged groups, respectively (p = 0.14 by Kruskal-Wallis testing). Analysis is restricted to those undergoing CHMI, that is n = 12 for R21 ID, n = 5 for R21 DVI, n = 8 for control.

Immunogenicity outcomes were the secondary outcomes. Following the vaccination course with R21, anti-NANP antibodies rose from baseline levels of just under 10 ELISA units (EU) to above 1,000 EU in the R21-vaccinated volunteers, but remained below 10 among the control group (Extended Data Fig. 1). Peak responses were geometric means of 2,152 and 1,113 and minimum to maximum ranges of 750–5,500 versus 610–3,300 for ID versus DVI challenged groups, respectively (*P* = 0.14).

**Extended Data Fig. 2:**
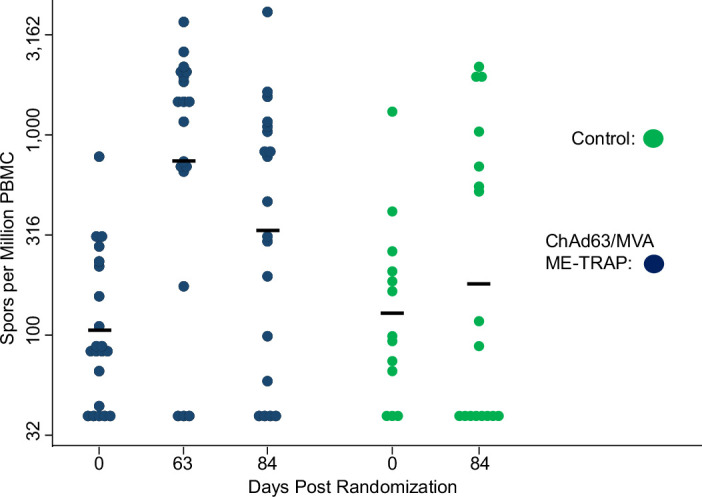
ME-TRAP vaccine-induced T cell responses over time. ME-TRAP IFNγ positive spots by ELISpot per million PBMC are shown over time with individual scatter dot plots per group and time point. Spot forming units per million PBMC (sfu) increased from geometric mean of 104 sfu (95%CI 70-153) at baseline to a peak of 735 (95%CI 364-1485, p = 0.01 for both comparisons with baseline) and 335 sfu (95%CI 162-691) on the day of challenge, compared with 127 sfu (95%CI 174 sfu (95%CI 74-409) among the control group, p = 0.064, p = 0.001, p = 0.084 for comparisons between all controls and ME-TRAP vaccinated volunteers at days 0, 63 and 84, respectively. The sample size varied according to timepoint according to the availability and viability of cells, hence for ChAd63/MVA ME-TRAP n = 21, 19 and 19 at days 0, 63 and 84, respectively, and for Control n = 13 and 17 for days 0 and 84, respectively.

Following vaccination with viral vectors encoding ME-TRAP, spot-forming units (s.f.u.) per million peripheral blood mononuclear cells (PBMCs) increased from geometric mean of 104 s.f.u. (95% CI 70–153) at baseline to a peak of 735 (95% CI 364–1,485 s.f.u., *P* = 0.01 for both comparisons with baseline) and 335 s.f.u. (95% CI 162–691 s.f.u.) on the day of challenge, compared with 127 s.f.u. (95% CI 74–409 s.f.u.) among the control group; *P* = 0.064, *P* = 0.001 and *P* = 0.084 for comparisons between all controls and ME-TRAP vaccinated volunteers at days 0, 63 and 84, respectively (Extended Data Fig. 2).

**Extended Data Fig. 3:**
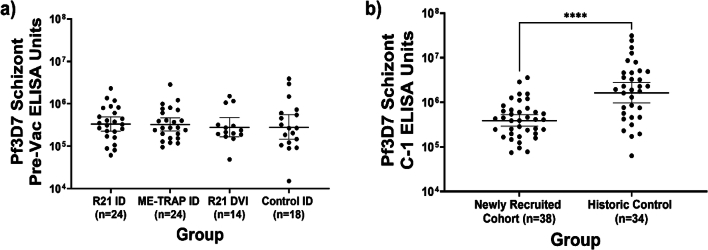
Past exposure antibody data. Schizont IgG ELISA units are shown measured at (**a**) pre-vaccination (Pre-Vac) and (**b**) day before challenge (C-1) against Pf3D7 schizont extract giving the geometric mean and error bars showing 95% confidence intervals. Historic controls had higher titres than the newly recruited cohort (geometric means of 1,244,000 EU (95% CI 693,000-2,235,000) vs 394,000 EU (95% CI 258,000-602,000), respectively, p = 0.0009), and the DVI and ID challenged groups had similar schizont extract antibodies (geometric means of 284,000 EU (95% CI 158,000-511,000) vs 434,000 EU (95% CI 252,000-745,000) respectively, p = 0.4). These tests were not pre-specified.

Volunteers had evidence of past exposure to malaria, as evidenced by prevaccination IgG antibodies against schizont extract (Extended Data Fig. 3). Historical controls had higher titers than the newly recruited cohort (geometric means of 1,244,000 EU (95% CI 693,000–2,235,000 EU) versus 394,000 EU (95% CI 258,000–602,000 EU), respectively, *P* = 0.0009), and the DVI and ID challenged groups had similar schizont extract antibodies (geometric means of 284,000 EU (95% CI 158,000–511,000 EU) versus 434,000 EU (95% CI 252,000–745,000 EU), respectively, *P* = 0.4).

**Extended Data Fig. 4:**
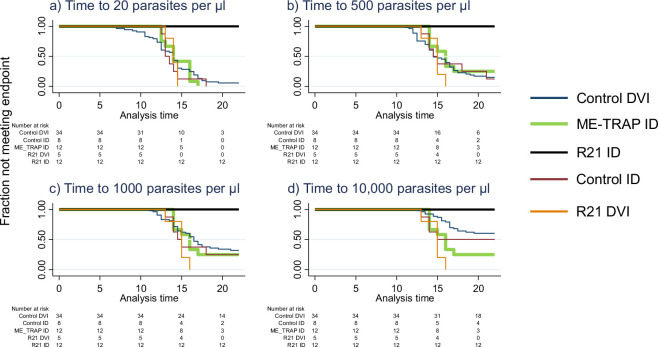
Time to: a) 20 parasites per µl; b) 500 parasites per µl; c) 1000 parasites per µl; d) 10,000 parasites per µl. None of the volunteers vaccinated by R21 and challenged by ID PfSPZ Challenge met the secondary endpoints, whereas volunteers for all other groups met secondary endpoints between days 7 and 21. Hence, none of 12 volunteers in the R21 ID group met any of the secondary endpoints, whereas 75/78 (96.1%), 60/78 (76.9%), 56/78 (71.8%) and 39/78 (50%) met secondary endpoints at p < 0.0005, p = 0.0001, p = 0.0007, p < 0.0005 by log rank survival across all groups for time to parasitemia >20, >500, >1000, >10,000, respectively.

Secondary efficacy outcomes were consistent with the primary outcome, that is, none of the volunteers vaccinated by R21 who received the ID PfSPZ challenge met the secondary endpoints, whereas volunteers for all other groups met secondary endpoints between days 7 and 21, none of the 12 volunteers in the R21 ID group met any of the secondary endpoints, whereas 75/78 (96.1%), 60/78 (76.9%), 56/78 (71.8%) and 39/78 (50%) met secondary endpoints (*P* < 0.0005, *P* = 0.0001, *P* = 0.0007 and *P* < 0.0005 by log-rank survival across all groups for time to parasitemia >20, >500, >1,000 and >10,000, respectively; Extended Data Fig. 4).

#### Safety

Following vaccination, self-limiting local solicited adverse events and general adverse events such as headache or fatigue were detected in a minority of volunteers (Table 3). No event was reported as severe and four events were reported as moderate, with a median and maximum duration of 4.5 and 5 days, respectively. General adverse events were similar in the R21 and ME-TRAP groups after the first vaccination. Specifically, headache occurred in 0/24, 1/14 (7.1%) and 5/24 (21%); fatigue in 2/24 (8.3%), 0/14 and 3/24 (12%); and malaise in 2/24 (8.3%), 0/14 and 2/24 (8.3%) of volunteers allocated to the R21 ID, R21 DVI and ME-TRAP ID groups, respectively. Fever was a more common general adverse event at the final vaccination for ME-TRAP with 5/24 (20.8%) and 8/24 (33.3%) experiencing headache, but only 1/38 volunteers vaccinated with R21 experienced fever (2.6%) and 2/38 experienced headache (5.2%). Local adverse events were rare and limited to pain at the injection site in 1/24 (4.2%) and 2/24 (8.3%) in the R21 ID and ME-TRAP ID groups, respectively.

**Table 3:** Local and general adverse events postvaccination

Local adverse events	Day 0			
	R21 ID, *n* = 24	R21 DVI, *n* = 14	ME-TRAP ID, *N* = 24	Total, *N* = 62
Pain at injection site	1 (4.2%)	0	2(8.3%)	3(4.8%)
Induration	0	0	0	0
Erythema	0	0	0	0
General adverse events				
Arthralgia	0	0	1(4.2%)	1(4.2)
Chills	0	0	1(4.2%)	1(4.2)
Fatigue	2 (8.3%)	0	3(12%)	5(8.1%)
Fever	0	1 (7.1%)	1(4.2%)	2(3.2)
Headache	0	1 (7.1%)	5(21%)	6(9.7%)
Malaise	2 (8.3%)	0	2(8.3%)	4(6.5%)
Myalgia	0	0	1(4.2%)	1(4.2%)
	Day 28			
Local adverse events	R21 ID *n* = 24	R21 DVI *n* = 14		Total *N* = 38
Pain at injection site	0	0		0
Induration	0	0		0
Erythema	0	0		0
General adverse events				
Arthralgia	0	0		0
Chills	1 (4.2%)	1 (7.1%)		2 (5.3%)
Fatigue	0	1 (7.1%)		1 (2.6%)
Fever	1 (4.2%)	1 (7.1%)		2 (5.3%)
Headache	0	1 (7.1%)		1 (2.6%)
Malaise	0	0		0
Myalgia	1 (4.2%)	2 (14%)		3 (7.9%)
	Day 56			
Local adverse events	R21 ID *n* = 24	R21 DVI *n* = 14	ME-TRAP ID *N* = 24	Total *N* = 62
Pain at injection site	0	0	4 (16.7%)	4 (6.5%)
Induration	0	0	1 (4.2%)	1 (1.6%)
Erythema	0	0	0	0
General adverse events				
Arthralgia	0	0	1 (4.2%)	1 (1.6%)
Chills	0	0	1 (4.2%)	1 (1.6%)
Fatigue	0	0	1 (4.2%)	1 (1.6%)
Fever	1 (4.2%)	0	5 (20.8%)	6 (9.7)
Headache	0	2 (14%)	8 (33.3%)	10 (16.1%)
Malaise	0	0	3 (12.5%)	3 (4.8%)
Myalgia	1 (4.2)	0	3 (12.5%)	4 (6.5%)

During CHMI, there were no immediate or early adverse events, but after day 7, fever, headache and fatigue were common in 22/37 (59.5%), 17/37 (45.9%) and 12/37 (32.0%) of volunteers, respectively (Extended Data Table 1). Six events were reported to be moderate and none were reported as severe. All events resolved on follow-up.

Abnormal blood tests, primarily transient elevations in liver enzymes and/or low white blood counts were identified in several volunteers but were not clinically significant and resolved on follow-up (Extended Data Table 2). There were clinically nonsignificant minor elevations in liver enzymes among the R21 ID group, and during CHMI there were clinically nonsignificant, transient abnormalities of liver enzymes and platelet counts (Extended Data Table 3). The safety findings were consistent with those reported previously in the same population^27^.

#### Sensitivity analysis

We saw one-off PCR positive signals on a single time point among 3 of the 12 volunteers (25%) vaccinated with R21 who received PfSPZ Challenge by ID If we include these volunteers as meeting an exploratory endpoint for purposes of a sensitivity analysis, protection is nevertheless substantial by ID (9 out of 12, 75%), and statistically significantly different from DVI (0 out of 5 protected, *P* = 0.009 by Fisher’s two-sided test).

#### Post hoc analysis

**Extended Data Fig. 5:**
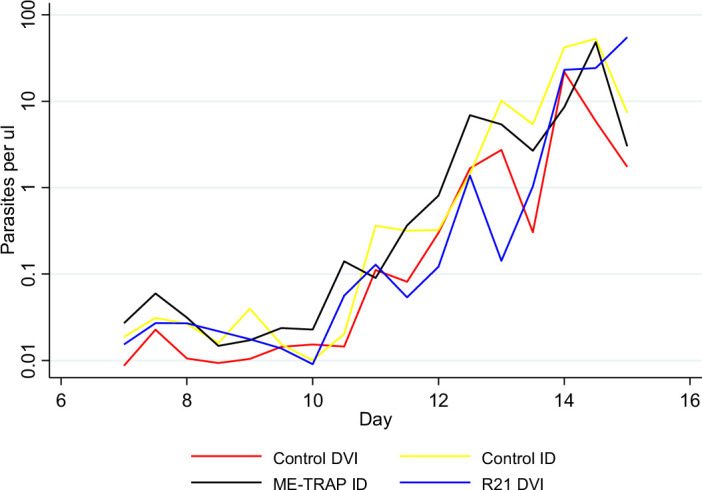
Parasite growth curves following ID and DVI inoculation. Geometric means of log PCR parasite densities following CHMI by group and day for: R21 DVI (blue line); Control ID (green line); ID ChAd63/MVA ME-TRAP (black line); and Historic Control DVI (red line). n = 8 for control DVI, n = 12 for ME-TRAP ID, n = 8 for control ID and n = 34 for historic control DVI. No data are shown for R21 ID as volunteers were not PCR positive.

The geometric mean parasite densities by PCR were similar over time following PfSPZ challenge for the control group by DVI, the control group by ID challenge, ME-TRAP vaccines by ID challenge and R21 vaccines by DVI (Extended Data Fig. 5). Hence, parasite inoculum and growth rates appear similar among these four groups.

## Discussion

Our results extend previous observations of protective efficacy by R21 against CHMI delivered by infective mosquito bites in UK adults^14^ and observations of protective efficacy by R21 against natural challenge in the field in West and East African children^2^. Instead of using infectious mosquito bites, we injected the PfSPZ challenge by needle and syringe to compare the protective efficacy of DVI versus ID inoculation of sporozoites.

R21 provided high levels of protective efficacy against PfSPZ challenge by ID, but we found no evidence of efficacy against PfSPZ challenge by DVI (that is, 0 out of 12 volunteers challenged by ID met the primary endpoint of requiring treatment versus 5 out of 5 challenged by DVI, *P* < 0.001 by Fisher’s two-sided test). Hence, despite the small sample size and the inclusion of three low-density PCR signals in a sensitivity analysis, the variation in protective efficacy of R21 by challenge route was strongly statistically significant.

Was this difference in protective efficacy by challenge route the result of differing PfSPZ doses? PfSPZ challenge is more infectious when given by DVI. To ensure comparable infectivity, we selected PfSPZ challenge doses that reliably infected unvaccinated volunteers in previous studies (that is, a sevenfold reduction for DVI at 3,200 PfSPZ challenge to 22,500 cryopreserved PfSPZ challenge ID)^16,17^. Among the unvaccinated volunteers in our study, neither infection rates (that is, 8/8 for ID challenge versus 32/34 (94%) for DVI challenge) nor growth curves (Extended Data Fig. 5) showed variation by challenge route.

We conclude that the different doses resulted in a similar effective inoculum reaching the liver in unvaccinated volunteers, and therefore that the differences seen among R21 vaccinated volunteers imply a qualitatively different interaction between anti-CSP antibodies and PfSPZ challenge that is dependent on the route of challenge. Furthermore, our data are consistent with animal models. Prior studies in mice have shown that at a given level, antisporozoite antibodies are protective against dermal inoculation but not against intravenous inoculation, and furthermore that more potent monoclonal antibodies are required to inhibit intravenous sporozoites compared to ID sporozoites^19,20^.

Taking together that (1) infectious mosquito bites deliver a variable proportion of Pf sporozoites into capillaries or dermal layers^15^, (2) that anti-CSP antibodies correlate noisily with protection against sporozoite infection^10^, with protection appearing leaky^28^, and (3) the data presented here that R21 vaccination was ineffective against PfSPZ challenge by DVI but effective against PfSPZ challenge by ID injection, we conclude that sporozoites causing infection in the face of high-titer anti-CSP antibodies induced by RTS,S or by R21 result from the occasional injection of capillary sporozoites by mosquito bites, producing noise in the correlation between antibody titers and the endpoint, which explains the leaky protection. The infrequent injection of sporozoites into capillaries (that is, one in five mosquito feeds^15^) is consistent with high levels of efficacy achieved with anti-PfCSP antibody induction despite this lack of efficacy against PfSPZ challenge by DVI: that is, if most mosquito bites deliver only dermal sporozoites, then anti-PfCSP antibodies are likely to be protective most of the time, whereas every fifth bite delivering sporozoites directly into a capillary would lead to breakthrough infection.

Correlates of protection have been defined for many other vaccines^29^ and have been used to guide policy decisions on vaccination schedules and doses for vaccines against yellow fever virus^30^, pneumococcus^31^, meningococcus A^32^ and hepatitis B^33^. Hence, defining a correlate of protection for a vaccine may translate to substantial public health benefits. While there is little doubt that anti-PfCSP antibodies are mechanistic correlates of protection, the strength of the association has varied by trial, and understanding the sources of noise in field trials will support public health decisions on future vaccine schedules.

More potent or higher-titer anti-CSP antibodies may be protective against DVI. In mouse models, high-potency monoclonal antibodies are able to inhibit sporozoites in blood whereas lower-potency monoclonals can inhibit sporozoites in skin or blood^34^, and this is further supported by findings in Saimiri monkeys challenged with vivax sporozoites^35^. Furthermore, vaccines acting through mechanisms apart from anti-CSP antibodies may not exhibit route-dependent protection^36^.

ME-TRAP was not protective against CHMI in our study and T cell induction, determined by ELISpot, was lower than that previously seen in European and Kenyan volunteers among whom protective efficacy was observed (that is, 735 s.f.u. at the peak in our study versus 2,068 and 1,451 s.f.u., respectively)^22,24^. Immunogenicity to this regimen may be suppressed by prior malaria exposure^37^ and may explain variable efficacy. In any case, the finding is not relevant to testing our hypothesis regarding antisporozoite antibodies, since the mechanism of action for ME-TRAP vaccines is cellular immunity against liver parasite stages^38^.

Our study has some limitations. We relied on historical controls for PfSPZ challenge by DVI and prior malaria exposure appears to have been higher in this group (Extended Data Fig. 4). However, the outcomes for DVI versus ID PfSPZ challenge among R21 vaccinees were based on a contemporaneous and randomized comparison, as was the comparison of R21 vaccinees versus control vaccines for PfSPZ challenge by ID, and prior malaria exposure did not differ between these groups (Extended Data Fig. 4).

Challenge by ID and DVI of cryopreserved sporozoites may not precisely duplicate infectious mosquito bites, and the latter is probably more representative of natural challenge in the field. For instance, with R21, ID and DVI PfSPZ challenge would under or overestimate protection in the field, respectively, whereas mosquito bite challenge more accurately predicted subsequent efficacy in the field^14^. On the other hand, our data suggest advantages in studying ID and/or DVI PfSPZ challenge in making comparisons between products inducing anti-CSP antibodies.

A further limitation is the sample size. Five R21 vaccinated volunteers were challenged by DVI and 12 R21 vaccinated volunteers were challenged by ID injection. In mitigation of the sample size, the outcomes completely segregated according to challenge route and were found to be significant by the primary analysis (that is, log rank for time to outcome, *P* < 0.0005).

We recruited volunteers from a specific area of Kilifi County (that is, Ngerenya), which has been at low malaria transmission since the early 2000s^39^. Previous work has shown that a high proportion of CHMI participants from this location meet the primary endpoint and that baseline antibody levels to schizont extract are relatively low^27^. We therefore recruited from this location for the present study and included data from previous participants resident in Ngerenya as a historical control population.

CHMI with infectious mosquito bites will probably remain an important step in clinical development of vaccines and is physiologically closer to challenge in the field. However, mosquito bites deliver a combination of dermal and venous sporozoites, and the concern raised by animal models is that dermal sporozoites are more readily blocked than venous sporozoites. Our study extends this finding to humans. Therefore, determining correlates of protection for anti-CSP antibodies will require either (1) large sample sizes to account for the noise introduced by mixed inoculation routes through mosquito bites or (2) a model where dermal and venous sporozoite inoculations can be studied separately. Our data suggest that the latter approach is tractable and could be used to derive antibody thresholds for protection to facilitate immunobridging studies for new products that also induce anti-CSP antibodies. Furthermore, superiority trials for new anti-CSP antibody inducing products will be underpowered in classic CHMI studies, but CHMI studies based on DVI PfSPZ can be used to specifically test for higher potency antibodies that could lead to higher levels of protection in the field against infectious mosquito bites.

## Methods

We undertook a phase IIb open-label, unblinded, randomized single-center study. Randomization and vaccination was undertaken between 20 July 2022 and 27 December 2022, and the first cohort were eligible for CHMI undertaken between 27 November 2022 and 17 December 2022 at the KEMRI Wellcome Trust Research Programme in Kilifi Kenya. One hundred thirty-five volunteers were screened for eligibility and 80 were randomized and vaccinated. The first cohort (*n* = 40) were eligible for CHMI. Three did not proceed to CHMI owing to SARS-CoV-2 positive results (*n* = 2) and an increase in liver enzymes (ALT, *n* = 1), so that 37 completed CHMI (Fig. 1). This cohort included four groups (R21/Matrix-M ID, R21/Matrix-M DVI, ChAd63/MVA ME-TRAP ID and control ID). R21 was adjuvanted with Matrix-M (R21/Matrix-M) in a 0-, 1-, 2-month schedule (Supplementary Methods). ChAd63/MVA ME-TRAP was given as two sequential doses in a 0- and 2-month schedule. For CHMI, we used *P. falciparum* NF54 sporozoites (Sanaria PfSPZ challenge (NF54)) using two alternative inoculation routes: injecting either 22,500 PfSPZ challenge ID or 3,200 PfSPZ challenge DVI.

The study was open label as (1) placebo for the different doses and routes would have been impractical and (2) outcomes were based on objective PCR data and the laboratory team were blind to allocations.

### Ethics

We complied with all relevant ethical requirements. Before commencing activity, approvals were obtained from a National IRB in Kenya and the relevant Oxford IRB (ERU (KEMRI/SERU/CGMR-C/158/3844) and OxTREC (OxTREC 32-19)) and from the medicines regulatory authority in Kenya (Pharmacy and Poisons Board (ECCT/19/11/01)). The study was registered with ClinicalTrials.gov (NCT03947190) on 07 May and PACTR (PACTR202108505632810) on 24 August 2021 to comply with in-country regulatory requirements. The use of Sanaria PfSPZ challenge (NF54) was done in accordance with an investigational new drugs application with the US Food and Drug Administration. The protocol underwent minor amendments to include methods for COVID testing and then for completion of study procedures after the first cohort underwent CHMI when a manufacturing hold was placed on PfSPZ, which prevented a second cohort CHMI.

### Study volunteers and eligibility

Following written informed consent, we recruited healthy adult men and women aged between 18 and 45 years from Kilifi North on the Kenyan Coast, where there is currently very low malaria transmission but with previous low to moderate malaria transmission. Recruitment was conducted by fieldworkers who were resident in the study areas, combined with community events to explain the study. Prospective participants were self-referring. Gender was self-reported, and given the sample size and low frequency of women volunteers, gender subgroup analyses were not conducted.

Inclusion criteria were

1. Healthy adults aged 18–45 years
2. Able and willing (in the Investigator’s opinion) to comply with all study requirements
3. Nonpregnant, nonlactating adult female or adult male
4. Agreement to refrain from blood donation during the study
5. Use of effective method of contraception for the duration of study for female participants.
6. Written informed consent
7. Plan to remain resident in the study area for 1 year following first dose of vaccination

Exclusion criteria were

1. Clinically significant congenital abnormalities as judged by the study clinicians
2. Any confirmed or suspected immunosuppressive or immunodeficient state, including HIV infection; asplenia; recurrent, severe infections; or >14 days immunosuppressant medication within the past 6 months
3. Sickle cell disease
4. Anaphylaxis in relation to vaccination
5. Clinically significant laboratory abnormality as judged by the study clinician
6. Blood transfusion within one month of enrollment
7. Hemoglobin less than 11.3 g dl^−1^ for men and less than 10 g dl^−1^ for in women, where judged to be clinically significant in the opinion of the investigator
8. Administration of immunoglobulins and/or any blood products within the last 3 months
9. Participation in another research study involving receipt of an investigational product in the 30 days preceding enrollment or planned use during the study period
10. Seropositive for hepatitis B surface antigen or hepatitis C (HCV IgG)
11. Use of systemic antibiotics with known antimalarial activity within 30 days of challenge
12. Women only; pregnancy, or an intention to become pregnant a day before challenge
13. Any significant disease, disorder or situation (including confirmed COVID-19 PCR positivity) which, in the opinion of the Investigator, may either put the participants at risk, or may influence the result of the trial
14. Confirmed parasite positive by PCR a day or 3 days before challenge

Compensation was informed by an existing guideline and approved by IRBs, and included out of pocket costs and transport, costs of contraception where applicable, an additional KES 2000 per overnight stay, applicable ATM or mobile money charges and KRA taxation.

### Enrollment and randomization

Volunteers were considered enrolled at the time of randomization, and vaccination was done on the same day. Enrollment took place within 90 days of screening. The volunteers were randomly assigned with randomization via a computer-generated sequence by an independent statistician. A randomization list in the form of password protected spreadsheet was generated using STATA and the data manager setup randomization in REDCap. Study clinicians only clicked to randomize after confirmation of eligibility criteria and immediately before vaccination for each volunteer, after which REDCap would reveal the volunteers’ randomization arm that could not be edited. The volunteers were randomized to either one of four groups.

### Vaccines and vaccination

R21 is a pre-erythrocytic protein-in-adjuvant malaria vaccine candidate. It is adjuvanted with Matrix-M based on the CSP produced by using recombinant Hepatitis B surface antigen particles expressing the central NANP repeat and the C terminus. Volunteers received three vaccinations with R21 at 4-week intervals. R21 was thawed to room temperature then mixed with Matrix-M before administration (10 μg mixed with Matrix-M 50 μg) and administered intramuscularly.

The viral vectors used were ChAd63 ME-TRAP and MVA ME-TRAP. Volunteers received a single intramuscular dose of each vaccine which was given, sequentially, 8 weeks apart. Both vaccines were thawed to room temperature before administration.

### CHMI

CHMI was undertaken 4 weeks after the final vaccination. Volunteers were tested for malaria blood stage infection by qPCR and tested for SARS-CoV-2 infection by RT–PCR on naso-pharyngeal swab. Volunteers positive for either were excluded from proceeding to CHMI.

The CHMI agent (that is, Sanaria PfSPZ challenge (NF54)) in cryovials was thawed by partial submersion of each vial for 30 s in a 37 ± 1 °C water bath. Trained study staff prepared, diluted and dispensed PfSPZ challenge to clinical staff using the diluents PBS and 25% human serum albumin within 30 min of thawing^27^. Challenge was administered in a volume of 0.5 ml using a needle and syringe by DVI at the standard dose of 3,200 PfSPZ challenge. The ID injections were administered in two separate syringes in both left and right arms with each syringe containing 11,250 PfSPZ challenge in 0.05 ml (that is, 22,500 in total). The injection sites were covered with a sterile dressing, removed no earlier than 1 h after inoculation.

The study endpoint criteria following CHMI for treatment and/or malaria diagnosis were as previously defined^27,40^, that ism reaching parasitemia threshold of 500 parasites μl^−1^ or any parasitemia plus important clinical symptoms. Volunteers who reached day 22 were treated without meeting the endpoint. Secondary efficacy outcomes were defined as time to parasitemia >20, >500, >1,000 and >10,000, respectively.

### Safety assessments

Solicited adverse events were recorded for 7 days after each vaccination, volunteers were assessed within 1 h of vaccination on the days of vaccination. Volunteers were provided with diary cards, rulers and thermometers and trained on the measurement and recording of cutaneous reactions or swelling and auxiliary temperature. Clinicians telephoned vaccinees daily for 7 days to remind them to record solicited events and to record and assess unsolicited adverse events. Further in-person examination was organized if necessary. Any unsolicited adverse events occurring between 7 and 30 days of each vaccination were recorded based on recall at 4 weeks. Serious adverse events were collected throughout the study. Causality was assessed by clinicians.

### Immunology

Plasma samples were separated from whole blood for serology. We conducted ELISAs for levels of anti-NANP antibody and anti-whole schizont antibody (3D7 strain)^41^.

PBMCs were separated by density gradient centrifugation from heparinized whole blood. Ex vivo ELISpot assays were performed over 18 h of incubation using Multiscreen IP ELISPOT plates (Millipore) and Mabtech IFNγ SA-ALP antibody kits (Mabtech). Peptide pools were tested in duplicate with 250,000 PBMCs per well. TRAP peptides were 20 amino acids in length, overlapping by 10 amino acids (ProImmune). Responses were averaged across duplicates, the background from negative control wells was subtracted and summed across TRAP pools plus ME. Plates were counted using an AID automated ELISPOT counter (AID Diagnostika GmbH), using identical settings for all plates. The lower limit of detection for the assay was 20 s.f.u.

### qPCR for parasite detection

Venous blood was collected twice daily from days 8 to 15 after PfSPZ challenge inoculation and then daily from days 16 to 22 for the detection of the 18S ribosomal RNA *P. falciparum* gene using qPCR in triplicates in a TaqMan assay using primers and probes synthesized by Eurofins Genomics: 18S Pf forward-5′GTAATTGGAATGATAGGAATTTACAAGGT 3′; 18S Pf Reverse-5’ TCAACTACGAACGTTTTAACTGCAAC 3′; 18S Pf MGB -5′ FAM- AACAATTGGAGGGCAAG-NFQ-MGB 3′ (ref. ^42^). No-template water (no-template control) was used as a negative control and cultured parasites of known quantity were used as a positive control, with sample parasite quantification undertaken against DNA extracted from known cultured parasite standards using eight serial dilutions. Standard curves were checked against the WHO external quantified quality control sample.

### Parasite genotyping

Parasite genotyping targeted the *ama1* (PF3D7_1133400) gene using amplicon deep sequencing^43^. Briefly, *ama1* amplicons spanning nucleotides 441–946 were generated from each sample, in duplicate. Deep sequencing of the sequencing library was performed on the SpotON FlowCell R10.4.1 (Oxford Nanopore Technologies, FLOW-MIN114-1) at the KEMRI–Wellcome Trust Laboratories. Sequence data analysis was performed in SeekDeep version 3.0.110, and *ama1* microhaplotypes with fewer than 250 reads or those detected at less than 5% minor allele frequency were discarded unless the microhaplotype was independently detected in additional samples at >5% frequency.

### Statistical analysis

The intent-to-treat (ITT) cohort included volunteers receiving one or more vaccines. The according-to-protocol cohort included all evaluable volunteers meeting eligibility criteria and complied with study procedures. Safety is reported ITT, immunogenicity is reported according-to-protocol and efficacy in CHMI is reported ITT for the cohort undergoing CHMI. Safety data were tabulated descriptively and immunogenicity was described by geometric means with 95% confidence intervals. CHMI data were reported by the description of quantitative PCR over time, then by frequency of outcome categories according to vaccine group. Using a comparison of proportions and assuming *P* = 0.05 and 100% infection rates in the control group, we predicted 90% power to detect 40% efficacy with *n* = 20 groups and 80% power to detect 60% efficacy with *n* = 10.

### Inclusion and ethics statement

The research was conducted and led in Kenya. Twenty-five of the 38 authors were resident in Kenya at the time of the study, and 23 authors are Kenyan citizens. The Kenyan authors played full roles in study design, implementation and data ownership. Malaria is a substantial public health problem in Kenya and a key research priority of KEMRI–Wellcome, the institution leading the research. Research roles and responsibilities were specified in the protocol, which was reviewed by IRBs in Kenya and the UK. A Kenyan author was registered for a PhD based on work from the study. CHMI studies have been previously undertaken in both the UK and Kenya and there are no severe restrictions or prohibitions. Animal welfare and environmental protection was not relevant, and biocontainment of falciparum malaria was undertaken as specified in the protocol reviewed by IRBs and with laboratory safety officer oversight. CHMI studies involve minimal risk to participants, with careful clinical supervision to reduce this risk as specified above. Relevant citations for previous CHMI and malaria studies in Kenya are included.

### Reporting summary

Further information on research design is available in the Nature Portfolio Reporting Summary linked to this article.

## Online content

Any methods, additional references, Nature Portfolio reporting summaries, source data, extended data, supplementary information, acknowledgements, peer review information; details of author contributions and competing interests; and statements of data and code availability are available at 10.1038/s41591-025-04107-6.

## Supplementary information

Supplementary Information Study protocol (VAC074 R21-CHMI Protocol Version 1.5 dated 6 April 2022 – CLEAN) and Sstatistical analysis plan.

Reporting Summary

## Figures and Tables

**Extended Data Table 1:**
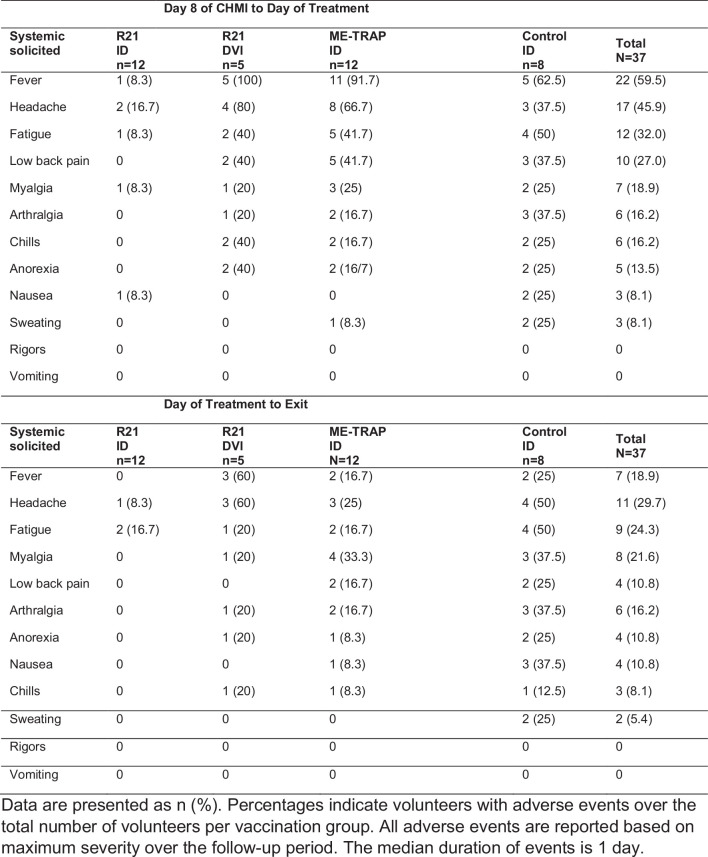
General adverse events between day 8 of CHMI to day of treatment, and day of treatment to exit of study

**Extended Data Table 2:**
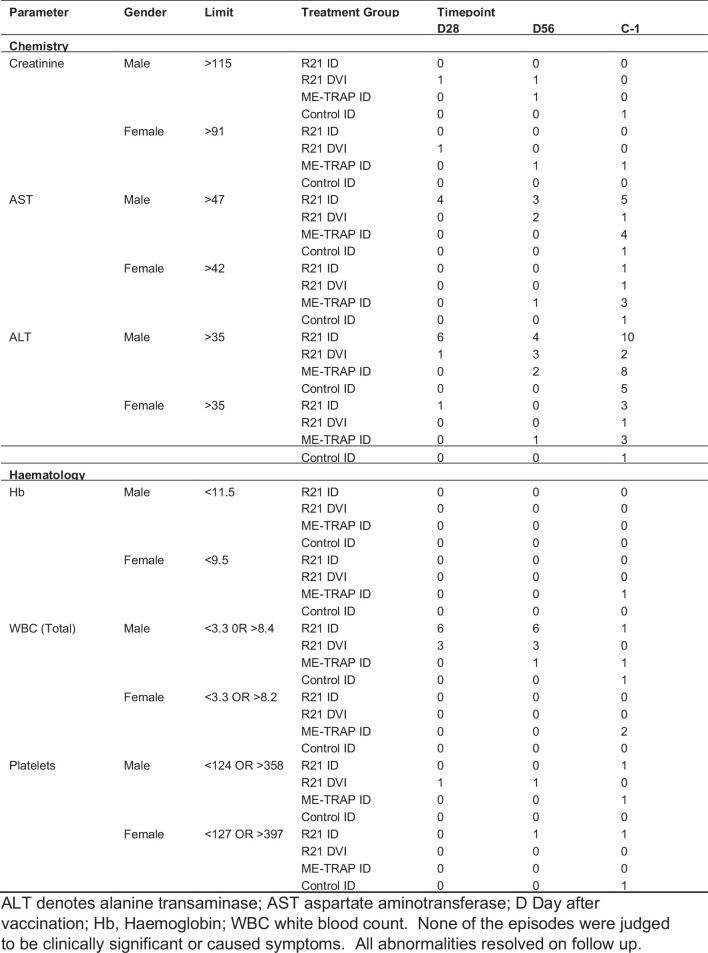
Volunteers with out-of-range safety blood results during vaccination (*n* = 62)

**Extended Data Table 3:**
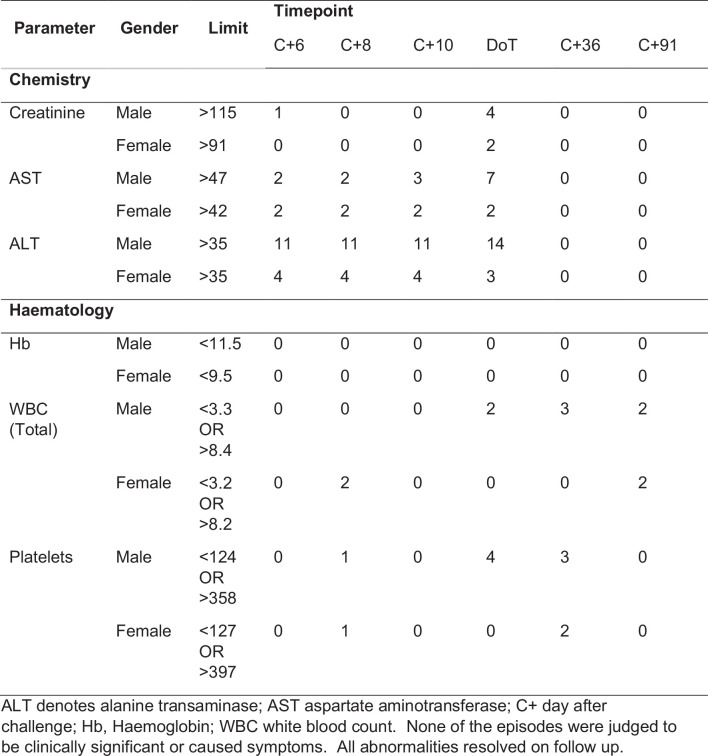
Volunteers with out-of-range safety blood results during CHMI (*n* = 37)

## Data Availability

Data are available through the online repository for KEMRI–Wellcome Trust Research Programme: Harvard Dataverse at 10.7910/DVN/TNHS14 (ref. ^44^). Individual-level data, excluding personal identifiers, will be made available in accordance with an application to the Data Governance Committee, which meets monthly (dgc@kemri-wellcome.org). There are no timing restrictions on the availability of data.
