# Supplementary material for: Malaria vaccine protection against intradermal or venous parasites: a randomized phase 2b human challenge trial
Source: Nat Med. 2026 Jan 6;32(1):178–85. doi: 10.1038/s41591-025-04107-6 (PMC12823417; doi:10.1038/s41591-025-04107-6)
Supplement: Supplementary file 1 — Study protocol (VAC074 R21-CHMI Protocol Version 1.5 dated 6 April 2022 – CLEAN) and Sstatistical analysis plan. [file 41591_2025_4107_MOESM1_ESM.pdf]

nature medicine

Supplementary information https://doi.org/10.1038/s41591-025-04107-6

# Malaria vaccine protection against intradermal or venous parasites: a randomized phase 2b human challenge trial

In the format provided by the authors and unedited

# KWTRP
# CLINICAL TRIAL PROTOCOL
# Safety, immunogenicity, and efficacy of R21/Matrix-M and ChAd63/MVA-ME-TRAP in the context of controlled human malaria infection: A Phase IIb Trial in Kenyan Adults

## GENERAL INFORMATION

| | |
|---|---|
| **Protocol Number:** | **KEMRI/SERU/CGMR-C/158/3844** |
| **Trial Registration Number:** | **NCT03947190** |
| **Investigational Product(s):** | **R21 with Matrix- M, ChAd63 ME-TRAP, MVA ME-TRAP, PfSPZ Challenge (Cryopreserved *Plasmodium falciparum* sporozoites)** |
| **Funder:** | **European and Developing Countries Clinical Trials Partnership** |
| **Tel:** | **+27 21 938 0690** |
| **Email:** | **info@edctp.org** |
| **Sponsor:** | **University of Oxford, University Offices, Wellington Square, Oxford, OX1 2JD, UK** |
| **Tel:** | **+44 1865 270000** |
| **Email:** | **research.services@admin.ox.ac.uk** |
| **Drug/Product Manufacturer:** | **1. Serum Institute of India PVT. LTD 212/2, Hadapsar, Off Soli Poonawalla Road, Pune 411028 India (R21)<br>2. Novavax AB, Kungsgatan, 109 SE- 753 18 Uppsala, Sweden (Matrix M)<br>3. Clinical Biomanufacturing Facility (CBF), University of Oxford, Old Road, Headington, Oxford, OX3 7JT, UK (ChAd63-ME-TRAP)<br>4. IDT Biologika GmbH (IDT), Am Pharmapark, 06861 Dessau-Rosslau Germany (MVA-ME-TRAP)<br>5. Sanaria Inc., 9800 Medical Center Drive A209, Rockville, MD 20850, USA (PfSPZ)** |
| **Tel:** | **1. +91-20-26993900<br>2. +46 18 16 17 00<br>3. +44 1865 744845<br>4. +49 34901 8850<br>5. +1 301-770-3222** |
| **Email:** | **1. contact@seruminstitute.com<br>2. busdev@novavax.com<br>3. cbf.enquiries@ndm.ox.ac.uk<br>4. info@idt-biologika.com<br>5. sanaria@sanaria.com** |

## Confidentiality Statement

The information contained herein is privileged or confidential and may not be disclosed unless such disclosure is required by applicable laws or regulations. In any event, persons to whom the information is disclosed must be informed that the information is privileged or confidential and may not be further disclosed by them. These restrictions on disclosure will apply equally to all future information supplied to you, which is indicated as privileged or confidential. This confidentiality statement also applies to data generated during the course of the study.

## INVESTIGATOR'S APPROVAL OF THE PROTOCOL

**Safety, immunogenicity, and efficacy of R21/Matrix- M and ChAd63/MVA-ME-TRAP in the context of controlled human malaria infection: A Phase IIb Trial in Kenyan Adults**

**Protocol Number:**

The undersigned acknowledge possession of and have read the Investigators' Brochures, [(1) R21 with Matrix M Edition 10.0 2nd December 2021; (2) ChAd63 ME-TRAP Edition 23.2 11th November 2021; (3) MVA ME-TRAP Edition 21.2 11th November 2021; and (4) PfSPZ Challenge Edition 16.0 3rd May 2021;, and protocol Safety, immunogenicity, and efficacy of R21/Matrix- M and ChAd63/MVA-ME-TRAP in the context of controlled human malaria infection: A PhaseIIb Trial in Kenyan Adults version 1.5 dated 6th April 2022. Having fully considered all theinformation available, the undersigned consider that it is ethically justifiable to give: (1) R21 with Matrix M; (2) ChAd63 ME-TRAP; (3) MVA ME-TRAP; and (4) PfSPZ Challenge to selected participants according to the agreed protocol.

I understand that all information concerning R21 with Matrix- M, ChAd63 ME-TRAP, MVA ME-TRAP, and PfSPZ supplied to me by Serum Institute of India (R21); Novavax AB (Matrix M); Clinical Biomanufacturing Facility (ChAd63-ME-TRAP); IDT Biologika GmbH (MVA-ME-TRAP); and Sanaria (PfSPZ Challenge) and/or its agents in connection with this study and not previously published is confidential information. This includes the Investigators' Brochure, Clinical Trial Protocol, Case Report Forms and any other preclinical and clinical data provided by University of Oxford.

I understand that no data are to be made public or published without prior knowledge and written approval by University of Oxford.

By my signature below, I hereby attest that I have read, understood and agreed to abide by all the conditions, instructions and restrictions contained in Protocol Safety, immunogenicity, and efficacy of R21/Matrix M and ChAd63/MVA-ME-TRAP in the context of controlled human malaria infection: A Phase IIb Trial in Kenyan Adults version 1.5 dated 6th April 2022 and in accordance with the most recent Declaration of Helsinki and Good Clinical Practice and all applicable regulatory requirements.

I acknowledge that the Sponsor of the study University of Oxford has the right to discontinue the study at any time.

**Melissa Kapulu** **Date 6th April 2022**

**Principal Investigator Signature**

---

**Mainga Hamaluba** **Date 6th April 2022**

**Co-Principal Investigator Signature**

---

# Contents

GLOSSARY OF TERMS AND ABBREVIATIONS: 7
LAY SUMMARY 9
Lay Title: A study to determine if candidate malaria vaccines are safe, effective, and induce immunity among Kenyan adults 9
What is the problem/background? 9
What questions are we trying to answer? 9
Where is the study taking place, how many people does it involve and how are they selected? 9
What does the study involve for those who are in it? 9
What are the benefits and risks/costs of the study for those involved? 11
How will the study benefit society? 11
When does the study start and finish? 11
LIST OF INVESTIGATORS 12
ABSTRACT 12
1. INTRODUCTION 13
1.1. Background Information 13
1.2. Name and description of investigational products 13
1.2.1. R21 Vaccine (protein-in-adjuvant) 13
1.2.2. ChAd63-MVA ME-TRAP 14
1.2.3. Matrix-M 16
1.2.4. Efficacy trials assessing R21c and ChAd63/MVA-ME-TRAP 17
1.3. Justification for vaccines 20
1.4. Evaluation of Efficacy in Controlled Human Malaria Infection (CHMI) 21
1.5. Justification for CHMI 22
2. TRIAL OBJECTIVES AND PURPOSE 23
2.1. Null hypothesis 23
2.2. Primary objective(s) 23
2.3. Secondary objective(s) 23
2.4. Tertiary objective(s) 23
3. TRIAL DESIGN 24
3.1. Overall Study Design and Plan Description 24
3.1.1. Recruitment 31
3.1.2. Screening 31
3.1.3. Enrolment 32
3.1.4. Study Procedures 32
3.1.4.1. Pre-Vaccination (Pre-Vac) Visit, V1 (Groups 1-3) 33
3.1.4.2. Enrolment Visit, V2 (Groups 1-3) 34
3.1.4.3. Post-Vaccination Visits 34
3.1.4.4. Post-Vaccination Visits: Boost Vaccination Visits 35
**3.1.4.5.** Post-Vaccination Visit **Error! Bookmark not defined.**
3.1.4.6. Malaria Infection Prior to PfSPZ Challenge Administration 35
3.1.4.7. Dose and Route of PfSPZ Challenge 35
3.1.4.8. Preparation and Administration of PfSPZ Challenge 36
3.1.4.9. Day Before PfSPZ Challenge (C-1) 36
3.1.5. Unscheduled Visits 37
3.1.6. End of Treatment Visit (Post diagnosis follow-up (+24hr, +48hr, +72hr) 38
3.1.7. Follow-up Visits: C+35 and C+90 38
3.1.8. Study Restrictions 38
4. SELECTION AND WITHDRAWAL OF STUDY PARTICIPANTS 38
4.1. Inclusion criteria 38
4.2. Exclusion criteria 39
4.3. Exclusion criteria on day of challenge 39
4.4. Indications for delayed vaccination 39
4.5. Withdrawal criteria 40
4.6. Managing withdrawals 40
4.7. Replacing withdrawn participants 41
5. TREATMENT OF STUDY PARTICIPANTS 41
5.1. Treatments 41

5.2. Identity of Investigational Product (IP) ... 41
5.3. Storage ... 42
5.4. Dose Selection ... 42
5.5. Timing of Doses ... 42
5.6. Packaging and Labelling ... 42
6. ASSESSMENT OF END POINTS ... 43
6.1. Specification of end point parameters ... 43
6.1.1. Primary outcome measures ... 43
6.1.2. Secondary outcome measures ... 44
6.1.3. Tertiary outcome measures ... 44
7. ASSESSMENT OF SAFETY ... 45
7.1. Adverse Events (AEs) ... 45
7.2. Definitions and monitoring of AEs ... 46
7.3. Documenting AEs ... 50
7.4. Reporting Serious Adverse Events (SAEs) and/or Unexpected AEs ... 50
7.5. Study Governance, LSM and DSMB ... 51
7.6. Emergency Procedures ... 52
7.7. Pregnancy ... 52
7.8. Protocol deviations ... 52
8. STATISTICS ... 53
8.1. Determination of sample size ... 53
8.2. Statistical and analytical plans ... 53
8.2.1.1. Statistical analysis ... 53
9. DIRECT ACCESS TO SOURCE DATA/DOCUMENTS ... 54
10. QUALITY CONTROL AND QUALITY ASSURANCE ... 54
11. INTELLECTUAL PROPERTY ... 54
12. TIME FRAME/DURATION OF THE TRIAL ... 54
13. ETHICS ... 54
13.1. Human Subjects ... 55
13.2. Community Engagement and Sensitisation ... 56
13.3. Informed Consent ... 56
13.4. Compensation ... 56
13.5. Patient Data Protection/Confidentiality ... 57
13.6. Data Sharing ... 57
14. ARCHIVING AND RECORD RETENTION ... 57
14.1. Data Management ... 57
14.2. Data capture methods ... 57
14.3. Archiving ... 58
15.1. Budget ... 58
15.2. Justification of the Budget ... 58
15.3. Insurance ... 59
16. TRIAL MANAGEMENT ... 59
17. REPORTING, DISSEMINATION AND NOTIFICATION OF RESULTS ... 59
18. REFERENCES ... 59
19. APPENDICES ... 63
19.1. Roles of Investigators ... 63
19.2. CVs of non-KEMRI investigators ... 63
19.3. Ethics Certificates of all investigators ... 63
19.4. Informed Consent documents ... 63

## GLOSSARY OF TERMS AND ABBREVIATIONS:

| | |
|---|---|
| AE | Adverse Event |
| Ab | Antibody |
| ALT | Alanine transaminase |
| B-HCG | Beta-Human Chorionic Gonadotrophin |
| BP | Blood Pressure |
| CBF | Clinical Bio-Manufacturing Facility |
| CHMI | Controlled Human Malaria Infection |
| CI | Chief Investigator |
| CLG | Community Liaison Group |
| COVID-19 | Coronavirus Disease 19 |
| CRF | Case report form |
| CS | Circumsporozoite |
| CSP | Circumsporozoite Protein |
| CTF | Clinical trials facility |
| DSMB | Data and Safety Monitoring Board |
| DTPw-HepB/Hib | Diphtheria, Tetanus, whole cell Pertussis Hepatitis B, Haemophilus influenza vaccine |
| DVI | Direct venous inoculation |
| ECG | Electrocardiogram |
| EPI | Expanded Programme of Immunisation |
| FBC | Full Blood Count |
| GCP | Good Clinical Practice |
| GIA | Growth Inhibition Assay |
| GMP | Good Manufacturing Practice |
| HBsAg | Hepatitis B surface antigen |
| HCV | Hepatitis C Virus |
| HIV | Human Immunodeficiency Virus |
| HR | Heart Rate |
| HSA | Human serum albumin |
| IC | Informed Consent |
| ID | Intradermal |
| ICH | International Conference on Harmonisation |

| | |
|---|---|
| ITNs | Insecticide treated bed nets |
| KCCRRT | Kilifi County COVID-19 Rapid Response Team |
| KCH | Kilifi County Hospital |
| LSM | Local safety monitor |
| OPA | Opsonic Phagocytosis Assay |
| OPD | Out-patient department |
| PBMCs | Peripheral Blood Mononuclear Cells |
| PBS | Phosphate buffered saline |
| PCR | Polymerase chain reaction |
| PfSPZ Challenge | Aseptic, purified, cryopreserved *P. falciparum* |
| R21c | A clinical grade R21 particle was manufactured by clinical bio-manufacturing facility (CBMF), University of Oxford. At the C-terminus of R21 a four amino acid sequence was added, EPEA, which was required for efficient immunochromatographic purification of R21; has been evaluated in early phase trials |
| R21/MM | This vaccine is manufactured by Serum Institute India, adjuvanted to Matrix M, and in the manufacturing of the vaccine no C-terminus was required for the purification process; yet to be evaluated in human subjects |
| RDT(s) | Rapid diagnostic test(s) |
| RUNMC | Radboud University Nijmegen Medical Centre |
| SAE | Serious Adverse Event |
| SCD | Sickle Cell Disease |
| SERU | Scientific & Ethics Review Unit |
| SMC | Safety Monitoring Committee |
| SOP | Standard Operating Procedure |
| SP | Sulphadoxine-pyrimethamine |
| SPZ | Sporozoites |
| SUSAR | Suspected Unexpected Serious Adverse Reaction |
| WHO | World Health Organisation |

## LAY SUMMARY

**Formal Title:** Safety, immunogenicity, and efficacy of R21/Matrix M (R21/MM) and ChAd63/MVA-ME-TRAP in the context of controlled human malaria infection: A Phase IIb Trial in Kenyan Adults.

**Lay Title:** A study to determine if candidate malaria vaccines are safe, effective, and induce immunity among Kenyan adults.

**What is the problem/background?** Malaria deaths have fallen with the advent of new drug combinations and widespread use of insecticide treated bed nets, but the hope of malaria eradication is threatened by emerging resistance to these drugs and insecticides. RTS,S, is the only malaria vaccine in advanced clinical development albeit with low efficacy. It is being evaluated for use in an infant vaccine schedule recommended by WHO, while determining the significance of any safety concerns. However, with RTS,S only a small proportion of the vaccine is made up of the malaria parasite protein. We have developed a malaria vaccine candidate, R21, which is also based on the same protein in RTS,S. The majority of RTS,S is composed of the Hepatitis B surface protein which may interfere with the immune response in infants; whereas R21 has very little of this protein on its surface. R21 also induces a good immune response and there is a good case for further clinical development of this vaccine.

**What questions are we trying to answer?** We plan to evaluate whether the malaria vaccine candidates R21 administered with Matrix M (MM) and ChAd63/MVA-ME-TRAP, are safe and effective in a Controlled Human Malaria Infection (CHMI) and induce good vaccine responses in healthy Kenyan adults (aged 18-45 years). We plan to investigate the immune responses following vaccination and CHMI.

**Where is the study taking place, how many people does it involve and how are they selected?** The study is taking place in Kilifi County at KEMRI-CGMRC and Pwani University where we will recruit and screen healthy adult volunteers from Ngerenya sub-location. A total number of 80 eligible volunteers will be required after assessment of eligibility at the screening visit to be enrolled for the vaccine efficacy study. This will be done after a programme of sensitization and information giving about the study.

**What does the study involve for those who are in it?** The following procedures are involved for those who volunteer to participate in the study. Blood samples will be taken at screening, a day prior to vaccine enrolment, prior to each vaccination, and post vaccination, a day before malaria infection, during infection and after infection treatment. Nasal and/or oral swabs will be taken for coronavirus disease 19 testing at enrolment into residence, before infection and at malaria diagnosis.

<u>Screening Procedures:</u> For individuals who are screened for potential participation, 8ml of blood will be drawn at the screening visit for serology; haematology; biochemistry and malaria testing. Medical history will also be obtained and a clinical assessment including a check of the functioning of the heart to exclude any significant heart problems. Those that pass screening will be invited to attend a pre-enrolment visit. At the enrolment visit, all eligible volunteers will be re-assessed and a blood sample of 37ml taken for haematology, biochemistry, malaria testing, and immunology.

Vaccine Enrolment and Vaccination Procedures: On the day of enrolment, a total of 80 eligible volunteers will be randomised into one of 4 groups as follows: group 1, receiving R21/MM (N=24); group 2 receiving ChAd63/MVA-ME-TRAP (N=24); group 3, receiving R21/Matrix M (N=14); and group 4 receiving no vaccine (control group, N=18). The R21/MM vaccine will be administered as a three-dose regimen at intervals of 28 days (days 0, 28 and 56) whilst the ChAd63/MVA-ME-TRAP will be administered as a prime-boost regimen at days 0 and 56. For coronavirus disease 19 transmission risk mitigation, each group will be equally split into two cohorts (i.e. group 1A and group 1B respectively) that will be enrolled and receive vaccinations sequentially over time. The sequence adopted will ensure that COVID-19 risk is mitigated. Furthermore, an additional 4 volunteers per group will be recruited to each respective group as a measure to address loss to being found positive for coronavirus disease 19 as well as loss to follow up. At each vaccination visit (except for day 0), a blood sample of 33ml will be taken from each of the volunteers. All volunteers in the vaccination groups (groups1 to 3), two weeks after each vaccine administration, a blood sample will be taken from each volunteer (30ml or 34ml after the final vaccination). Repeat blood samples may be taken to verify abnormal results. If volunteers are found to have malaria 2 weeks after the final vaccination (day 70), they will be given anti-malarial monotherapy and a blood sample taken post-treatment to test for malaria (4ml). A blood sample from the volunteers in the control group will also be collected at (day 70) for the purposes of checking for malaria and treatmentinitiated if found positive with malaria, a post-treatment sample will be taken to ensure malariainfection has been cleared. At day 77, all volunteers eligible for malaria infection will be invitedfor COVID-19 PCR testing and enrolled as residents at Pwani University after a confirmed negative test result.

Malaria Infection Procedures: A clinical assessment will be repeated a day before infection including collection of a urine sample and measurement of weight and height. Two days before infection, a repeat nasal and/or oral swab will also be taken for coronavirus disease 19 testing. Volunteers will be enrolled as residents at Pwani University eight days before malaria infection and be under medical supervision for up to 25 days depending on when they are diagnosed with malaria. This will also include volunteers from the control group who did not go through the vaccination procedures. The malaria infection will be given either via the intradermal route- into the skin (groups 1, 2, and 4) or via direct venous injection (into the blood stream) (group 3) of PfSPZ Challenge, which leads to a blood-stage malaria infection after 6.5 days of incubation in the liver to a minimum number of 64 volunteers (minimum numbers required per group as follows: group 1, N=20; group 2, (N=20; group 3, N=10; and group 4, N=14). Blood samples will also be taken: a day before the malaria infection (57mls); day five after infection (32mls); twice daily every day from day seven after infection up to the time that signs and symptoms of malaria are detected and malaria treatment initiated and completed (usually between seven to twenty-four days, 238ml); and on days thirty-five and ninety after infection (35ml at each of these time points). A total of 308mls of blood per volunteer will be drawn during this period. Observed treatment for malaria will be administered on the day of diagnosis or after twenty-one days for those who do not develop an infection.

Any volunteers who are found to be positive for coronavirus disease 19 will be referred to the Kilifi County coronavirus disease 19 rapid response team (RRT) for management and given antimalarial treatment (if found positive at day of diagnosis or day 21). Should the RRT be overwhelmed the study team will provide advice as required until the RRT is available. Participants would be isolated, treated with anti-malarials and exit the study at COVID-19 diagnosis. Study clinicians will be able to follow up the treatment of malaria of these volunteers during their referral to the response team.

**What are the benefits and risks/costs of the study for those involved?** Participants may benefit from receipt of the vaccine if it demonstrates that the efficacy in a Kenyan population is favourable and the vaccine is safe. Participants will also have close oversight and treatment support from the study team although this will primarily be for risk monitoring. There will also be wider benefit in the field of malaria vaccine research in building on data available on safety, efficacy and immune responses to R21/MM and ChAd63/MVA ME-TRAP. Controlled human malaria infection challenge via the intradermal route and direct venous injection has been conducted in seventy-eight and one hundred and eighty-four individuals respectively and has been safe and well-tolerated. The first ever controlled malaria infection study in Kenya by intramuscular injection was safely conducted in Nairobi with twenty-eight individuals. We have since conducted a controlled malaria infection study in Kenya by direct venous injection in another one hundred and sixty-one individuals in Kilifi. The risks relate to the possibility of developing an allergic reaction upon administration of the malaria infection. In addition, participants may develop signs and symptoms of malaria after infection which are unpleasant and may develop some side effects to the drugs used to treat the infection. During malaria infection, volunteers are required to be resident in a guest-house for safety monitoring and reduce the risk of acquiring field malaria infections. This is a considerable cost to the volunteers on their time but is a requirement for the study to be successful. There are no direct benefits to individuals participating in this study other than information about their health. There might be a perception of benefit from receipt of a vaccine, clinical assessment and laboratory screening.

**How will the study benefit society?** Controlled human malaria infection provides a way of studying immunity to malaria in a very detailed way that is not possible using natural infections, and we believe that this step is now necessary to inform vaccine design. This study will also evaluate promising malaria vaccine candidates, which will inform the design of a phase III study, contribute further to the area of malaria research and hopefully provide a possible tool to contribute to future eradication.

**When does the study start and finish?** The study will start upon receipt of ethical and regulatory approval; data collection, analysis and write up will take place over 2 years.

## LIST OF INVESTIGATORS

| Institutions | Investigators |
|---|---|
| KEMRI | Melissa Kapulu (PI), Mainga Hamaluba (Co-PI), Philip Bejon, Janet Musembi, Omar Ngoto, Faith Osier |
| University of Oxford | Mehreen Datoo, Alison Lawrie, Rachel Roberts, Katie Ewer, Adrian Hill (Chief Investigator -CI) |

## ABSTRACT

In the era of anti-malaria drug resistance and resistance to insecticide treated bed nets, there is an urgent need for a highly efficacious vaccine. We plan to evaluate the immunogenicity and efficacy of candidate malaria vaccines incorporating the sporozoite antigen R21 (*P. falciparum* circumsporozoite protein co-expressed with hepatitis B antigen) administered as protein-in-adjvuvant with the Matrix M and viral-vectored vaccines expressing the liver antigen TRAP (*P. falciparum* multiple epitope thrombospondin adhesion protein, ME-TRAP expressed in Chimpanzee Adenovirus 63 and Modified Vaccinia Ankara administered in a heterologous prime-boost regimen). There is evidence of safety and immunogenicity utilising these two vaccines. We plan to conduct a phase IIb trial in malaria-exposed individuals to assess the immunogenicity and efficacy of the two vaccines in the context of controlled human malaria infection. *P. falciparum* sporozoite challenge, PfSPZ Challenge.

Healthy adults aged between 18-45 years will be recruited to participate in the study after a process of information giving and sensitisation about the study. Those that provide informed consent to participate in the study will be screened to ensure they are in good health based on clinical assessment and laboratory results. Each participant will have blood tests undertaken, and physical and clinical examination to ensure suitability prior to vaccination and PfSPZ Challenge. A total of 64 participants will be enrolled for challenge and divided into four groups as follows: 20 participants to receive R21/Matrix M (R21/MM) with intradermal PfSPZ Challenge; 20 participants to receive viral-vectored ME-TRAP with intradermal PfSPZ Challenge; 10 participants to receive R21/MM with direct venous inoculation PfSPZ Challenge; and 14 participants comprising of the control group with intradermal PfSPZ Challenge. Blood tests and clinical assessments will be conducted to screen out participants with health conditions that may impact participation in the study.

Blood samples will be taken at screening and a day prior to vaccine and PfSPZ Challenge enrolment. Blood will also be taken prior to each vaccination as well as after PfSPZ Challenge administration until diagnosis with malaria, endpoint. Nasal and/or oral swabs will also be taken for coronavirus disease 19 testing at enrolment into Pwani University, before challenge and during the infection phase**.** A set threshold for malaria diagnosis will be met and once this is achieved, participants will be treated with the recommended anti-malaria drug treatment. Blood samplesto assess immunogenicity will be taken prior to vaccination, and throughout the study to assessvaccine-induced immune responses. Efficacy will be assessed in relation to the control group with serial quantitative polymerase chain reaction (PCR) monitoring of parasitaemia during PfSPZ Challenge. A day before PfSPZ Challenge, participants will be enrolled residents at Pwani University where they will be monitored daily up to a period of 25 days depending on when they are diagnosed with malaria. There will be follow-up after exit from residence for up to 90 days after PfSPZ Challenge.

# 1. INTRODUCTION

## 1.1. Background Information

Falciparum malaria remains one of the leading infectious causes of morbidity and mortality worldwide. In 2015, there were an estimated 212 million new cases of malaria and 429, 000 deaths. Fewer than half of the 91 malaria-affected countries and territories are on track to achieve the 40% reduction in case incidence and mortality by the 2020 milestone. It is estimated that in 2015, financing for malaria cost US$2.9 billion (1). The advent of artemisinin-combination therapy and increased uptake of insecticide-treated nets has resulted in significant reductions in mortality (2). However, emerging resistance to artemisinins, other anti-malarial drugs (3-5) and resistance to insecticides, may hinder the progress being made towards the ultimate goal of eradication (6). Prevention is key, and the development of a vaccine would be invaluable in the fight against malaria. The leading vaccine candidate, RTS,S/AS01, has entered pilot deployment trials in Africa. However, at present, no vaccine has demonstrated durable high-level efficacy. One of the primary strategic goals outlined by WHO in the Malaria Vaccine Roadmap, is the development of malaria vaccines with protective efficacy of at least 75% against clinical malaria, suitable for administration in malaria endemic areas and appropriate at-risk groups by 2030 (7).

RTS,S/AS01 is a pre-erythrocytic vaccine and works by inducing antibodies to sporozoites. The final results of a Phase III efficacy trial of the RTS,S/AS01 vaccine, in 7 sub-Saharan African countries were published in 2015. Overall vaccine efficacy for children aged 5-17 months was 36.3% for those who were given RTS,S/AS01 at 0,1, 2 and 20 months, and 28% for those given the vaccine at 0, 1 and 2 months. For younger infants aged 6-12 weeks, it was 26% and 18% respectively (8). Highest efficacy was noted with 4 doses but this is a challenge for implementation in the current Expanded Programme on Immunisation (EPI), which has been responsible for the much improved vaccination coverage in Africa (9). Current implementation trials are ongoing in 3 countries to determine whether RTS,S is implementable. RTS,S/AS01 vaccine, induces very strong antibody responses to the conserved central repeat of CSP, of the order of 100 - 600 micrograms per ml, weak mainly IL-2 containing CD4+ T cells and no CD8+ T cells to CSP (10). The most reproducible correlate of protection in clinical studies is with antibody levels (10, 11). R21 and viral vectors based on the ME-TRAP antigen are promising further developments for the reasons described below.

## 1.2. Name and description of investigational products

### 1.2.1. R21 Vaccine (protein-in-adjuvant)

R21 has been developed at the Jenner Institute, University of Oxford which is produced by using recombinant HBsAg particles expressing the central repeat and the C-terminus of the circumsporozoite protein (CSP). The R21 particle contains no *P. falciparum* sequences that are not present in RTS,S, which has been safely used in thousands of individuals. It is a hybrid protein of most of the CS protein of *P. falciparum* fused to the hepatitis B surface antigen. It spontaneously forms a particle as observed with RTS,S. Initial Phase I/II studies have been conducted with R21c and have shown that it is a more immunogenic particle than RTS,S in humans, with at least as high antibody levels with 20% of the RTS,S dose. This may be because R21c induces predominantly malaria rather than hepatitis antibodies (See Figure 1).

R21c was originally GMP manufactured at The Clinical Biomanufacturing Facility (CBF) in Oxford in *Pichia pastoris* and is now being manufactured at the Serum Institute of India (SII)

in *Hansenula polymorphaat.* The vaccine under manufacture at SII is currently under investigation for safety and immunogenicity in Oxford (NCT03580824) and Kenya (KEMR/SERU/CGMR-C/116/3711), has a slight modification to the structure and manufacturing process. The modification involves the removal of a C-tag, therefore, R21 does not have the four-amino acid sequence, EPEA, at the C-terminus. R21 is administered with the adjuvant Matrix M (see below) (R21/MM) in a three dose vaccine regimen at intervals of 28 days.

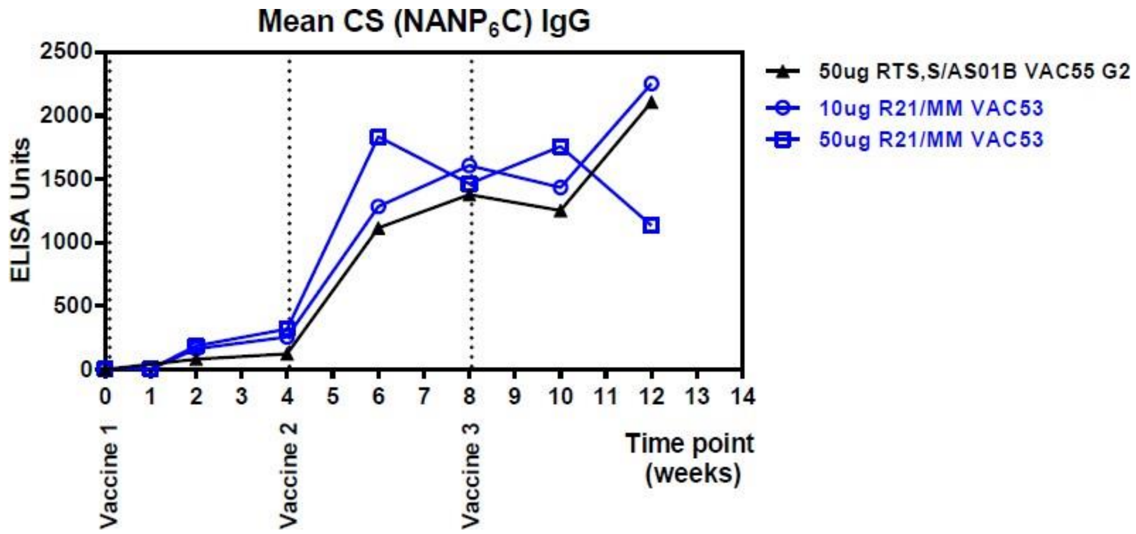


**Figure 1:** Mean IgG antibody responses to the pre-erythrocytic circumsporozoite protein.

### 1.2.2. ChAd63-MVA ME-TRAP

Viral-vectored vaccine strategies using the ME-TRAP recombinant insert have been in clinical development since 1999. ME-TRAP is a recombinant 2398 base-pair DNA insert which encodes for a single polypeptide of 789 amino acids (12, 13) containing multiple epitopes (ME) and the *P. falciparum* pre-erythrocytic thrombospondin-related adhesion protein (TRAP). ME is a string of 20 epitopes, mainly CD8 T cell epitopes, from *P. falciparum* pre-erythrocytic antigens. The individual CTL epitopes which constitute the 'multiple epitope' part of ME-TRAP are recognized by a number of common human HLA types, represent a variety (six) of potentially protective target antigens and are included to try to help ensure an immune response to the vaccine in the majority of the population vaccinated (14) The ME string is fused to the entire sequence of the T9/96 strain of *P. falciparum* TRAP. TRAP is a well characterized and abundant pre-erythrocytic stage *P. falciparum* antigen.

***Safety***

Phase I clinical testing of the safety and immunogenicity of heterologous prime boost immunisation with ChAd63 ME-TRAP followed eight weeks later by MVA ME-TRAP began in adults in the UK (15). Fifty-four healthy volunteers received ChAd63 ME-TRAP alone or followed by MVA ME-TRAP. ChAd63 ME-TRAP – MVA ME-TRAP prime-boost immunisation showed excellent safety and potent T cell immunogenicity. Vaccination was more potently immunogenic than earlier vector platforms delivering the ME-TRAP insert. Dose

ranging evaluation indicated that the preferred doses for adults, balancing reactogenicity and immunogenicity, were 5 x $10^{10}$ vp for ChAd63 and 2 x $10^{8}$ vp for MVA. These vaccines are administered via the intramuscular route.

***Immunogenicity***

The targeted mechanism of action is the induction of TRAP-specific CD8-positive T cells that eliminate *P. falciparum* – infected hepatocytes. Low to moderate antibodies to TRAP were also induced but these showed no correlation with vaccine efficacy in a sporozoite infection study, following vaccination with ChAd63 ME-TRAP and MVA ME-TRAP. In this study, vaccine efficacy was found to correlate with TRAP-specific interferon-gamma-producing CD8-positive T cells and a threshold value of 0.2% CD8+ TRAP-specific T cells was identified as a correlate of efficacy. A preclinical model of the efficacy of ChAd63-MVA ME-TRAP prime-boost immunisation showed that vaccine-induced ME-TRAP-specific effector T cells could prevent the development of blood stage infection following *P. berghei* sporozoite challenge (16). As T cell immunogenicity has progressively increased with changes in the vector platforms used for delivery of ME-TRAP, so too has vaccine efficacy (Table 3). The ChAd63-MVA heterologous prime-boost immunisation platform has induced the most potent CD8+ T cell responses to date for any vaccination approach in clinical trials for malaria and is also used to generate potent T cell responses in candidate vaccine immunization strategies for Hepatitis C and HIV.

**Table 1***:* Clinical trials of ME-TRAP encoding vaccines

| **Vaccine encoding ME-TRAP** | **T cell response mean cells/million PBMCS*** | **Efficacy** |
|---|---|---|
| DNA x 3 (13) | 48 | Nil |
| Fowl-pox x 2 | 50 | Nil |
| MVA x 3 (13) | 41 | Nil |
| DNA & MVA (13, 17) | 430 | 23% |
| Fowl-pox & MVA (18) | 475 | 25% |
| ChAd63-MVA* | 2400 | 58% |

*Summarizing maximum T cell response as measured by IFNγ producing ELISPOT at peak time point post final boost, and clinical efficacy defined as sterile protection or significant delay to parasitemia post CHMI by mosquito bite. Note that efficacy here includes both sterile efficacy and delay to patency.

***Efficacy***

Delivery of ME-TRAP as a recombinant insert in the inactivated viral vectors, ChAd63 and MVA, administered eight weeks apart ("heterologous prime-boost immunisation") has demonstrated efficacy in UK sporozoite challenge adult clinical trials. This was demonstrated in two clinical trials. In this first trial (MAL034), 14 volunteers who underwent malaria challenge after prime boost vaccination with ChAd63 ME-TRAP (5x$10^{10}$ vp IM) and MVA ME-TRAP (2x$10^{8}$ pfu ID); 10 volunteers underwent malaria challenge after vaccination with

ChAd63 ME-TRAP alone; and 14 volunteers enrolled in the control group. 57% of ChAd63-MVA-ME-TRAP vaccines demonstrated clinical efficacy: 3/14 (21%) volunteers demonstrated sterile protection, and 5/14 (36%) volunteers demonstrated partial protection (an increased time to clinical malaria). Kaplan-Meier survival analysis demonstrated significant delay in time to patent parasitaemia in the prime-boost vaccines compared to the control group as measured by blood film microscopy ($p = 0.008$, log rank test,) or a real time quantitative PCR assay (to > 20 parasites/ml, $p = 0.016$, log rank test). On re-challenge 8 months later, all 3 sterilely protected ChAd63-MVA-ME-TRAP volunteers demonstrated evidence of persisting efficacy, with 1 volunteer demonstrating sterile protection and the other two delays to patency. In the second efficacy trial (VAC45), volunteers received either ChAd63-MVA-ME-TRAP vaccination, ChAd63-MVA-CSP vaccination, or were unvaccinated controls. Of 15 ChAd63-MVA ME-TRAP volunteers, 2 volunteers showed sterile protection (failure to develop blood stage infection by Day 21) and five further volunteers showed a delay in time to patent blood stage infection (to at least Day 13, 2.5 days beyond the mean for unvaccinated controls). Of 15 ChAd63-MVA-CSP volunteers, 1 volunteer showed sterile protection and three further volunteers showed a delay in time to patent blood stage infection.
A clinical field efficacy trial conducted in Kilifi where healthy volunteers were given ChAd63 MVA ME-TRAP, showed that vaccination reduced the risk of infection with *P. falciparum* by 67% ($p=0.002$) during 8 weeks of monitoring. This study demonstrated some protective efficacy against malaria infection with a T cell-inducing vaccination strategy among adults in a malaria-endemic area in Kenya, whereas previous T cell-inducing vaccines which have been partially effective in controlled human malaria infection (CHMI) studies, have been ineffective in field studies (19).

### 1.2.3. Matrix-M

Matrix-M (MM) is a 40nm-sized complex containing the adjuvant-active saponin *Quillaja saponaria*, phospholipid and cholesterol. Quillaja saponins are triterpene glycoside substances derived from the tree *Quillaja saponaria*. The molecular weights of the different saponins range from 1800 - 2000 Da. In water, saponin in concentrations of 200-500 ppm exist as monomers; at higher concentrations they aggregated as micelles, with a molecular weight of approximately 100,000 Da. Saponins are surface-active compounds with a variety of applications including in agriculture, feed, food and beverage, mining, and veterinary vaccines, and are currently being investigated in human vaccine clinical trials. In aqueous solution, saponins are excellent adjuvants and are used in commercial veterinary vaccines, e.g., vaccines against foot-and-mouth disease, bovine mastitis, feline leukemia and equine influenza. Matrix M is being developed by Novavax as an adjuvant for their H7N9 influenza vaccine and a licensure application to the FDA for this influenza vaccine with Matrix M is planned.

***Matrix-M – effect in humans***

More than 1400 human subjects have received Matrix-M, as an adjuvant for vaccines against several diseases including malaria, influenza and ebolavirus disease. Collectively, the clinical data with Matrix-M at doses up to 75µg shows that vaccines containing the adjuvant have reversible acute reactogenicity (i.e. self-limiting fever and pain) but are generally well-tolerated and demonstrate an acceptably safety profile. Matrix-M adjuvanted vaccine formulations have

also demonstrated a clear immunogenicity benefit, with documented evidence of antigen dose-sparing (20). Further details on the pre-clinical and clinical assessment of Matrix-M adjuvanted vaccines can be found in the Safety Data Supplement provided by Novavax. Since 2018, Matrix-M has been used in 3 large Phase III trials by Novavax for influenza, RSV and now COVID-19 with many tens of thousands of subjects dosed. The safety profile remains very reassuring. Novavax now plans to supply 1.1 billion doses of Matrix-M for their COVID vaccine to COVAX working in partnership with Serum Institute of India in 2021.

### 1.2.4. Efficacy trials assessing R21c and ChAd63/MVA-ME-TRAP

We conducted a phase I/IIa sporozoite challenge study (VAC65) to assess the safety and protective efficacy of adjuvanted R21c at different doses and the combination malaria vaccine candidate regimen of adjuvanted R21 + ChAd63 and MVA encoding ME-TRAP in naïve individuals. 66 volunteers were enrolled in the trial (See Table 2 and Figure 2). Challenge A was enrolled, vaccinated and followed-up from November 2016 – February 2017. CHMI took place 30-31st January 2017. Challenge B was enrolled, vaccinated and followed-up from July 2017-October 2017. CHMI took place 17th-18th September 2017, eight and a half months after the last vaccination. The volunteers in Group 6 were recruited from a previous R21c trial.

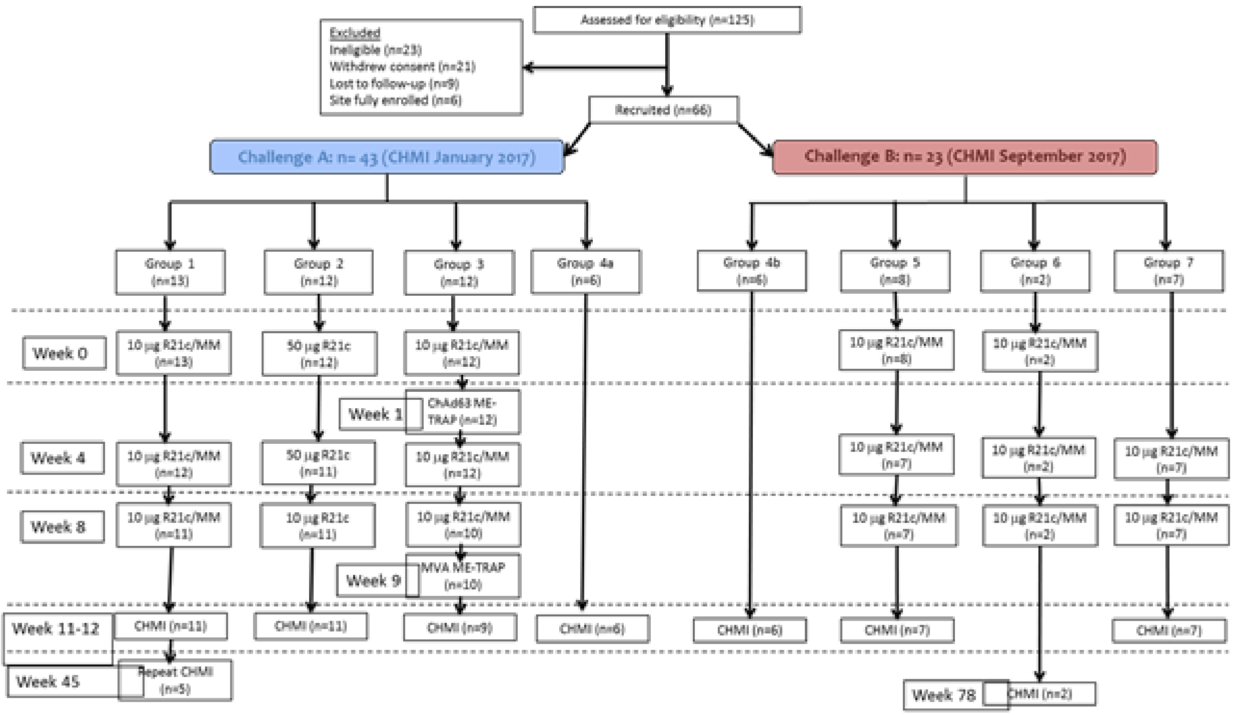


**Figure 2: VAC065 flow chart of study design and volunteer recruitment.** Abbreviations: MM, Matrix-M. Abbreviations: ChAd63, chimpanzee adenovirus serotype 63; ME-TRAP, multiple epitope string fused to the thrombospondin-related adhesion protein; MVA, modified vaccinia Ankara; ChAd63 ME-TRAP wasadministered at 5 x $10^{10}$ viral particles and MVA ME-TRAP was administered at 2 x $10^{8}$ plaque forming units. Atotal of 125 volunteers were screened and 66 were enrolled in total.

**Table 2:** VAC65 Vaccination groups.

| **Week** | **0** | **1** | **4** | **8** | **9** | **11-12** | **32-64** | **52-104** |
|---|---|---|---|---|---|---|---|---|
| **Group 1 n=12** | 10µg R21c /50µg Matrix M | | 10µg R21c /50µg Matrix M | 10µg R21c /50µg Matrix M | | CHMI | repeat CHMI of sterilely protected volunteers | |
| **Group 2 n=12** | 50µg R21c /50µg Matrix M | | 50µg R21c /50µg Matrix M | 10µg R21c /50µg Matrix M | | CHMI | repeat CHMI of sterilely protected volunteers | |
| **Group 3 n=12** | 10µg R21c /50µg Matrix M | ChAd63 ME-TRAP | 10µg R21c /50µg Matrix M | 10µg R21c /50µg Matrix M | MVA ME-TRAP | CHMI | repeat CHMI of sterilely protected volunteers | |
| **Group 4a^ n=6** | | | | | | CHMI (controls) | | |
| **Group 5 n=12** | 10µg R21c /50µg Matrix M | | 10µg R21c /50µg Matrix M | 2µg R21c / 50µg Matrix-M | | CHMI | repeat CHMI of sterilely protected volunteers | |
| **Group 6* n=1-8** | 10µg R21c /50µg Matrix M | | 10µg R21c/50µg Matrix M | 10µg R21c /50µg Matrix M | | | | CHMI |
| **Group 7 n = 8** | 10µg R21c /50µg Matrix M | | 10µg R21c /50µg Matrix M | | | Week 7-8 CHMI | | |
| **Group 4b[%] n=4-6** | | | | | | CHMI (controls) | | |

^Group 4a were infectivity controls when groups 1-3 had initial CHMI (challenge A); [%]Group 4b were infectivity controls when group 5 and 6 had initial CHMI and sterilely protected volunteers in groups 1-2 had repeat CHMI (challenge B); *Group 6 received their vaccination during their enrolment into VAC053.

### *Safety and Immunogenicity*

R21c in combination with Matrix M was safe and well tolerated with adverse events being predominantly mild in nature and self-limiting. Vaccine injection site pain was the most common local adverse event and was predominantly mild in severity. 10/10/10µg R21c with MM had a favourable reactogenicity profile compared to RTS,S/AS01B. Comparison was made with data from a previous clinical trial conducted in Oxford where volunteers received three doses of RTS,S/AS01B. The adverse event profile was statistically significantly improved with 10µg R21/MM after each dose in comparison to 50µg RTS,S/AS01B (Vaccination 1 - p<0.0001, Vaccination 2 - p<0.0001 and Vaccination 3 - p=0.005; chi-squared test comparing adverse event rates stratified by grade). There was also a considerably higher percentage of moderate and severe adverse events reported by volunteers receiving RTS,S/AS01B. There were no statistically significant differences between the IgG responses to NANP between the R21c/MM groups and there was a broad range in magnitude of antibody responses in those protected against CHMI. This suggests that the quality of the initially induced humoral response was relevant to efficacy in addition to the magnitude of the response. Additionally, antibody responses were well maintained at 8.5 months' post-vaccination.

### *Efficacy*

High level efficacy (82%) was observed with 10/10/10µg R21c with Matrix M; amongst the highest efficacy reported for any three-dose vaccine (See Figure 3). The addition of viral-vectored vaccines to this or a fractional third dose of R21c did not result in improved efficacy. Durable vaccine efficacy of 60% was observed at 8.5 months. Efficacy of 57% was observed with 10/10mg R21c/MM, which is amongst the highest level of CHMI efficacy reported for a two-dose malaria vaccine regime. (See Figure 4).

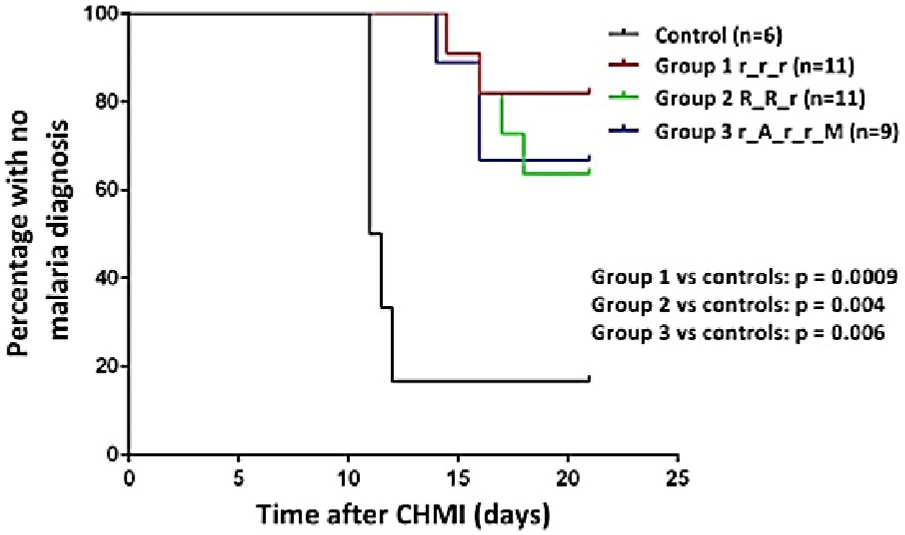


**Figure 3: Challenge A efficacy.** Efficacy of Group 1 low dose 10µg, 10 µg, 10 µg regime = 82% sterile protection. (Corrected VE = 78% for Group 1 volunteers allowing for 1/6 controls not infected). Group 2- R21c 50 µg, 50 µg, 10 µg with Matrix M 50 µg. Group 3- R21c 10 µg, 10 µg, 10 µg with Matrix M 50 µg and ChAd 63 MVA ME-TRAP

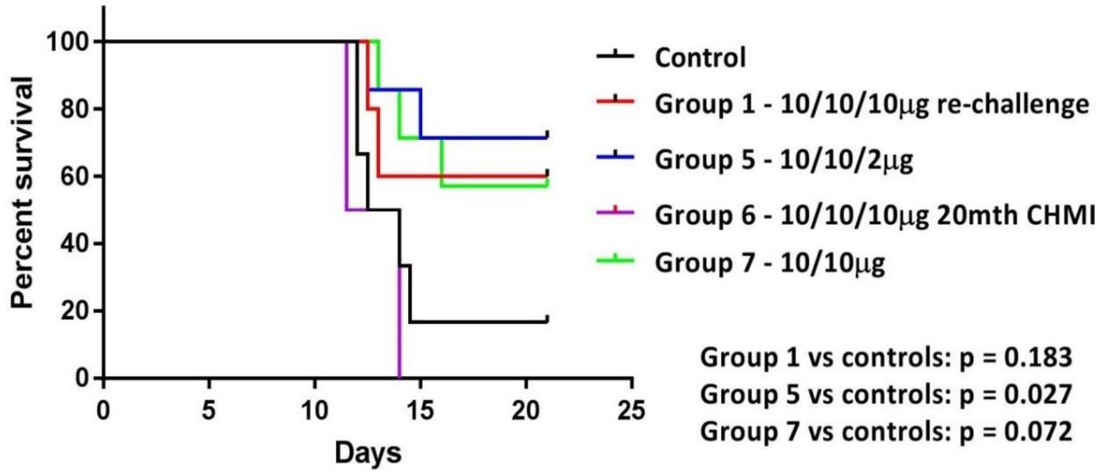


**Figure 4: Challenge B efficacy.** Efficacy of two dose 10 µg, 10 µg regime at 3-4 weeks = 57%. (Group 7). Durable efficacy of three dose 10 µg regime at 8.5 months = 60% (Group 1)

In conclusion, this Phase I/II clinical trial showed that R21c adjuvanted with Matrix- M was safe and well tolerated in UK subjects. The vaccine regime of 10/10/10µg R21 with MM showed good efficacy with durable efficacy and antibody response observed at 8.5 months. The two-dose regime also displayed high level efficacy.

Since these studies, R21 in combination with Matrix M has been shown to be safe, well tolerated and immunogenic in children and adults in Kenya (Phase Ib) and Burkina Faso (Phase IIb). Preliminary data from the Phase Ib trial in Kenya (KEMRI/SERU/CGMR-C/116/3711, ECCT/18/12/01) has shown that the vaccine is safe, well tolerated and immunogenic in in 91 adults, children and infants. There were no Suspected Unexpected Serious Adverse Reactions (SUSARs) or Serious Adverse Events (SAEs) related to vaccination following the priming doses. In addition, the Phase IIb trial in Burkina Faso (multiple doses in 450 children aged 5-36 months) has reported no SUSARs or SAEs related to vaccination. Most adverse events were mild fever in less than 50% of those vaccinated.

### 1.3. Justification for vaccines

In summary, two different candidate vaccine strategies (R21 and viral-vectors) have been described. Both strategies target the pre-erythrocytic stage of the malaria parasite life cycle but induce specific immune responses through distinct mechanisms. Each vaccine strategy has demonstrated pre-erythrocytic stage immunity and this trial proposes to assess the sterile efficacy of adjuvanted R21 and viral-vectored vaccines.
The justification for progressing with R21 is as follows:

- The efficacy obtained with RTS, S alone could be improved. R21 was designed to have a higher ratio of malaria antigen (i.e. CS) to hepatitis B surface antigen. This allows immunogenicity to be sustained with lower doses than with RTS, S, but does not translate into higher levels of immunogenicity than RTS, S when judged by antibody concentrations. However, it appears that the antibody is of improved quality, since protective efficacy in both mouse and human challenge studies is higher than that observed with RTS, S
- R21 is likely to be cheaper to produce and manufacture than RTS, S. Even if R21 turns out not to have substantially higher efficacy than RTS, S when tested in the field (despite

the indications at pre-clinical and human challenge stage that efficacy is higher), RTS, S has been given a positive scientific opinion by the EMA and is currently undergoing implementation trials. It appears to be judged potentially cost-effective by WHO to justify these implementation trials, and therefore R21 would be cost-effective.

- There are some safety concerns related to RTS, S (i.e. the possibility of aseptic meningitis and of increased mortality in girls due to non-specific vaccine effects). The significance of these concerns is unclear at present, and WHO has considered that implementation trials are needed in part to clarify these. If further data support any of these concerns it is more likely that they will be related to the adjuvant than to the antigen, and it would be a strategic advantage to public health to have a 2$^{nd}$ generation RTS, S product with a different adjuvant in development to reduce the risk of that eventuality.

The justification for progressing with viral-vectors is as follows:

- ChAd63 ME-TRAP and MVA ME-TRAP heterologous prime-boost immunisation shows durable partial efficacy in malaria challenge studies in the UK, and a CD8+ T cell correlate of efficacy has been identified (Ewer et al Nat Comm 2013; IB for MVA ME-TRAP) which may help predict the potential efficacy of the strategy based on immunogenicity results of clinical studies.
- The vaccination strategy shows potent immunogenicity in malaria-endemic populations, including infants and children, and has an excellent track record of safety.
- Clinical evaluation indicates that there may be flexibility to modify the ChAd63 ME-TRAP / MVA ME-TRAP vaccination regimen to be compatible with EPI programs if necessary for field-applicability in infants and children, and to boost immune responses over a longer period of time with repeat vaccination.

### 1.4. Evaluation of Efficacy in Controlled Human Malaria Infection (CHMI)

CHMI studies have previously depended on exposure to mosquito bites, which places logistic demands in terms of the incubation of blood-stage cultures, feeding and infection of laboratory reared mosquitoes in sufficient numbers, which then cannot be stored. These hurdles can be overcome by using cryopreserved sporozoites, which can be transported to and used in sites that lack the facilities necessary for mosquito-bite challenge (21). The company Sanaria has addressed various technical challenges in developing this technology, including the need to prepare large numbers of aseptic, purified and viable sporozoites which can be cryopreserved and injected by syringe when required. Several studies have been conducted to date to establish the safety and efficiency of this approach (21-23) and following a scale-up in production, CHMI studies are now possible at very much greater scale. The direct venous inoculation (DVI) of sporozoites is an efficient route of administration and provides a reproducible inoculum. Recent studies have shown that an infection rate by intramuscular injection of 75,000 sporozoites is comparable with 3,200 sporozoites delivered by DVI and 5 mosquito infected bites (24, 25). Thus, there are fewer sporozoites required using the DVI route of administration to achieve the same infection rates observed by the intramuscular route of administration (24). The DVI route has further been proven to be safe and reliable in resulting in infection of all naïve volunteers administered (25).

As proof of concept, a pilot study of CHMI in Nairobi, including 28 volunteers with serial quantitative PCR monitoring (26), using PfSPZ Challenge was conducted (SSC 2313). The parasite growth rate was nearly flat in one volunteer, a pattern not seen in more than several hundred CHMI studies in malaria-naïve volunteers (27). These pilot studies recruited in

Nairobi, and therefore only a few of the volunteers had substantial prior exposure to malaria. We anticipate more volunteers with markedly reduced *in vivo* parasite growth rates where exposure to malaria is higher, as inferred from natural challenge studies in the Gambia (28). We have since conducted a larger study involving DVI in semi-immune Kenyan adults (KEMRI/SERU/CGMR-C/029/3190) where CHMI has been safe with evidence of parasite growth being modified by immunity. Thus, the CHMI platform in semi-immune volunteers will provide a valuable resource for characterizing clinical immunity in a well-controlled experimental setting. More than 1,000 volunteers have participated in CHMI studies that have been conducted and myocardial events have occurred in five volunteers in the Netherlands following mosquito bite challenge, one with prior cardiovascular risk factors and evidence of atherosclerotic infarction, and the others without risk factors and a less clear clinical picture (30, 31) (unpublished findings). These five isolated events were not definitively linked to CHMI and are not widely recognized consequences of natural malaria infection. All occurred in the Netherlands and no such events have occurred in the CHMI studies conducted elsewhere, including CHMI studies in Tanzania, Kenya and Gabon. Furthermore, no participant in CHMI has thus far developed an illness meeting criteria for severe malaria. Thus overall, administration of PfSPZ Challenge in CHMI appears to be safe (24, 25).

The intradermal inoculation of PfSPZ Challenge has also been tried and was well tolerated, only associated with mild local and systemic adverse events. In a study conducted at RUNMC, 3 groups of 6 volunteers were challenged with 3 different doses of PFSPZ at 2 injection sites: 2,500, 10,000 and 25,000. In all 3 groups, 5 out of 6 inoculated volunteers were successfully infected (83%) with *P. falciparum*. The pre-patent period was similar for all dose groups; 13.0, 12.7 and 13.0 days respectively (21). In another trial conducted in Oxford, PfSPZ challenge of 2, 500 parasites administered intradermally at 2 injection sites was well tolerated and infected 5 out of 6 volunteers (29). Intradermal inoculation of PfSPZ has also been conducted in a malaria endemic area; Bagamoyo, Tanzania. Volunteers were injected intradermally with 10,000 and 25, 000 PfSPZ Challenge. 11 out of 12 and 10 out of 11 volunteers, respectively, developed parasitaemia (23). This suggests that this is possibly a good CHMI model to take forward and conduct more studies with, when testing potential malaria vaccine candidates in malaria endemic areas. Intradermal challenge may be a closer model to natural infection in consideration of subjects being infected via mosquito bites in comparison to direct venous inoculation. A comparison of vaccine protection for intradermal versus intravenous sporozoites will provide further information regarding mechanisms of protection of CS antibodies.

### 1.5. Justification for CHMI

*P. falciparum* malaria is a microbe particularly well suited to challenge studies. It has a relatively short asymptomatic period, a well-established diagnostic laboratory test (thick film microscopy or qPCR), and no long-term sequelae or infectious state following appropriate and timely treatment. Studies involving CHMI are a powerful tool for investigating malaria vaccine and prophylactic drug efficacy (32). With an increasing number of candidate malaria vaccines being developed, the number of centers conducting CHMI studies is expanding (32). Deliberate infection of humans with malaria were first performed in 1917, primarily as a therapy for patients with neurosyphilis (33). Following the development of protocols for the continuous culture of *P. falciparum* (34) and for the generation of mature *P. falciparum* gametocytes *in-vitro* (35), it became possible to produce laboratory-reared infectious mosquitoes, meaning that CHMI trials could be performed more routinely (36). The first well-documented CHMI study with laboratory-reared infectious mosquitoes was carried out in 1986 (37). The following year, the efficacies of the first recombinant protein and synthetic peptide *P. falciparum* vaccines were

reported for experimentally infected volunteers (38, 39). CHMI has now become established as a key tool to assess the efficacy of novel malaria vaccines and drugs; a total of 1,343 volunteers were experimentally infected with *P. falciparum* between 1985 and 2009 (40)(32). As CHMI trials are carried out in a controlled environment, they allow detailed evaluation of parasite growth and immunological responses, providing key information for vaccine and drug development (32). While it is not an investigational product, PfSPZ Challenge is being used as a challenge agent with which to evaluate the efficacy of vaccines.

# 2. TRIAL OBJECTIVES AND PURPOSE

## 2.1. Null hypothesis

There is no variation in risk of malaria across the groups.

## 2.2. Primary objective(s)

1. To assess the safety and tolerability of adjuvanted R21/MM and heterologous prime-boost regime of ChAd63-MVA ME-TRAP in healthy adult volunteers.

2. To assess the safety of intradermal sporozoite infection dose in semi-immune healthy adult volunteers.

3. To assess the efficacy (occurrence of *P. falciparum* parasitemia, (assessed by qPCR) of adjuvanted R21 and heterologous prime- boost regime of ChAd63-MVA ME-TRAP against malaria sporozoite challenge, in healthy adult volunteers.

## 2.3. Secondary objective(s)

1. To assess humoral immunogenicity generated in individuals of adjuvanted R21(R21/MM) and heterologous prime-boost regime of ChAd63-MVA ME-TRAP at different time points.
2. To assess cell-mediated immunogenicity by IFN-ɣ ELISPOT generated in individuals of heterologous prime-boost regime of ChAd63-MVA ME-TRAP at different time points.
3. To assess any differences in efficacy estimates with ID versus DVI challenge in individuals receiving R21/MM by assessing parasite density dynamics by qPCR.
4. To measure and compare the parasite growth rates and liver to blood inoculum in individuals receiving DVI versus ID sporozoite challenge in relation to naturally acquired immunity.

## 2.4. Tertiary objective(s)

To evaluate cell-mediated immunogenicity using flow cytometry with intracellular cytokine staining and other exploratory immunological end points.

# 3. TRIAL DESIGN

## 3.1. Overall Study Design and Plan Description

This is a Phase IIb Sporozoite Infection Study to assess the safety, immunogenicity and protective efficacy of 2 malaria vaccine candidate antigens: R21 adjuvanted with Matrix- M and ChAd63-MVA encoding ME-TRAP. It is an open-label, randomised controlled human malaria infection (CHMI) vaccine efficacy study. CHMI will be delivered either intradermally (ID) by inoculation of 22,500 PfSPZ Challenge or intravenously (DVI) by inoculation of 3,200 PfSPZ Challenge. The trial design is described in Table 3 below. We will recruit a total of 80 healthy adults who will be enrolled into 4 groups by randomisation (Table 3 and Figure 5). All eligible volunteers will be asked to attend on the day of enrolment including those in the control group where they will all be randomized to each respective group. Vaccination will be carried out over two days and the volunteers in the control group also spread over this time frame. Volunteers will further be sub-grouped into two cohorts per group who will be vaccinated and challenged sequentially over time. The sequence adopted will ensure that COVID-19 risk is mitigated. The vaccinations and CHMI studies will take place in Kilifi at KEMRI-CGMRC and Pwani University. Volunteers will be screened for significant medical conditions, including coronavirus disease 19 (COVID-19), before enrolling as detailed in section 3.1.2 below. CHMI, in a minimum number of 64 volunteers, will comprise theintradermal injection (ID) or the direct venous injection (DVI) of PfSPZ Challenge, which leadsto a blood-stage malaria infection after 6.5 days of incubation in the liver. Twice daily blood tests will be done to monitor the density of infection and anti-malarial treatment will be given either: a) when the density of infection rises past a threshold of 500 parasites per µl (a thresholdsubstantially lower than 2,500 parasites per µl at which clinical illness becomes more commonin children in Kenya [42]); b) if a volunteer develops symptoms or signs of illness and an immediate blood film examination shows evidence of detectable malaria parasites; or c) the volunteer reaches day 21 of monitoring, at which point CHMI will be completed or the participant is withdrawn. During the duration of CHMI, volunteers will be accommodated at afacility where they will stay for the entire duration of malaria infection. All volunteers will be provided with and required to use an ITN for the duration of the study.

**Table 3.** Trial groups and volunteer numbers.

| Week | 0 | 4 | 8 | 12 |
|---|---|---|---|---|
| **Group 1 N=24** | R21/ Matrix M 10µg /50µg | R21/ Matrix M 10µg /50µg | R21/ Matrix M 10µg /50µg | CHMI (ID) N=20* |
| **Group 2 N=24** | ChAd63 ME-TRAP $5x10^{10}$ vp | | MVA ME-TRAP $2x10^{8}$ pfu | CHMI (ID) N=20* |
| **Group 3 N=14** | R21/ Matrix M 10µg /50µg | R21/ Matrix M 10µg /50µg | R21/ Matrix M 10µg /50µg | CHMI (DVI) N=10* |
| **Group 4 N=18** | | | | CHMI (ID) N=14* |

Total of 80 volunteers, 62 of whom will be vaccinated with either R21/MM or ChAd63/MVA-ME-TRAP and 18 will be the control group receiving no vaccine. 64-80 volunteers will be challenged to account for any drop-out. ID refers to intradermal inoculation with 22,500 PfSPZ and DVI refers to direct intravenous inoculation with 3,200 PfSPZ. *indicates minimum number of volunteers required to be enrolled per group for CHMI.

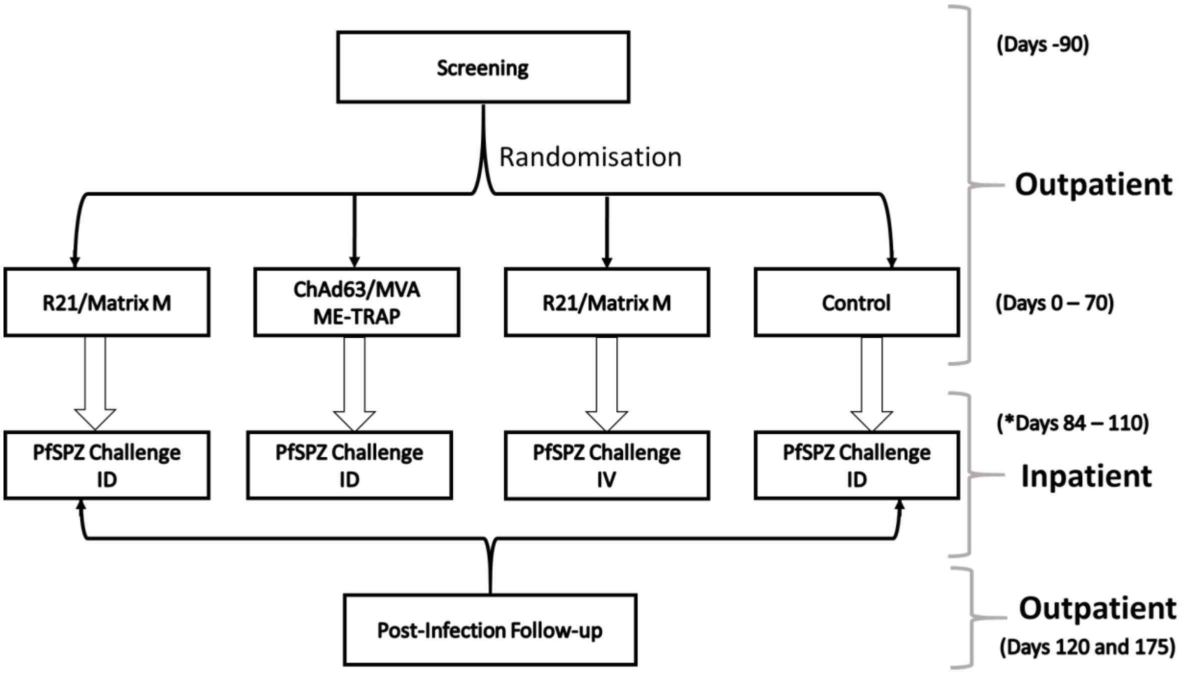


**Figure 5. Schematic of trial design.**
Healthy volunteers will be recruited from Ngerenya, Kilifi County and asked to attend a screening visit at KEMRI-CGMRC or Pwani University for informed consent processes and assessment of eligibility. A total of 80 eligible volunteers, who consent to participate in the study, will be asked to attend a pre-vaccination visit. Following from this they will all attend an enrolment visit where they will be randomized into one of 4 groups. 62 volunteers will be vaccinated with either R21/MM or ChAd63/MVA-ME-TRAP and 18 will be randomized to the control group receiving no vaccine. A minimum number of 64 volunteers will be administered with PfSPZ Challenge and followed up until endpoint and post-challenge follow up.

## Table 4. Procedures and blood volumes (ml) to be taken for volunteers per group

| Procedure/Visit | Group 1 | Group 2 | Group 3 | Group 4 |
|---|---|---|---|---|
| Screening | 8 | 8 | 8 | 8 |
| Pre-Vac | 37 | 37 | 37 | 37 |
| Vaccination visits[1] | 66 | 33 | 66 | - |
| Post-Vaccination | 98 | 68 | 98 | 8* |
| C-1 | 57 | 57 | 57 | 57 |
| PfSPZ Challenge | 238 | 238 | 238 | 238 |
| Post PfSPZ Challenge | 70 | 70 | 70 | 70 |
| **Total** | **574** | **511** | **574** | **418** |

Note: [1]there will be no blood draws conducted at enrolment visit (VAC1/E visit). *At day 70 (post-vaccination visit for vaccinated volunteers), the volunteers will be asked to attend a clinic visit where a 4ml blood sample will be taken to check for malaria by qPCR. If they are found to be positive, they will be administered with 7 day Artesunate monotherapy and a repeat blood sample after treatment completion for qPCR confirmation of clearance of parasites. These blood volumes are within the recommended national guidelines for blood draws. Additional bloods will be taken/repeated at any time if clinically indicated.

## Table 5: Schedule of procedures for R21/MM Vaccine Groups (1&3) before infection

| Event | S | Pre-VAC | VAC 1/E | Post VAC1 | Day 14 | VAC 2 | Post VAC2 | Day 42 | VAC 3 | Post VAC 3 | Day 70 | P | C-3 /C-1 |
|---|---|---|---|---|---|---|---|---|---|---|---|---|---|
| Attendance Number | 0 | 1 | 2 | 3-9 | 10 | 11 | 12-18 | 19 | 20 | 21-27 | 28 | 29 | 30 |
| Timeline (days) | -90 | -1 | 0 | 1-7 | 14 | 28 | 29-35 | 42 | 56 | 57-63 | 70 | 77 | 84 |
| Window (days) | | | | ±1 | ±2 | ±2 | ±1 | ±2 | ±2 | ±1 | ±2 | ±2 | 0 |
| ICF and ICF evaluation | X | | | | | | | | | | | | |
| Demography | X | | | | | | | | | | | | |
| Medical History | X | X | (X) | (X) | (X) | (X) | (X) | (X) | (X) | (X) | (X) | | X |
| Physical Examination | X | X | (X) | (X) | | (X) | | | (X) | (X) | (X) | | X |
| Anthropometry | X | X | | | | X | | | X | | | | X |
| Urinalysis | X | X | | | | | | | | | | | X |
| ECG | X | (X) | | | | | | | X | | | | X |
| β-HCG urine ♀ | X | X | | | | X | | | X | | | | X |
| Vital Signs* | X | X | X | (X) | | X | (X) | | X | (X) | | | X |
| Randomisation Feedback | | | X | | | | | | | | | | |
| Solicited AEs | | | | X | | | X | | | X | | | |
| Unsolicited AEs | | | | X | X | X | X | X | X | X | X | | X |
| Safety Bloods** | 3 | 3 | | | | 3 | | | 3 | | | | 3 |
| Serology*** | 1 | | | | | | | | | | | | |
| Immunology Bloods | | 30 | | | 30 | 30 | | 30 | 30 | | 30 | | 50 |
| Malaria PCR | 4 | 4 | | | | | | | | | 4 | 4 | 4 |
| COVID-19 PCR$ | | | | | | | | | | | | Y | Y |
| Total Blood volume (ml) | 8 | 37 | 0 | 0 | 30 | 33 | 0 | 30 | 33 | 0 | 34 | 4 | 57 |
| Cumulative blood volume (ml) | 8 | 45 | 45 | 45 | 75 | 108 | 108 | 138 | 171 | 171 | 205 | 209 | 266 |

Note: S refers to screening; VAC refers to vaccination; C refers to PfSPZ Challenge administration; (X) if clinically indicated; ECG, Electrocardiogram; β-HCG, beta-Human Chorionic Gonadotrophin; and P refers to post-treatment qPCR check where applicable. *Vital signs include pulse, blood pressure and temperature; **Safety bloods: Haematology will include a complete blood count, biochemistry will include Sodium, Potassium, Urea, Creatinine, Albumin, ALT and bilirubin. ***Serology includes HIV, HCV and HBsAg antibody testing. Post vaccination monitoring will be after every vaccination visit for a period of 7 days post vaccination with self-reporting to the clinical team of any unsolicited AEs. Should there be any medical concerns the clinician will review the participant and record any vital signs related to the visit. Y indicates time points when nasal and/or oral swab will be taken for COVID-19 PCR testing. At visit P (day 77, all volunteers will undergo COVID-19 PCR testing and those that are negative will be enrolled as residents at Pwani University. At C-3 volunteers will undergo a repeat nasal and/or oral swab to test for COVID-19 PCR testing prior to challenge which will allow for repeat testing in case of discrepancy or clarification of results. $COVID-19 PCR tests will be repeated at any time if clinically indicated.

## Table 6: Schedule of procedures for Group 2 ChAd63/MVA-ME-TRAP before infection

| Event | S | Pre-VAC | VAC 1/E | Post VAC1 | Day 14 | VAC 2 | Post VAC2 | Day 63 | Day 70 | P | C-3 /C-1 |
|---|---|---|---|---|---|---|---|---|---|---|---|
| Attendance Number | 0 | 1 | 2 | 3-9 | 10 | 11 | 12-18 | 18 | 19 | 20 | 21 |
| Timeline (days) | -90 | -1 | 0 | 1-7 | 14 | 56 | 57-63 | 63 | 70 | 77 | 84 |
| Window (days) | | | | ±1 | ±2 | ±2 | ±1 | ±2 | ±2 | ±2 | 0 |
| ICF and ICF evaluation | X | | | | | | | | | | |
| Demography | X | | | | | | | | | | |
| Medical History | X | X | (X) | (X) | (X) | (X) | (X) | (X) | (X) | | X |
| Physical Examination | X | X | (X) | (X) | | (X) | | (X) | (X) | | X |
| Anthropometry | X | X | | | | X | | | | | X |
| Urinalysis | X | X | | | | | | | | | X |
| ECG | X | (X) | | | | | | | | | X |
| β-HCG urine ♀ | X | X | | | | X | | | | | X |
| Vital Signs* | X | X | X | X | | X | X | | | | X |
| Randomisation Feedback | | | X | | | | | | | | |
| Solicited AEs | | | | X | | | X | | | | |
| Unsolicited AEs | | | | X | X | X | X | X | X | | X |
| Safety Bloods** | 3 | 3 | | | | 3 | | | | | 3 |
| Serology*** | 1 | | | | | | | | | | |
| Immunology Bloods | | 30 | | | 30 | 30 | | 30 | | | 50 |
| Malaria PCR | 4 | 4 | | | | | | | 4 | 4 | 4 |
| COVID-19 PCR$ | | | | | | | | | | Y | Y |
| Total Blood volume (ml) | 8 | 37 | 0 | 0 | 30 | 33 | 0 | 30 | 4 | 4 | 57 |
| Cumulative blood volume (ml) | 8 | 45 | 45 | 45 | 75 | 108 | 108 | 138 | 142 | 146 | 203 |

Note: S refers to screening; VAC refers to vaccination; C refers to PfSPZ Challenge administration; (X) if clinically indicated; ECG, Electrocardiogram; β-HCG, beta-Human Chorionic Gonadotrophin; and P refers to post-treatment qPCR check where applicable. *Vital signs include pulse, blood pressure and temperature; **Safety bloods: Haematology will include a complete blood count, biochemistry will include Sodium, Potassium, Urea, Creatinine, Albumin, ALT and bilirubin. ***Serology includes HIV, HCV and HBsAg antibody testing. Post vaccination monitoring will be after every vaccination visit for a period of 7 days post vaccination with self-reporting to the clinical team of any unsolicited AEs. Should there be any medical concerns the clinician will review the participant and record any vital signs related to the visit. Y indicates time points when nasal and/or oral swab will be taken for COVID-19 PCR testing. At visit P (day 77, all volunteers will undergo COVID-19 PCR testing and those that are negative will be enrolled as residents at Pwani University. At C-3 volunteers will undergo a repeat nasal and/or oral swab to test for COVID-19 PCR testing prior to challenge which will allow for repeat testing in case of discrepancy or clarification of results. $COVID-19 PCR tests will be repeated at anytime if clinically indicated.

## Table 7: Schedule of events for CHMI (all volunteer groups – 1 to 4)

| Timeline (days in relation to challenge) | C | C+1-21 | Diagnosis | +24hr | +48hr | +72hr | C+35 | C+90 |
|---|---|---|---|---|---|---|---|---|
| Window (days) | 0 | 0 | 0 | 0 | 0 | 0 | ±5 | ±5 |
| Clinical assessment | X | X | X | X | X | X | X | X |
| Vital signs^ | X | X | X | X | X | X | X | X |
| Urinary B-HCG | | | X | | | | | |
| Anthropometry | | | X | | | | | |
| Malaria PCR | | X | X | X | X | X | | |
| COVID-19 PCR | | X | | | | | | |
| Haematology% | | X | | | | X | X | X |
| Biochemistry* | | X | X | | | | | |
| Immunology bloods | | X | X | | | | X | X |
| Administration of PfSPZ Challenge | X | | | | | | | |
| Local and systemic AEs Reviewed | X | X | X | X | X | X | X | X |
| Anti-malarial directly Observed | | | X | X | X | X | | |

C refers to PfSPZ Challenge; C-1 day before PfSPZ Challenge; C+1 – C+21, C+35, and C+90 days refers to time points after challenge; +24hr, +48hr, and +72hr refers to time points after malaria diagnosis and anti-malaria treatment. B-HCG, beta-Human Chorionic Gonadotrophin. Clinical assessment will include physical examination and be based on tests conducted from blood samples collected. ^Vital signs includes pulse, blood pressure and temperature. % Haematology will include a full blood count. *Biochemistry will include Sodium, Potassium, Urea, Creatinine, Albumin, ALT and bilirubin. A nasal and/or oral swab will be taken for COVID-19 PCR testing.

For Group 4, they will undergo screening activities at the screening visit together will all the other volunteers. At their screening visit, they will go through the following procedures: ICF and ICF evaluation; medical history; clinical assessment; anthropometry, urinalysis; ECG; β-HCG urine (♀); vital signs; safety bloods; serology; and malaria qPCR as proposed for groups 1, 2, and 3. A 8ml blood sample will be collected at the screening visit (safety bloods, 3ml; serology, 1ml; and malaria qPCR, 4ml). They will be required to attend the pre-vaccination visit (Pre-Vac visit) where, a 37ml blood sample will be taken for safety bloods, 3ml; immunology, 30ml; and malaria qPCR, 4ml. They will also be required to attend the enrolment visit (VAC 1/E visit) where they will be randomised together with all the other volunteers to their respective group. At day 70, the volunteers will be asked to attend a clinic visit where a 4ml blood sample will be taken to check for malaria by qPCR. If they are found to be positive for malaria, they will be administered with 7-day Artesunate monotherapy and a repeat blood sample after treatment completion for qPCR confirmation of clearance of parasites. In addition, a nasal and/or oral swab will be taken from the volunteers at day 77 for COVID-19 PCR testing. If they are found to be positive for COVID-19, they will be referred to the Kilifi County COVID-19 Response Team (KCCRT) for management. For those negative, they will be enrolled as residents at Pwani University.

At C-3, group 4 volunteers will have a nasal and/or oral swab sample taken for COVID-19 PCR testing. If they are found to be positive for COVID-19, they will be referred to the Kilifi County COVID-19 Response Team (KCCRT) for management. For those negative, they will continue their residence at Pwani University.

At C-1, eligible group 4 volunteers will undergo the same procedures carried out at screening with the following exceptions: serology and ECG. At C-1 a blood sample of 57ml will be taken (safety bloods, 3ml; immunology bloods, 50ml; and malaria qPCR, 4ml). The cumulative blood volume for group 4 at C-1 will be a total of 108ml.

**Table 8. Schedule of venipuncture after challenge and study exit.**

| Days post Challenge | 5 | 7 | 7.5 | 8 | 8.5 | 9 | 9.5 | 10 | 10.5 | 11 | 11.5 | 12 | 12.5 | 13 | 13.5 | 14 | 14.5 | 15 | 16 | 17 | 18 | 19 | 20 | 21 | Diag& | +24hr | +48hr | +72hr | C+35 | C+90 |
|---|---|---|---|---|---|---|---|---|---|---|---|---|---|---|---|---|---|---|---|---|---|---|---|---|---|---|---|---|---|---|
| PCR | | 4 | 4 | 4 | 4 | 4 | 4 | 4 | 4 | 4 | 4 | 4 | 4 | 4 | 4 | 4 | 4 | 4 | 4 | 4 | 4 | 4 | 4 | 4 | 4 | 4 | 4 | 4 | 4 | 4 |
| Haematology | | 1 | | | | | | | | | | | | | | 1 | | | | | | | | 1 | 1 | | | 1 | 1 | 1 |
| Biochemistry*^ | 2 | 2 | | | | 2 | | | | | | | | | | | | | | | | | | 2 | 2 | | | | | |
| Plasma/PBMCs$ | 30 | 30 | | | | 30 | | | | | | | | | | | | | | | | | | 30 | 30 | | | | 30 | 30 |
| Parasite Typing | | | | | | | | | | | | | | | | | | | | | | | | 2 | 2 | | | | | |
| Vol (ml) | 32 | 37 | 4 | 4 | 4 | 36 | 4 | 4 | 4 | 4 | 4 | 4 | 4 | 4 | 4 | 5 | 4 | 4 | 4 | 4 | 4 | 4 | 4 | 39 | 39 | 4 | 4 | 5 | 35 | 35 |
| Cumulative total (ml) | 32 | 69 | 73 | 77 | 81 | 117 | 121 | 125 | 129 | 133 | 137 | 141 | 145 | 149 | 153 | 158 | 162 | 166 | 170 | 174 | 178 | 182 | 186 | 225 | - | 229 | 233 | 238 | 273 | 308 |

Note: Only one blood sample will be drawn between day of Challenge and Challenge +6 (C+5). *Biochemistry will include Sodium, Potassium, Urea, Creatinine, Albumin, ALT and bilirubin. ^Blood will be drawn for biochemistry on +9, C+21 and day of diagnosis. $Blood will be taken for plasma and/or PBMCs on +5, +7, +9, +21, and day of diagnosis and then not again until Challenge +35. &Blood sample will not be taken at diagnosis if a sample has already been taken on the same day. The cumulative total includes only blood volumes from C+5 to C+90 after malaria infection. The cumulative total blood volumes including screening, pre-vaccine, pre-malaria infection, and post-malaria infection, whereapplicable, are indicated in Table 4.

### 3.1.1. Recruitment

The target volunteer population will be healthy, adult volunteers. Recruitment will be conducted by a team of fieldworkers from CGMR-C targeting residents of the Kilifi Health Demography Surveillance System. Recruitment will target residents within the Ngerenya sub-location of Kilifi County. Community engagement meetings will be undertaken with COVID-19 guidelines to minimise risk of infection transmission. The nature of the meetings will involve use of engagement strategies that have proved to be effective during the pandemic ensuring the necessary preventative measures are followed for example use of personal protective equipment (PPE), physical distancing, and other prevention and control measures (e.g. telephone, door-to-door recruitment by trained field workers and small meetings). These meetings to inform potential volunteers will be conducted for residents in Ngerenya. We will also approach potential volunteers who have taken part in previous screening events under the protocol KEMRI/SERU/CGMR-C/029/3190 but were not enrolled for PfSPZ Challenge administration. For those who are willing to participate in the study, they will be asked to travel to CGMR-C or Pwani University where the consent process, screening and enrolment into the study will take place. Investigators will emphasize that participation in the study is voluntary. Information sheets will be given to interested volunteers. Individuals who feel that the trial is appropriate for them will be invited to attend a formal screening visit with a study clinician. Potential participants will be screened for good health to identify the minimum number required for each group, we will screen potential volunteers until we reach the number required for enrolment. It will be made clear to all potential participants that they may be excluded for several health reasons even for conditions that may have relatively little impact on their day to day lives. The trained study team members such as field workers will conduct individual meetings with the participants to ensure understanding of the information sheet and consent form.

### 3.1.2. Screening

All potential volunteers will have a screening visit at CGMR-C or Pwani University which may take place up to 90 days prior to enrolment. Information regarding the study will be disseminated either on an individual basis or in a small group session where potential participants will be encouraged to ask questions. After this session, each participant will have a private discussion with trained study staff where additional clarification will be provided and time for more questions provided. Written informed consent will be taken before screening. If consent is obtained, the screening procedures indicated in the schedule of procedures (Tables 6 & 7) will be undertaken. After consent is obtained, each consented participant will be screened for clinically significant acute or chronic diseases based on the inclusion and exclusion criteria. This will be done using both general physical examination and screening laboratory tests; complete blood count (CBC), HIV, Hepatitis B and C, malaria, and biochemistry. Likelihood of migration will be an important factor for non-enrolment. Abnormal clinical findings from the medical history, clinical assessment, or blood tests at any point in the study will be assessed. If a test is deemed clinically significant it may be repeated to ensure it is not a single occurrence. If an abnormal finding is deemed to be clinically significant, the volunteer will be informed and referral to an appropriate medical centre arranged with the permission of the volunteer. Decisions to exclude the volunteer from enrolling in the trial or to withdraw a volunteer from the trial will be at the discretion of the Investigator. Volunteers will undergo counselling prior to HIV test. HIV sero-status will be established using the standard rapid diagnostic kits in the lab as per the testing algorithm used by the Kenyan Ministry of Health. Those diagnosed as HIV positive will be referred to an appropriate health centre for further counselling and treatment. To maintain confidentiality of those volunteers infected with HIV, we will make it

clear during screening that one can be excluded due to a range of diseases (not just HIV) as well as abnormal laboratory results.

Once all specific visit procedures are completed, results will be fed back in a timely manner before the Pre-Vac visit (day prior to proposed 1st vaccination). The screening laboratory results will be reviewed by the study clinicians before the participants are next seen. These procedures will be documented in the electronic case report forms (eCRFs)/paper CRF and relevant clinical notes, which will be kept in the individual study participant's file.

### 3.1.3. Enrolment

Participants will be considered enrolled at the time of randomisation and vaccination or equivalent. Enrolment will take place within 90 days of screening. Where this time frame is exceeded volunteers will need to be re-screened. We will enrol a total of 80 participants for vaccination from which 62 will be enrolled to receive vaccinations prior to CHMI, 18 will form part of the control group and a minimum number of 64 participants will be enrolled for CHMI. All volunteers will give written informed consent before being enrolled, after having been informed of the nature of the trial, the potential risks and their obligations. All screened volunteers will be informed of results of laboratory tests and their eligibility for enrolment after the screening visit. The volunteers will then be randomly assigned; randomisation will be via a computer-generated sequence by an independent statistician. A randomisation list in the form of password protected spreadsheet will be generated using STATA/R and the data manager/designee will set up randomisation in REDCap. Study clinicians will only click to randomise after confirmation of eligibility criteria and immediately prior to vaccination for each volunteer, after which REDCap will reveal the participants' randomisation arm that cannot be edited. The volunteers will be randomised to either one of four groups. Volunteers will be assigned their randomisation group at the vaccination/enrolment visit.

### 3.1.4. Study Procedures

Procedures will be performed at the time points indicated in the schedule of procedures in Tables 5 – 8 above and Figure 5. Additional procedures or laboratory tests may be performed at the discretion of the clinical team and investigators if clinically necessary (e.g. urine microscopy in the event of positive urinalysis). Abnormal clinical findings from the medical history, clinical assessment or clinically-relevant blood tests at any point in the study will be reviewed by a qualified clinician and acted on appropriately (including repeated or further testing if necessary). If an abnormal finding is deemed to be clinically significant, the participant will be informed and referral to an appropriate government medical centre will be arranged in consultation with the participant. Initial referrals and transport will be facilitated by the study team. Results may be repeated for verification but sample volumes obtained will remain within the WHO recommendations (41).

**1. Observations**

Full physical examination including vital signs: pulse, blood pressure, respiratory rate, and temperature will be measured at the time points indicated in the schedule of procedures. (Tables 5-8)

**2. Laboratory Tests**

Blood will be drawn at the time points indicated in Tables 5 – 8 and the following laboratory assays performed as indicated in the tables:

a) Haematology: Complete Blood Count (CBC).
b) Biochemistry: Sodium, Potassium, Urea, Creatinine, Albumin, ALT and bilirubin.
c) Diagnostic serology: HIV antibodies, Hepatitis B, and Hepatitis C.
d) Immunological assays to determine naturally acquired immunity to malaria: antibody and cell-mediated immunity. Protein arrays will be used to quantify antibodies against parasite antigens whilst antibody-dependent assays of functional immunity will also be performed such as growth inhibition assays (GIAs), opsonic phagocytosis assays(OPA), and antibody dependent respiratory burst (ADRB), complement-fixation assays and other assays that will be developed using plasma.
e) Diagnostic Malaria Tests: Primarily PCR for *P. falciparum* DNA and microscopy, and for detection of parasite sexual stages using a reverse transcriptase PCR assay.
f) DNA Genotyping PCR: For red blood cell polymorphisms including but not limited to sickle cell trait, alpha-thalassemia, Dantu mutation of glycophorin A, and others that might affect susceptibility or resistance to malaria.

The haematology, biochemistry and diagnostic work will be done on site (at KEMRI-CGMR-C) or an accredited laboratory. Immunological assays and DNA based PCR will be conducted at CGMR-C. For asexual stage based PCR, additional testing and quality control may require shipping to the Jenner Institute, University of Oxford, UK; to Mahidol Oxford Research Unit, Mahidol University, Bangkok, Thailand; and University of Washington, Seattle, USA. In addition, plasma samples will be tested for anti-malarial drug levels a day before malaria infection and when asexual blood-stage parasites are detectable in the blood stream. This will require samples to the tested at the Mahidol Oxford Research Unit, Mahidol University, Bangkok, Thailand and thus samples would need to be shipped. The need for the shipment of the samples is to utilise techniques (such as ultra-sensitive qPCR parasite detection and ultra-sensitive pharmacological drug screening assays) that are currently not available in Kenya.

Nasal and/or oral swab samples will be taken from each participant as indicated in Tables 5 – 7 for the detection of SARS-CoV-2 the pathogen that causes COVID-19 by PCR testing.

**3. Urinalysis**
Urine will be tested for the presence of clinically significant proteinuria, glucosuria or haematuria at screening and at various follow-up time points. Urine will be tested for β-HCG in female volunteers at screening, prior to administration of PfSPZ Challenge and prior to start of anti-malarial medication.

**4. ECG**
Electrocardiograms (ECGs) will be performed at screening and examined by a clinically qualified investigator for evidence of significant heart disease.

#### 3.1.4.1. Pre-Vaccination (Pre-Vac) Visit

Participants who meet the inclusion criteria at the screening visit will be seen a day prior to first vaccination/enrolment. A clinical assessment will be conducted to review any new medical complaints since their screening visit. The clinical assessment will include a repeat height and weight, temperature, BP and heart rate, venepuncture for repeat basic haematology and biochemistry and blood tests for baseline immunology (see Tables 5-8), malaria PCR, urinalysis and urinary β-HCG for females. In addition, anti-circumsporozoite antibody levels and associated cellular immune responses will be assessed. There will also be a review of any

concomitant medication. For participants whose screening assessments lead to exclusion this visit will serve as a feedback visit to relay reasons for exclusion and further referral if necessary.

#### 3.1.4.2. Enrolment Visit (VAC 1/E)

All results from the pre-vaccination visit will be reviewed prior to enrolment and only participants who meet the eligibility criteria will be enrolled. If eligible, an enrolment visit will be scheduled for the participant to receive the vaccine. Participants will not be considered enrolled in the study until they have been randomised into one of 4 groups and received a vaccine where applicable. Vaccination will be supervised by a trained clinician with appropriate resuscitation equipment and drugs available. If the participant is considered ineligible due to results taken from investigations at the pre-vaccination visit, this will be communicated at this visit. The following procedures will be performed during each enrolment visit:

- Targeted medical history since previous visit
- Review of inclusion / exclusion criteria
- Vital signs (HR, BP)
- Pre-vaccination axillary temperature
- Randomisation

All participants, where applicable, will be assessed 60 minutes after vaccination as detailed below to evaluate and treat any adverse events.

- Record vital signs (HR, BP)
- Record any post-vaccination solicited adverse events (AEs).
- Record any post-vaccination unsolicited (AEs).
- Record any post-vaccination serious adverse events (SAEs).
- Provide appointment and emergency contact card

#### 3.1.4.3. Post-Vaccination Monitoring Visits

- **Post VAC1, VAC2, and VAC3**

**Groups 1 and 3: V3-9; V12-18; and V21-27**
**Group 2: V3-9; V12-18**
After each vaccination visit unsolicited AEs will be documented by the clinical team. This will be done for a period of 7 days' post-vaccination. If deemed clinically necessary, the volunteer will attend a clinic visit and continue to be seen regularly until the AEs have resolved or stabilized. Should there be any medical concerns, the study clinicians will review the volunteer and the following will be assessed and recorded in the CRF:

- Record concomitant medication taken.
- Record any post-vaccination solicited adverse events (AEs).
- Record any post-vaccination unsolicited AEs.
- Record any post-vaccination serious adverse events (SAEs).
- Vital signs
- Physical examination will be conducted

- **Other Visits**

**Group 1 and 3: V10; V19; and V28 [Days 14, 42, and 70]**
**Group 2: V10; and V19 [Days 14 and 63]**
Volunteers will be asked to attend the clinic where the following procedures will be conducted:

- Immunology bloods

- Record any post-vaccination unsolicited SAEs

On day 70, all volunteers including the control group will attend the clinic for a qPCR blood test. This will allow sufficient time for diagnosis of malaria and treatment prior to CHMI if necessary. Those who will be found positive, will be administered with a 7-day Artseunate monotherapy and a repeat blood sample at day 77 (event P) for confirmation of parasite clearance (see section 3.1.4.5. below). At day 77 (event P) all volunteers, including those who were administered with 7-day Artesunate monotherapy, will be asked to attend a clinic visit and a nasal and/or oral swab taken for COVID-19 PCR testing. Those found to be COVID-19 negative, will be enrolled as residents at Pwani University and those positive referred to the RRT for management.
All information gathered will be entered on the participant's eCRF/paper CRF.

#### 3.1.4.4. Booster Vaccination Visits

**Groups 1 and 3: V11 and V20 [Days 28 and 56]**
**Group 2: V11 [Day 56]**
The following procedures will be conducted:

- Carry out physical examination if clinically indicated
- Height and Weight
- Vital signs (HR, BP)
- Check exclusion criteria
- Record/review unsolicited adverse events and SAEs experienced by the participant since the last visit.
- Record pre-vaccination axillary temperature.
- CBC and Biochemistry
- Urine sample collection for the determination of pregnancy (adult females only). The test results must be negative prior to administration of the vaccine
- Immunology bloods (section 9.3)
- Administer the 2nd dose of R21/MM **(Groups 1 and 3)**
- Administer the 3rd dose of R21/MM **(Groups 1 and 3)**
- Administer MVA ME-TRAP **(Group 2)**
- Record any post-vaccination solicited AEs.
- Record any post-vaccination unsolicited AEs.
- Record any post-vaccination SAEs.
- Record concomitant medication.

#### 3.1.4.5. Malaria Infection Prior to PfSPZ Challenge Administration

To prevent pre-existing malaria infection from interfering with the study, volunteers who are found to be qPCR positive for malaria infection will be treated with 7 days of Artesunate (this drug will be chosen to avoid other drugs with long half-lives that might interfere with subsequent CHMI), and the volunteer will then be screened again by PCR to confirm that they are negative in the week before CHMI.

#### 3.1.4.6. Dose and Route of PfSPZ Challenge

Administration of PfSPZ Challenge will be via direct venous inoculation (3,200) or intradermally (22,500). This will be done by a trained clinician. The dose and route of administration of PfSPZ Challenge to be used in this trial have been chosen to maximise the likelihood of successful infection with malaria and are based on safety data from previous

clinical trials of PfSPZ Challenge in malaria naïve individuals and in malaria endemic regions (see background section above).

### 3.1.4.7. Preparation and Administration of PfSPZ Challenge

Immediately prior to use, PfSPZ Challenge in cryovials will be thawed individually by partial submersion of the vials for 30 seconds in a 37°C ± 1°C water bath. Designated, trained study staff will then prepare, dilute (if necessary) and dispense PfSPZ Challenge to clinical staff at the clinical study site according to the appropriate SOP. Aliquots of the diluents phosphate buffered saline (PBS) and 25% human serum albumin (HSA) will be provided to the clinical sites by Sanaria Inc. PfSPZ Challenge will be administered using a needle and syringe by DVI or ID according to the appropriate SOPs. Study staff administering PfSPZ Challenge will use standard universal protection (aseptic technique and gloves) as well as eye protection.

### 3.1.4.8. Day Before PfSPZ Challenge (C-1)

All volunteers (vaccinees and controls, groups 1 to 4) will attend a pre-challenge clinic visit. The following procedures will be conducted:

- Assessment of any new medical issues or symptoms that have arisen since screening
- Height and Weight
- Vital signs (HR, BP)
- Check exclusion criteria
- Record axillary temperature.
- CBC and Biochemistry (rresults of bloods taken at this visit must be available and reviewed prior to PfSPZ Challenge administration)
- Immunology bloods
- Malaria qPCR blood test
- COVID-19 PCR test (from a nasal and/or oral swab)
- Urine sample collection for determination of pregnancy (adult females only). The test results must be negative prior to administration of PfSPZ Challenge.

### 3.1.4.9. Day of PfSPZ Challenge administration

All volunteers will have clinical assessments performed prior to challenge. If withdrawal criteria are met this will be dealt with as described in section 4.5. below.

### 3.1.4.10. Monitoring of CHMI

All volunteers will remain at Pwani University, in Kilifi until completion of anti-malarial treatment. All volunteers will be reviewed and have observations performed twice daily and more frequently if a volunteer is symptomatic. Full contact details for each subject will be documented, including home address, home and work land-line telephone numbers where available, and next-of-kin address and telephone numbers. Any AEs occurring post administration of PfSPZ Challenge (solicited and unsolicited) will be documented.

### 3.1.4.11. Days 1-6 Post PfSPZ Challenge (C+1 – C+6)

The liver stage of malaria infection is asymptomatic and lasts 6 days. A blood sample will only be collected on day 5 post-challenge (C+5) where 32ml of venous blood sample will be taken for assessment of liver stage immunity and safety bloods (biochemistry). During this period volunteers will be required to be resident at Pwani University and will have access to a study clinician in case of any symptoms.

#### 3.1.4.12. Days 7-21 Post PfSPZ Challenge (C+7 – C+21)

The following procedures will be conducted at each review:

- Clinical assessment
- Record any adverse events related to symptoms of malaria (See Table 11)
- Safety bloods as per schedule in Table 8
- Malaria PCR (Venous blood samples (each 4ml) will be taken for PCR for *P. falciparum* twice per day (i.e. morning between 7 am and 10am, and evening between 3pm and 7pm) from days 7 to 14, and then once per day from 15 to 21. PCR results willbe processed within 6 hours of collection for the morning tests and within 18 hours forthe evening tests. Anti-malarials will be given at a threshold of 500 parasites per µl.)
- If a volunteer develops symptoms and signs of malaria, then a rapid diagnostic test (RDT) will be done and a sample taken for an immediate blood film examination to be conducted and results relayed immediately on availability. The presence of a parasite will be confirmed by a second microscopist before the film is considered positive. If any parasites are seen on the blood film then anti-malarials will be given.
- If the clinical investigators have concern regarding the clinical condition of any volunteer, they may advise treatment with anti-malarials irrespective of the results of PCR or microscopy.
- When a case of malaria is diagnosed, each subject will have a clinical evaluation by (a clinician with appropriate history and clinical examination.
- If in the opinion of a clinical investigator a volunteer shows signs that indicate the need for in-patient care, then admission will be organized to an appropriate hospital in Kilifi or Mombasa. If intensive care facilities required, the referral will be to an appropriate facility.
- In the event that a volunteer develops any signs and symptoms of COVID-19 during the stay in Pwani then in the first instance a nasal and/or oral swab will be taken for rapid diagnostic COVID-19 testing (if available) and results relayed immediately. If the volunteer is positive (or the rapid test is not available) then they will remain in their room under strict isolation pending confirmatory PCR tests. A sample will also be taken for PCR testing.
- If a volunteer is found to be positive for COVID-19, they will be referred to the Kilifi County COVID-19 Response Team (KCCRT) for management and initiated on endpoint anti-malaria treatment. The study team will support KCCRT management regarding any needed advice or input on malaria treatment.
- If there is an isolated fever or temperature in a volunteer known to have a malaria parasite count above 50 per ml, then this will be assumed to be due to malaria and all necessary procedures for malaria outlined above will be followed. COVID-19 testing will be undertaken if there are additional suggestive features.
- Immunology bloods at 3 time points, (days 7, 9, and 21 or day of diagnosis if earlier).

### 3.1.5. Unscheduled Visits

If the participant is unwell, they will be strongly encouraged to seek treatment with the study team and given emergency contact numbers to facilitate this. Out of hours' participants will be advised to contact the study clinician and in the event of an emergency, to attend Kilifi County Hospital. Medical emergency plans will be in place with the possibility to admit patients to the Aga Khan Hospital in Mombasa, should intensive care be required. Study participants referred to other hospital will be managed based on hospital and/or national guidelines. The study team

will track clinical progress and outcomes for all admitted participants and remain in touch with participants where feasible.

#### 3.1.6. End of Treatment Visit (Post diagnosis follow-up (+24hr, +48hr, +72hr)

Volunteers will continue to be reviewed and have clinical observations performed once a day post diagnosis. If qPCR results from blood taken at 24, 48 and 72 hours post diagnosis are negative for parasites and the patient has no symptoms or mild, resolving symptoms, then the volunteer will be able to leave Pwani University and not reviewed again until Day 35 post-infection (C+35). If further blood samples are positive or symptoms are persistent then a medical assessment will be conducted, and further investigation or treatment planned according to the findings. 1ml of blood will also be drawn 72 hours' post-diagnosis for full blood count (Table 8) for safety monitoring.

#### 3.1.7. Follow-up Visits: C+35 and C+90

All volunteers will be reviewed in the clinic 35 and 90 days after PfSPZ Challenge. Clinical assessments will be performed, and AEs assessed. Venipuncture will be performed (Table 8).

#### 3.1.8. Study Restrictions

Safety oversight will be the responsibility of the investigators, Local safety monitor (LSM), and the independent Data Safety and Monitoring Board (DSMB).

***Discontinuation of the study***

The study in its entirety may be discontinued prematurely by the PI, Sponsor, or the Ethics Committee with oversight responsibilities at any time, and/or individual subjects may terminate their participation prematurely, or have their participation be terminated by the Investigator. All arms of the study will be discontinued in the event of the following:

1. New scientific information is published to indicate that the participants in the study are being exposed to undue risks because of administration of investigational medicinal products (IMPs) or as a result of the follow –up schedule.
2. Serious concern about the safety of the IMPs arise because of vaccine related serious adverse event (SAE)s occurring in the subjects enrolled in this or any other ongoing study of the IMPs.
3. For any other reason at the discretion of the Principal investigators.
4. Should there be safety concerns raised by the authorising ethics committees (SERU & OxTREC).

***Stopping rules***

The study will be placed on safety hold if:

- One or more participants experience any SAE related to the study product (probable or related)
- A suspected unexpected serious adverse drug reaction (SUSAR) occurs.

## 4. SELECTION AND WITHDRAWAL OF STUDY PARTICIPANTS

### 4.1. Inclusion criteria

The participant must satisfy all the following criteria to be eligible for the study:

- Healthy adults aged 18 to 45 years
- Able and willing (in the Investigator's opinion) to comply with all study requirements
- Non-pregnant, non-lactating adult female or adult male
- Agreement to refrain from blood donation during the study
- Use of effective method of contraception for the duration of study for female participants. For those with no contraception, they will be referred for contraception at the relevant

health facility. For female participants, we will ask them to attend with their family planning records for verification. Effective contraception is defined as a contraceptive method with failure rate of less than 1% per year when used consistently and correctly, in accordance with the product label. Examples of these include: combined oral contraceptives; injectable progestogen; implants of etenogestrel or levonorgestrel; intrauterine device or intrauterine system; male condom combined with a vaginal spermicide (foam, gel, film, cream or suppository); and male condom combined with a female diaphragm, either with or without a vaginal spermicide (foam, gel, film, cream, or suppository)

- Provide written informed consent
- Plan to remain resident in the study area for 1 year following first dose of vaccination

### 4.2. Exclusion criteria

- Clinically significant congenital abnormalities as judged by the study clinicians
- Any confirmed or suspected immunosuppressive or immunodeficient state, including HIV infection; asplenia; recurrent, severe infections and chronic (more than 14 days) immunosuppressant medication within the past 6 months (inhaled and topical steroids are allowed).
- Sickle cell disease
- Any history of anaphylaxis in relation to vaccination
- Clinically significant laboratory abnormality as judged by the study clinician
- Blood transfusion within one month of enrolment
- Haemoglobin less than 11.3 g/dl for men and less than 10g/dl for in women, where judged to be clinically significant in the opinion of the investigator.
- Administration of immunoglobulins and/or any blood products within the three months preceding the planned administration of the vaccine candidate
- Participation in another research study involving receipt of an investigational product in the 30 days preceding enrolment, or planned use during the study period
- Seropositive for hepatitis B surface antigen (HBsAg) or hepatitis C (HCV IgG)
- Use of systemic antibiotics with known antimalarial activity within 30 days of administration of PfSPZ Challenge (e.g. trimethoprim-sulfamethoxazole, doxycycline, tetracycline, clindamycin, erythromycin, fluoroquinolones and azithromycin)
- Women only; pregnancy, or an intention to become pregnant a day before challenge i.e. at C-1
- Any significant disease, disorder or situation (including confirmed COVID-19 PCR positivity) which, in the opinion of the Investigator, may either put the participants at risk because of participation in the trial, or may influence the result of the trial, or the participant’s ability to participate in the trial
- Confirmed parasite positive by PCR a day before challenge i.e. at C-1.
- Confirmed PCR positive for COVID-19 three days before challenge i.e. C-3.

### 4.3. Exclusion criteria on day of challenge

- Acute disease, defined as moderate or severe illness with or without fever (temperature >37.5°C)

### 4.4. Indications for delayed vaccination

The following adverse events constitute contraindications to administration of vaccine at that point in time; if any one of these adverse events occurs at the time scheduled for vaccination, the subject may be vaccinated later, or withdrawn at the discretion of the investigator. The subject must be followed until resolution or stabilization of the adverse event or until causality is determined to be unrelated to trial interventions, as with any adverse event.

- Acute disease at the time of vaccination. (Acute disease is defined as the presence of moderate or severe illness with or without fever). Vaccines can be administered to persons with a minor illness such as diarrhoea, mild upper respiratory infection with or without low-grade febrile illness, i.e., temperature of <37.5°C/99.5°F).
- Temperature of ≥37.5°C (99.5°F) at the time of vaccination

**Note:** Anaphylactic reaction following administration of study vaccine constitutes an absolute contraindication to further administration of vaccine, and the subject must be withdrawn and followed until resolution of the event

### 4.5. Withdrawal criteria

In accordance with the principles of the current revision of the Declaration of Helsinki (updated 2008) and any other applicable regulations, a volunteer has the right to withdraw from the study at any time and for any reason and is not obliged to give his or her reasons for doing so. The Investigator may withdraw the volunteer at any time in the interests of the volunteer's health and well-being. In addition, the volunteer may withdraw/be withdrawn for several reasons including those listed below:

- On the decision of the investigator
- On the advice of data safety and monitoring board (DSMB)
- Any adverse event which results in the inability to comply with study procedures.
- Ineligibility either arising during the study or retrospectively (having been overlooked during screening).
- Significant protocol deviation.
- Loss to follow up (applies to a subject who consistently does not return for protocol study visits, is not reachable by telephone or any other means of communication and/ is not able to be located).

### 4.6. Managing withdrawals

If a subject is withdrawn for any reason, the reason will be recorded. The reason for withdrawal will be recorded in the clinical report form (CRF). If withdrawal is the result of a serious AE, the investigator will offer to arrange for appropriate specialist management of the problem and the ethical committee will be informed in a timely manner. The extent of follow up will be determined by a medically qualified investigator but will be at least for the whole study period. Subjects withdrawn prematurely for any reason will not receive further vaccinations, although they may be requested to come back to the clinic for safety evaluation.

If a participant withdraws from the study, blood, nasal/oral swab samples collected before his/her withdrawal from the trial will be used/stored unless the participant specifically requests otherwise. In all cases of subject withdrawal, apart from those of complete consent withdrawal, long-term safety data collection for vaccinated participants, including some procedures such as safety blood investigations, will continue so far as the participants are willing to consent. Where participants withdraw consent for follow up, this will be respected and follow up will be discontinued.

If a volunteer withdraws/is withdrawn from the study after receiving PfSPZ Challenge but before reaching the criterion for malaria diagnosis, a complete, appropriate dose of the recommended anti-malarials by the government of Kenya will be provided. The importance of taking this medication will be stressed to the volunteer. They will be encouraged to continue in residence until completion of the treatment to ensure their safety and well-being. In addition, a sample will be taken for COVID-19 testing at withdrawal and if the volunteer is found to be positive referred to RRT for management as per COVID-19 national management guidelines and thus exit residence. The study team will closely follow up on the clinical management outcome of the volunteer under RRT management.

### 4.7. Replacing withdrawn participants

If a participant withdraws/is withdrawn before completing a full vaccination course (3-doses) they will not be replaced.

## 5. TREATMENT OF STUDY PARTICIPANTS

### 5.1. Treatments

The following vaccinations will be given in this study:

- R21 10µg mixed with Matrix-M 50µg
- ChAd63 ME-TRAP 5 x $10^{10}$ vp prime and MVA ME-TRAP 2 x $10^{8}$ pfu boost

### 5.2. Identity of Investigational Product (IP)

***R21/ Matrix M***

R21 is currently being manufactured at Serum Institute of India. The R21 vaccine consists of recombinant HBsAg particles expressing the central repeat and the C-terminus of the circumsporozoite protein from *Plasmodium falciparum* strain NF54. It is 14 amino acids smaller than the RTS fusion protein at the C-terminus of the CSP sequence, and lacks the excess of HBsAg in RTS,S.

Matrix M was manufactured in compliance with cGMP by Apotek Produktion & Laboratorier AB (APL) Formvägen 5B, SE-903 03 Umeå, Sweden. It is supplied as a sterile solution in 3ml glass vials. Matrix M (85 parts Matrix A and 15 parts of Matrix C) is obtained by simply mixing Matrix A and C, followed by dilution in PBS, filtration though filter 0.22 µm and filling into vials in a volume of 2 ml. Matrix M is a colourless slightly-opalescent non-viscous liquid.

The vaccine is presented containing two vials of R21 Antigen at 20 µg/mL each and one vial of Matrix-M Adjuvant at 200 µg/mL. The storage temperature of the vaccine and adjuvant is 2-8 °C. The vaccine mixing will involve withdrawal of 0.5 mL from Vial 1 of R21 and adding it to the Matrix-M vial (containing 0.5ml adjuvant). Another 0.5 ml from Vial 2 of R21 will be added to this mixture. After addition the content will be mixed gently giving a total volume of 1.5 ml mixture. 0.75ml of this mixture will be withdrawn and administered to the volunteers. Each dose of 0.75ml (after mixing of R21 with Matrix-M) will contain 10 µg R21 and 50 µg Matrix M. A mixture of 10µg R21 with Matrix-M 50µg will be administered to volunteers in Groups 1 and 3 and will be administered intramuscularly.

***Viral-vectored vaccines***

ChAd63 ME-TRAP is manufactured under Good Manufacturing Practice (GMP) conditions by the Clinical Biomanufacturing Facility (CBF), University of Oxford. ChAd63 ME-TRAP is supplied as a liquid in sterile aliquots in 2.0 mL clear glass vials. Further details relating to batch release and manufacturing can be found in the ChAd63 ME-TRAP IMP-D.

MVA ME-TRAP is manufactured under Good Manufacturing Practice conditions by IDT Biologika GmbH (IDT), Germany. MVA ME-TRAP is supplied as a liquid formulation in 10mM Tris buffer 140 mM NaCl, pH 7.7. The virus suspension is supplied as sterile aliquots in 2.0 mL clear glass injection vials. Final QP certification and associated labelling takes place at the CBF, University of Oxford. Further details relating to batch release and manufacturing can be found in the MVA ME-TRAP IMP-D.

### 5.3. Storage

***R21***

R21 was previously stored long-term as frozen at -80°C (nominal). R21 is now stored at 2-8 °C.

***Matrix-M***

Matrix-M is stored refrigerated at 2 to 8°C and protected from light.

***Viral-vectored vaccines***

ChAd63 ME-TRAP and MVA ME-TRAP vaccines will be stored frozen at a nominal temperature of -80˚C.

All movements of the vaccines and adjuvants will be documented. Accountability, storage, shipment and handling of vaccines will be in accordance with relevant local SOPs and forms.

### 5.4. Dose Selection

The dosing schedule of R21 is based on the existing doses used in the Phase Ib/IIb trials conducted in Kenya and Burkina Faso which have shown that these doses are safe and immunogenic. The dose of 10µg provides the equivalent amount of malaria protein to RTS,S. Phase IIa trials conducted at the University of Oxford have also shown high level efficacy with this dose. The dose of Matrix M has been chosen based on previous Phase I and II clinical trials, which have administered 50µg of Matrix M as an adjuvant to numerous antigens.

### 5.5. Timing of Doses

Participants in Groups 1 & 3 will receive 3 vaccines, 4 weeks apart. Participants in Group 2 will receive 2 vaccines, 8 weeks apart.

### 5.6. Packaging and Labelling

The vaccine is presented containing two vials of R21 at 20 µg/mL each and one vial of Matrix-M Adjuvant at 200 µg/mL. Each dose of 0.75ml (after mixing of R21 with Matrix-M) will contain 10 µg of R21 and 50 µg of Matrix-M.
ChAd63 ME-TRAP and MVA ME-TRAP are stored between –70$^0$C and –90$^0$C in a locked freezer at the University of Oxford, Churchill Hospital. The vaccines will be shipped from Oxford on dry ice, and then stored in a –70$^0$C freezer.

Labelling will take place at the Clinical Biomanufacturing Facility (CBF), Oxford.

### 5.7. Clinical Reviews of CHMI

All clinical care and procedures will be undertaken by a qualified nurse or clinician trained in the study procedures. Following administration of PfSPZ Challenge, volunteers will remain at Pwani University near to the trial site until the end of CHMI. Clinically qualified staff will be available at all times, and standard operating procedures (SOPs) will be established for out-of-hours cover. Volunteers and staff at the site will have contact numbers of clinically qualified investigators for consultation with a senior clinician at any time during CHMI.

The participants will be monitored by clinical staff with experience of managing clinical *P. falciparum* infection at all times. Resuscitation equipment and anti-malarial drugs will be available at all times. The strain of parasite used for CHMI is known to be sensitive to chloroquine, artemether-lumefantrine (AL), atovaquone/proguanil and sulphadoxine-pyrimethamine (SP). These treatments are all known to be effective for uncomplicated malaria. A full treatment course will be given to all volunteers reaching the end of CHMI.

### 5.8. Safety Measures for CHMI

Volunteer safety is of paramount importance. The following measures are in place to safeguard volunteer safety;

1. All volunteers will be asked to provide details of an emergency contact person who may be contacted if the volunteer cannot be contacted or located following CHMI and before treatment.
2. All doses of artemether-lumefantrine will be observed by the study team. (For volunteers taking other anti-malarials, at least half of all doses will be observed).
3. Volunteers will be counselled to contact the study team for review if they develop fever or other symptoms of malaria in the 6 months following CHMI.

### 5.9. CHMI Malaria Management

All volunteers will be treated with a full course of artemether-lumefantrine. Doses of treatment will be directly observed. The infecting parasites are known to be fully sensitive to artemether-lumefantrine. Volunteers who remain undiagnosed with malaria at Day 21 will start a treatment course of artemether-lumefantrine at this time point. If a patient is unable to tolerate an oral anti-malarial, they will be treated with parenteral Artesunate until they are able to take oral medication. If a volunteer withdraws/is withdrawn from the study after receiving PfSPZ Challenge but before reaching the criterion for malaria diagnosis, a complete, appropriate, curative course of anti-malarial therapy must be completed. The importance of this will be emphasized to volunteers at screening. If a volunteer develops a contraindication to artemether-lumefantrine or is unable to tolerate artemether-lumefantrine, oral chloroquine or SP may be prescribed as an alternative treatment for malaria. The strain of malaria used is known to be sensitive to chloroquine, atovaquone/proguanil, SP, and artemether-lumefantrine. These drugs will be used in accordance with the manufacturer's instructions and the Government of Kenya treatment guidelines.

## 6. ASSESSMENT OF END POINTS

### 6.1. Specification of end point parameters.

#### 6.1.1. Primary outcome measures

***Safety***

The specific endpoints for safety and reactogenicity will be actively and passively collected data on adverse events. The following parameters will be assessed for all study groups

1. Occurrence of solicited local reactogenicity signs and symptoms for 7 days following the vaccination
2. Occurrence of solicited systemic reactogenicity signs and symptoms for 7 days following the vaccination
3. Occurrence of unsolicited adverse events for 28 days following the vaccination
4. Change from baseline for safety laboratory measures
5. Occurrence of serious adverse events during the whole study duration including safety with regards to PfSPZ Challenge administration.

***Efficacy***

Venous blood samples will be taken at each study visit during the post-CHMI follow-up period as described above for quantitative PCR (qPCR) for *P. falciparum* nucleic acids which will be processed within 6 hours of collection. A diagnosis of malaria infection will be made and treatment with anti-malarials commenced immediately for a volunteer satisfying one of the following criteria:

1. The density of infection rises past a threshold of 500 parasites per µl
2. The volunteer develops symptoms or signs of illness and an immediate blood film examination shows any evidence of detectable malaria parasites
3. The volunteer reaches day 21 of monitoring, at which point CHMI will be completed

The investigators are able to treat any volunteer for malaria regardless of the qPCR result if they are clinically concerned (and have discussed the case with the Chief Investigator), or a volunteer wishes to withdraw from the study.

#### 6.1.2. Secondary outcome measures

***Immunology***

**1.** Comparison of immunogenicity (antibody responses) of the R21/MM vaccination doses and the longevity of responses

***Efficacy***

Further measures of vaccine efficacy will be used, as secondary endpoints, to provide more detailed quantitative assessment of the dynamics of malaria infection in sporozoite-challenged volunteers. These measures enable more powerful distinction, than the use of sterile efficacy rates, between the performance of different vaccination regimens, and between individuals receiving the same vaccination regimen, as has been shown for previous efficacy trials. This maximises the yield of information from the volunteers in this study that is used to assess the primary objective.

Kaplan-Meier analyses will be performed for each vaccination regimen and for controls. Separate analyses will be done using various outcome measures for malaria blood stage infection including: (i) parasitaemia as defined by 10,000 or more parasites/ml in peripheral blood by quantitative PCR or composite of symptoms and PCR > 1000 p/ml; (ii) parasitaemia >1,000 p/ml on PCR; (iii) parasitaemia > 500 p/ml on PCR; and (iv) parasitaemia > 20 p/ml. A statistically significant difference between a vaccination regimen and controls by log rank analysis of Kaplan Meier curves any endpoint, will be considered as an indicator of likely vaccine efficacy compared to controls.

As potentially more sensitive but newer measures of vaccine efficacy, the following secondary endpoints will also be assessed. Differences will be calculated between vaccination groups and control groups for parasite density at early post-challenge time-points, using comparisons of mean group parasite densities at cycle peaks, typically 7.5, 9.5 and 11.5 days post challenge, and area under the curve analysis performed of parasite density profiles in the first to third cycles post liver to blood infection Differences between groups will be assessed by T test or non-parametric tests as appropriate for the data distribution.

#### 6.1.3. Tertiary outcome measures

Tertiary immunological measures performed on immunology bloods include:

1. ELISA to quantify antibodies to the vaccine components CS, NANP, TRAP and HBsAb.
2. Flow cytometry assays with intracellular cytokine staining to enumerate and functionally characterise immune cell populations such as effector and memory T cells (e.g. CD4+ and CD8+), T follicular helper cells, regulatory T cells, B cells, plasma cells and dendritic cells

3. Assays to determine antibody function *in vitro*
4. ELISPOT for enumeration of antibody-secreting cells (e.g. B and plasma cells)

Antibody levels and frequencies of cells will be compared by non-parametric tests before and after vaccination.

***Exploratory Immunology may include***

5. Assays to assess immunological aging, dysregulation and senescence, such as telomere length or expression of relevant markers and transcription factors.
6. Assays to assess antibody avidity, sub-types and isotypes
7. Identification and quantification of antibody and B cell repertoire
8. Assays to assess presence or absence of other factors affecting vaccine immunogenicity, such as antibodies against viral pathogens including cytomegalovirus.
9. Other ELISA assays for immunity to malaria that may be relevant to prior malaria exposure and be used to predict vaccine immunogenicity.
10. Genetic tests-determination of HLA-type and associated genes that can have an impact on vaccination. N.B. Specific consent for HLA and genetic testing will be sought through an additional question on the ICF to make clear to participants and parents that consent for genetic testing doesn't not affect participation in the clinical trial.

Aliquots of plasma and PBMC samples will be transferred to Oxford for analysis of humoral and cell-mediated immunity and consent for this will be obtained. This is being done as the assays to analyse these samples have been standardised in Oxford and to ensure comparability between various trials utilising the vaccines under investigation, samples will need to assayed and analysed in a central laboratory at the Jenner Institute, University of Oxford, Oxford, UK. After the end of the study, remaining samples will be transferred back to the Biobank in Kilifi. Long term storage of samples will be undertaken in Kilifi. There will be no-opt of storage and shipment outside of Kenya due to primary object being answered.

The immunoassay of most interest is the antibody response to NANP because this correlates with vaccine efficacy after RTS, S/AS01 administration, and induction of antibody levels comparable to or greater than RTS, S/AS01 would suggest likely vaccine efficacy. In RTS, S trials, IgG titres against the NANP region of CS at D42 (2 weeks after the second vaccination) were most closely correlated with vaccine efficacy, hence the inclusion of an additional immunology blood sample at this time point. This assay has been validated and established in at the Jenner Institute, University of Oxford and therefore will require sample shipment to Oxford, UK.

## 7. ASSESSMENT OF SAFETY

Safety oversight will be the responsibility of the investigators, Local safety monitor (LSM) and the independent Data Safety Monitoring Board (DSMB) that will be convened.

### 7.1. Adverse Events (AEs)

Both solicited and unsolicited AEs will be recorded on the participant's eCRF/paper CRF. The diagnosis, date and time of onset, outcome, severity and relationship to vaccination will be established. Details of any treatment or concomitant interventions will be recorded.

There will be a 7-day follow-up period for solicited AEs post each vaccination visit. Day 0 evaluation will be carried out by the study clinician following enrolment. The volunteers will be provided with rulers and thermometers and trained on how to use these to measure swellings and take temperature the subsequent days following vaccination (days 1 to 7). Additionally, diary cards will be provided for recording the measurements taken. Clinicians will call them

daily to assess their wellbeing and record on a call log and CRF as appropriate. If indicated, the volunteer will be asked to attend a clinicvisit for post-vaccination clinical assessment. If indicated, safety bloods will be taken and documentation of solicited AEs. Severity of adverse events will be the responsibilityof either a clinical or medical officer.

There will be a 30-day (day of vaccination and 29 subsequent days) follow-up after each vaccine dose for reporting unsolicited symptoms.

### 7.2. Definitions and monitoring of AEs

***Adverse Events (AE)***

Any untoward medical occurrence in a patient or clinical investigation subject occurring in any phase of the clinical study whether or not considered related to the vaccine. This includes an exacerbation of pre-existing conditions or events, intercurrent illnesses, or drug interactions. Anticipated day-to-day fluctuations of pre-existing conditions, including the disease under study, that do not represent a clinically significant exacerbation will not be considered AEs. Discrete episodes of chronic conditions occurring during a study period will be reported as adverse events in order to assess changes in frequency or severity.

Unsolicited adverse events will be documented in terms of a medical diagnosis(es). When this is not possible, the AE will be documented in terms of signs and symptoms observed by the investigator or reported by the subject.

Pre-existing conditions or signs and/or symptoms (including any which are not recognised at study entry but are recognised during the study period) present in a subject prior to the start of the study will be recorded on the Medical History form within the subject's CRF.

***Serious/severe Adverse Events (SAE)***

A serious adverse event is any untoward medical occurrence that at any dose:

- results in death
- is life-threatening

  **Note:** The term “life-threatening” in the definition of “serious” refers to an event in which the patient was at risk of death at the time of the event; it does not refer to an event which hypothetically might have caused death if it were more severe.
- requires inpatient hospitalisation or prolongation of existing hospitalisation,
- results in persistent or significant disability/incapacity, or
- results in a congenital anomaly/birth defect

Medical and scientific judgment should be exercised in deciding whether expedited reporting is appropriate in other situations, such as important medical events that may not be immediately life-threatening or result in death or hospitalisation but may jeopardise the patient or may require intervention to prevent one of the other outcomes listed in the definition above. These should also usually be considered serious. SAEs related with drug treatment will not be reported to a sponsor, since the drugs are all licensed, but we will undertake expedited reporting to the local safety monitor and SERU.

***Suspected Unexpected Serious Adverse Reactions (SUSAR)***

An adverse reaction, the nature or severity of which is not anticipated based on the applicable product information is considered as an unexpected adverse drug reaction. Where the adverse reaction is also considered to have a possible, probable or definite relationship with the drugs given, and also meets the criteria for a serious adverse reaction, it is termed a Suspected Unexpected Serious Adverse Reaction (SUSAR). These events are subject to expedited reporting as for SAEs.

***Severity Assessment***

The severity of clinical adverse events relating to vaccination will be assessed according to the scales in Tables 9, 10 and 12. Adverse events relating to malaria infection will be assessed according to Table 11 for symptoms and Table 9 and 10 for severity. Study subjects will be asked to indicate the maximum degree of pain they experience at the injection site following vaccinations using a scale ranging from 0 to 3 as described in Tables 9, 10 and 12. All local reactions will be considered causally related to the vaccination in the absence of another more likely explanation (such as recent trauma).

At each visit, participants will be requested to report local and general side effects they might have experienced since they last were seen. The investigator will assess the severity of the solicited signs and symptoms using the key provided in Tables 9-12. Further details for any AE (such as start/stop date and any treatment), will be gathered, regardless of the relationship to the vaccine. We will also document any unsolicited adverse event reported by the participant/parent/guardian. Serious adverse events (SAE) as defined above will be documented and reported using a serious adverse event reporting form.

**Table 9.** Severity grading criteria for local adverse events

| Adverse Event | Grade | Intensity |
|---|---|---|
| Pain at injection site | 1 | Pain that is easily tolerated |
| | 2 | Pain that interferes with daily activity |
| | 3 | Pain that prevents daily activity |
| Erythema at injection site* | 1 | >3 - ≤50 mm |
| | 2 | >50 - ≤100 mm |
| | 3 | >100 mm |
| Swelling at injection site | 1 | >1 - ≤20 mm |
| | [illegible] | [illegible] |
| | [illegible] | [illegible] |

*Erythema ≤3mm is an expected consequence of skin puncture and will therefore not be considered an adverse event.

**Table 10**. Severity grading criteria for physical observations

| | **Grade 1 (mild)** | **Grade 2 (moderate)** | **Grade 3 (severe)** |
|---|---|---|---|
| Fever | 37.6°C - 38.0°C | 38.1°C – 39.0°C | >39.0°C |
| Tachycardia (bpm)* | 101 - 115 | 116 – 130 | >130 |
| Bradycardia (bpm)** | 50 – 54 | 40 – 49 | <40 |
| Systolic hypertension (mmHg) | 141 - 159 | 160 – 179 | ≥180 |

| | | | |
|---|---|---|---|
| Diastolic hypertension (mmHg) | 91 - 99 | 100 – 109 | ≥110 |
| Systolic hypotension (mmHg)*** | 85 - 89 | 80 – 84 | <80 |

*Taken after ≥10 minutes at rest
**When resting heart rate is between 60 – 100 beats per minute. Use clinical judgement when characterising bradycardia among some healthy subject populations, for example, conditioned athletes.
***Only if symptomatic (e.g. dizzy/ light-headed)

**Table 11.** Solicited adverse events related to malaria infection. Gradings for physical observations abnormalities as per Table 9

| Adverse events | |
|---|---|
| Physical observations | Fever<br>Hypotension<br>Tachycardia |
| Symptoms | Feverishness<br>Chills<br>Rigors<br>Sweating<br>Headache<br>Anorexia<br>Nausea<br>Vomiting<br>Myalgia<br>Arthralgia<br>Low back pain<br>Fatigue |
| Laboratory abnormalities | Lymphopenia<br>Thrombocytopenia |

**Table 12.** Intensity of general adverse events

| | |
|---|---|
| GRADE 0 | None |
| GRADE 1 | Mild: Transient or mild discomfort (< 48 hours); no medical intervention/therapy required |
| GRADE 2 | Moderate: Mild to moderate limitation in activity - some assistance may be needed; no or minimal medical intervention/therapy required |
| GRADE 3 | Severe: Marked limitation in activity, some assistance usually required; medical intervention/therapy required, hospitalization possible |

***Follow-up of Adverse Events***

Adverse events likely to be related to the vaccine, whether serious or not, which persist at the end of the trial will be followed up by the investigator until their resolution or stabilisation, or until causality is determined to be unrelated to trial interventions. All AEs will be managed as per Kenyan national clinical guidelines.

Moreover, any serious adverse event likely to be related to the vaccine and occurring after trial termination should be reported by the investigator according to the procedure described below.

Outcome of any non-serious adverse event occurring within 30 days' post-vaccination (*i.e.* unsolicited adverse event) or any SAE reported during the entire study will be assessed as:

- Recovered/resolved
- Not recovered/not resolved
- Recovering/resolving
- Recovered with sequelae/resolved with sequelae
- Fatal (SAEs only)

Subjects who have moderate or severe on-going adverse events that are not vaccine linked will be referred to an appropriate hospital/health facility on completion of the study and will be advised to consult a primary care physician if the event is not considered to be related to the study vaccine. A follow-up visit will be arranged to manage the problem and to determine the severity and duration of the event, if it is related to the study vaccine. If appropriate, specialist review within the Kilifi County Hospital (KCH) will be arranged.

**Table 13.** Relationship to investigative product

| | | |
|---|---|---|
| 0 | **No Relationship** | No temporal relationship to study product ***and***<br>Alternate aetiology (clinical state, environmental or other interventions); ***and***<br>Does not follow known pattern of response to study product |
| 1 | **Unlikely** | Unlikely temporal relationship to study product ***and***<br>Alternate aetiology likely (clinical state, environmental or other interventions) ***and***<br>Does not follow known typical or plausible pattern of response to study product. |
| 2 | **Possible** | Reasonable temporal relationship to study product; ***or***<br>Event not readily produced by clinical state, environmental or other interventions; ***or***<br>Similar pattern of response to that seen with other vaccines |
| 3 | **Probable** | Reasonable temporal relationship to study product; ***and***<br>Event not readily produced by clinical state, environment, or other interventions ***or***<br>Known pattern of response seen with other vaccines |
| 4 | **Definite** | Reasonable temporal relationship to study product; ***and***<br>Event not readily produced by clinical state, environment, or other interventions; ***and***<br>Known pattern of response seen with other vaccines |

### 7.3. Documenting AEs

Solicited and unsolicited AEs will be recorded on the participant's eCRF/paper CRF. The diagnosis, date and time of onset, outcome, severity and relationship to vaccination will be established. Details of any treatment or concomitant interventions will be recorded.

### 7.4. Reporting Serious Adverse Events (SAEs) and/or Unexpected AEs

Every SAE occurring throughout the trial must be reported by telephone, e-mail or fax to the sponsor (University of Oxford) and DSMB within twenty-four hours, even if the investigator considers the SAE not related to vaccination. The investigator will then complete a SAE report as soon as possible and within 5 working days or 7 calendar days.

Any relevant information concerning the adverse event that becomes available after the SAE report form has been sent (outcome, precise description of medical history, results of the investigation, copy of hospitalisation report, etc.) will be forwarded to the sponsor in a timely manner, the anonymity of the subjects shall be respected when forwarding this information.

The DSMB may ask for the study to be stopped, or for an extended study hold to be applied while further data and information are sought. The DSMB will make its recommendation to the Sponsor, who will have ultimate responsibility for acting on the recommendation.

Any study-related SUSAR or serious adverse event related to participation in the study must be reported by telephone, e-mail or fax to the sponsor (University of Oxford) and DSMB within twenty-four hours, and to the scientific and ethical review committee (SERU) via email within 48 hours of the principal investigator (PI) being aware of the event. The hard copies of the

report must be forwarded to the SERU Secretariat within 5 working days of the initial notification.

If any participants are lost to follow up during CHMI without having completed a course of anti-malarials this will also be reported within 1 working day.

Follow-up reports should be submitted as soon as more information becomes available.Periodic 6 monthly safety reports of any study-related expected adverse event or adverse eventsnot related to participation in the study will be forwarded to SERU.

Initial reports of SUSARs will be provided by the Sponsor to the Pharmacy and Poisons Board (PPB) as soon as possible but within seven (7) calendar days of the notification of the SUSARs with follow up reports being provided within a further eight (8) calendar days.

The SUSAR and SAE reports will be submitted to PPB through the online system at www.pv.pharmacyboardkenya.org. A summary of SAEs and SUSARs shall be submitted every six months from the day of approval of the study. The sponsor pledges to inform the authorities of any trial discontinuation and specify the reason for discontinuation. The causal relationship between the SAE and the product will first be evaluated by the investigator with the following scale (Table 14.)

### 7.5. Study Governance, LSM and DSMB

*Local Safety Monitor*

The Local Safety Monitor (LSM) will be a clinician resident in Kilifi (therefore likely to be linked to or a staff member of KWTRP) but independent of the study team. The LSM will act as a semi-independent assessor of participants experiencing important safety events at the request of the DSMB and will provide their observations to the DSMB and/or local clinicians or study team members where appropriate.

*Data and Safety Monitoring Board*

The DSMB will include at least 3 independent members (at least 1 clinician and 1 statistician), including internationally reputable clinical and/or statistical experts, and will have access to all relevant data on administration of R21/MM and ChAd63-MVA ME-TRAP or related products. The DSMB will include at least 1 member with expert knowledge of the Kenyan context. The DSMB will be convened at the start of the study before vaccinations begin to review the protocol and their responsibilities. A local safety monitor (LSM) will also be appointed at this time to provide independent safety assessments of participants. The DSMB will review:

- all SAEs/SUSARs as they occur
- severe adverse events reported within a week of their occurrence with an updated summary of all severe adverse events reported to date.

We will provide summaries of all adverse events to the sponsor and DSMB. However, investigators or the local safety monitor can request the DSMB to review any non-serious non-severe adverse events that raise concern. The DSMB will consist of the same members as for all the clinical trials funded by the EDCTP grant but there will be an additional DSMB for the CHMI. Information provided to the DSMB for CHMI will be safety data specific to CHMI only.

### 7.6. Emergency Procedures

***Vaccinations***

As with any vaccine, Guillain-Barré syndrome or immune-mediated reactions that can lead to organ damage including serious allergic reactions may occur but this should be extremely rare. Serious allergic reactions including anaphylaxis could also occur and for this reason volunteers will be vaccinated in a clinical area where Basic Life Support trained physicians, equipment and drugs are immediately available for the management of any serious adverse reactions. Any unsolicited adverse events or SAE will be recorded. (See Safety reporting below).

***Administration of PfSPZ***

During administration of PfSPZ Challenge, Basic Life Support drugs and resuscitation equipment will be immediately available for the management of anaphylaxis. Volunteers will be observed for 1 hour after injection before returning to Pwani University or facility where they will be staying. The injection sites will be covered with a sterile dressing. The sterile dressing will be removed no earlier than 1hour post inoculation. Any unsolicited adverse events or SAE will be recorded. (See Safety reporting below).

### 7.7. Pregnancy

Should a volunteer become pregnant during the trial, she will be followed up as other volunteers and in addition will be followed until pregnancy outcome, with the volunteer's permission. We will not routinely perform venipuncture on such volunteers. The management of any volunteer found to be pregnant at any time after infection up to the point of diagnosis with malaria by PCR will be discussed with the LSM. If inadvertent exposure during pregnancy does occur post-vaccination in the trial, then we will undertake to complete follow up to determine the outcome of the pregnancy, and follow up the vaccine-exposed infant for at least 6 months. We will report the event to SERU and other regulatory bodies.

### 7.8. Protocol deviations

A protocol deviation will be any failure to adhere to the defined procedures or treatment plans outlined in the protocol version previously approved by SERU. A protocol violation is any planned or inadvertent changes that may impact safety of study participants, affect integrity of the study data and/or affect study participants willingness to participate in the study previously approved by the SERU. Any unforeseen and unavoidable deviations from the protocol will be documented and filed in a protocol deviation folder, with explanation and reported to the relevant ethical and regulatory authorities as applicable

# 8. STATISTICS

## 8.1. Determination of sample size

This is a Phase IIb study, there will be a minimum of 64 participants. The number of participants being enrolled into the study is contingent on practical issues, and is comparable to previous sporozoite challenge studies done as reported in the literature. Using a comparison of proportions assuming p=0.05 and 100% infection rates in a control arm there is 90% power to detect 40% efficacy in the n=20 groups, and 80% power to detect 50% efficacy in the control group n=14, and 80% power to detect 60% efficacy in the n=10 group.

## 8.2. Statistical and analytical plans

### 8.2.1.1. Statistical analysis

The null hypothesis is that there is no difference in the rate of *P. falciparum* infection (as defined by PCR monitoring post CHMI) between vaccination groups.

***Intent-to-treat cohort (ITT)***

The intent-to-treat cohort will include all subject volunteers in the study, who received at least one dose of the candidate malaria vaccines or comparator and for whom data for the observation in question are available.

***Protocol-defined cohort (ATP)***

The protocol-defined cohort for analysis of efficacy and immunogenicity will include all evaluable volunteers (i.e., those meeting all eligibility criteria, complying with the procedures defined in the protocol), for whom data concerning efficacy endpoint measures are available.

***Baseline data.***

Age, location of residence, PCR at day 0 and bed net use will be presented by group to ensure comparability.

**Safety Review**

No interim safety analysis is planned, but real-time reporting of SAEs and SUSARs may lead to interim safety analyses if requested by the DSMB. Safety analysis will be done for the ITT cohort. All reactogenicity analyses will be on an intention to treat basis, comparing control to malaria vaccine regimen. The frequency of adverse events, changes for example in ALT, plasma creatinine, haemoglobin, white cell count, platelet values etc. will be described by time and vaccine group.

***Immunogenicity***

ATP cohorts will be analysed, using ELISA, immediate *ex vivo* and cultured ELISpots, and ICS data. Groups will be described using geometric means and medians of T cell numbers and antibody levels with 95% confidence intervals. Comparisons of immunogenicity will not be formally adjusted for multiple comparisons (e.g. using a Bonferroni adjustment) but the multiple comparisons made will be considered in the interpretation of significance levels.

***Efficacy***

Efficacy will be analysed both ITT and ATP. Log-likelihood tests of survival functions will be conducted on PCR positivity meeting treatment thresholds. PCR positivity will be defined by threshold, using >100 parasites per ul as the primary endpoint, but including other thresholds for comparison. Age, bed net use and location of residence will be used as covariates to adjust.

Immunological responses will also be examined as covariates to look for correlates of immunity.

The full analysis plan will be approved by the DSMB before implementation.

#### 8.2.1.2. Stopping criteria for termination of trial

The study will be placed on safety hold if:

- One or more participants experience any SAE related to the study product (possible, probable or related)
- A suspected unexpected serious adverse drug reaction (SUSAR) occurs.

## 9. DIRECT ACCESS TO SOURCE DATA/DOCUMENTS

The PI will provide direct access to the source data documents to the ethics committee should this be requested, to the regulatory agency, and to authorised representatives of the sponsor in order to permit trial related monitoring and audits.

## 10. QUALITY CONTROL AND QUALITY ASSURANCE

The QA manager at the trial site will conduct internal audits if indicated to check that the trial is being conducted, data is being recorded, analysed and accurately reported according to the protocol, trial SOPs, and in compliance with ICH GCP. The audits will also include laboratory activities according to an agreed audit schedule. The Clinical Trials and Research Governance Office in Oxford may carry out an audit to ensure compliance with the protocol, GCP and appropriate regulations.

## 11. INTELLECTUAL PROPERTY

Any intellectual property rights that arise from the work will be safeguarded according to the current KEMRI guidelines and the Industrial Property Act of 2001, sections 32, 58 and 80. The scientific and intellectual contributions of all persons involved in the research will be appropriately acknowledged in all publications and presentations arising from the work.

## 12. TIME FRAME/DURATION OF THE TRIAL

| **Activity** | **Q1 2022** | **Q2 2022** | **Q3 2022** | **Q4 2022** | **Q1-Q4 2023** |
|---|---|---|---|---|---|
| Community engagement and mobilisation | ■ | ■ | | | |
| Recruitment and screening | ■ | ■ | ■ | | |
| Vaccinations | ■ | ■ | ■ | ■ | |
| CHMI | | ■ | ■ | ■ | ■ |
| CHMI – follow up | | ■ | ■ | ■ | ■ |
| Sample Collection | ■ | ■ | ■ | ■ | ■ |
| Laboratory analysis | ■ | ■ | ■ | ■ | ■ |
| Completion of data collection | | | | | ■ |
| Data analysis | | | ■ | ■ | ■ |
| Write Up | | | | ■ | ■ |
| Feedback to study participants | | | | | ■ |

## 13. ETHICS

Before any study procedures can begin the protocol must be approved by SERU.

### 13.1. Human Subjects

"First, do no harm."

We take seriously the potential risks involved in CHMI and have undertaken a smaller pilot study and will have a DSMB in place. However, to contextualize the risks 30 to 60% of the adult population in Kilifi have falciparum parasites detectable in their blood by PCR and 8 to 20% are positive by microscopy. These infections persist for an average of 9 months and clinical illness is rare and severe malaria extremely rare (and difficult to detect in surveillance of adult illness via the hospital in Kilifi). Malaria is a serious disease and can lead to death if left untreated. Most people do not develop symptoms but have the parasites in their blood, whilst others only develop a mild form of the disease, and others may become very seriously sick and may die if they are not treated. Therefore, monitoring will be done throughout the timefrom challenge to ensure that they are treated, and the infection is cleared before they are released from the study. In consideration of this, participants will be accommodated at a guesthouse at Pwani University (which is near KEMRI-CGMRC) at least seven days before infectionto enable monitoring of malaria infection. At Pwani University, the participants will bemonitored throughout the malaria infection and treatment. Each participant will have access toinsecticide treated bednets to prevent any further infection with any other malaria frommosquitoes and will be given.

Furthermore, even in non-immune populations CHMI appears to be very safe [51]. More than 1000 volunteers have taken part in CHMI studies that have been conducted and myocardial events have occurred in five volunteers in the Netherlands following mosquito bite challenge, one with prior cardiovascular risk factors and evidence of atherosclerotic infarction, and the others without risk factors and a less clear clinical picture [40, 41] (unpublished findings). These five isolated events were not definitively linked to CHMI and are not widely recognized consequences of natural malaria infection. All occurred in the Netherlands and no such events have occurred in the CHMI studies conducted elsewhere, including CHMI studies in Tanzania, Kenya and Gabon. Furthermore, no participant in CHMI has thus far developed an illness meeting criteria for severe malaria.

Ethical Review

Before the inclusion of the first subject in the study, the protocol and the informed consent must be approved by SERU and Oxford Tropical Research Ethics Committee (OxTREC) and the Pharmacy and Poisons Board (PPB).

The participant will give written informed consent before being included in the trial, after having been informed of the nature of the trial, the potential risks and their obligations. Informed consent forms will be provided in duplicate (original kept by the investigator, one copy kept by the subject or the subject's legally acceptable representative).

Confidentiality

Personal information of the participants will be handled confidentially. All HIV tests and HIV related referrals will be handled with sensitivity. They will be linked to personal identifying information only on documents held securely in the Clinical Trials Facility, and no personal

information will be entered into the electronic database. This will identify subjects by their unique code number only. Immunology bloods will not have personal identifying information. Any future research related to the data or samples from this study will require written approval from SERU as well as the UK Biobank committee before it can be done.

### 13.2. Community Engagement and Sensitisation

A detailed plan of community engagement activities will be drawn up to include sensitization about the study to several stakeholders and potential participants in accordance with national COVID-19 compliance measures.. CGMRC will use existing community engagement strategies to inform communities about the study. Meetings with institutional heads, chiefs, community leaders and community representatives will be organised to explain the study and its aims, and to discuss concerns. Subsequently further meetings will be organized with potential participants where investigators will present the study including the risks and benefits of participation and describe the inconvenience and procedures required for participation in detail.

A community engagement plan specific for the study will be developed between the Community Liaison Group (CLG) team and the investigators. Community barazas in Ngerenya, meetings with chiefs, sub-chiefs, community representatives, and the Department of Health, Kilifi County will be held to inform them about the study. In addition, there will be engagement meetings at Pwani University. Community engagement will continue throughout the study period collecting and responding to concerns from the community about the study through the CLG team.

### 13.3. Informed Consent

Informed consent will be obtained by trained members of the study team such as clinicians, nurses and fieldworkers. All participants will sign and date the informed consent form before any study specific procedures are performed. The clinician will have a checklist for them to discuss with the potential participant aimed to assess their understanding of the study. Participants will have ample time to read the patient information sheet. All informed consent documents will be translated into Kiswahili and Giryama. We will emphasize that:

- Participation in the study is entirely voluntary.
- Declining to participate involves no penalty or loss of medical benefits.
- A participant may withdraw from the study at any time however this not desirable to ensure participant safety and well-being.
- A participant is free to ask questions at any time to allow him or her to understand the purpose of the study and the procedures involved.
- There is no direct benefit from participating. The benefits will be realized in the long-term for the community by contributing towards the development of a malaria vaccine.

### 13.4. Compensation

Compensation for study related out of pocket expenses are informed by an existing guideline on payment and benefits within KEMRI-CGMRC. We will compensate participants for study related out of pocket costs, this includes transport, for each clinic study visit. Participants who do not have adequate methods of contraception will be referred to their local dispensary where this will be dispensed for free or to Kilifi County Hospital. Costs incurred for obtaining contraception at KCH will be covered by the study. Rates are based on Kenyan government minimum wage guidelines (currently 350/= per clinic visit), and we will reimburse transport costs incurred (depending on distance from the clinic, ranging from 100/= return to 1,200/= per visit). During residence at Pwani University, compensation for out of pocket expenses will be

at a rate of Ksh2, 000 per overnight stay. The amounts will be paid on a weekly basis via mobile money transfer or bank account. Based on the amounts that need to be paid for each participant enrolled into the study, an amount of money (30% of the total out of pocket expense) will need to be paid to Kenya Revenue Authority (KRA). For this to be possible, each participant will need to provide a KRA personal identification number (PIN) and in case participants do not have this, one would need to be obtained. The study will cover the cost of only one ATM or mobile money withdrawal which will be transferred together with the total cost of reimbursement. The study will also cover the cost of the KRA tax amount. The payment of TAX by the participants is a requirement specified by KRA due to the amount of compensation being received.

### 13.5. Patient Data Protection/Confidentiality

The clinical records will be kept in locked designated cabinets of the clinical trials facility. All immunological and PCR data will be kept in anonymized databases linked by the study number to clinical data. The data will be stored in password protected computers and the hard copy documents will be stored in lockable cabinets. Participant identifiable information will not be shared in any way that is not necessary for the day-to-day administration of the trial. Participant identifiable information will not be published. History taking and examination for participants will be carried out with the normal respect towards privacy, dignity and confidentiality. Clinical written information arising from such episodes will be held securely, as normal patient confidentiality guidelines dictate.

### 13.6. Data and Sample Sharing

The study is occurring in collaboration with the Jenner Institute. Individual-level anonymized data will be shared with the sponsor. For wider stake-holder engagement and the medical community, summary-level statistical analyses will be shared. Information collected or generated during this study will be de-indentified for use to support new research on malaria vaccines. Long term storage of samples (15 years) will be undertaken at KEMRI-CGMRC in Kilifi with only aliquots of samples being shared with collaborators. Any future research using information and samples from this study must first be approved by a local or national expert committee to make sure that the interests of participants and their communities are protected. Data will be managed by KWTRP.

## 14. ARCHIVING AND RECORD RETENTION

### 14.1. Data Management

The PI will have overall responsibility for ensuring management of the data. A designee to the PI will be responsible for receiving, entering, cleaning, querying, analysing and storing all data that accrues from the study. Responsibility for this may be delegated to the to the study data management team. The data will be entered into the subjects' paper CRFs. Data will be subsequently transferred to an electronic database for analysis.

If any changes to the study are necessary during the study a formal amendment will be presented to the sponsor prior to submission to the relevant ethical and regulatory agencies for approval unless to eliminate an immediate hazard(s) to study participant without prior ethics approval. Any unforeseen and unavoidable deviations from the protocol will be documented and filed in as a protocol deviation in the Trial Master File, with explanation.

### 14.2. Data capture methods

Data capture will be primarily via an offline database for the scheduled visits through use of encrypted computers, laptops and PDAs/smart phones. These data will then be transferred to an eCRF. Paper source documents will be used to capture data for the screening visits, unscheduled clinic visits, lab and other investigational results. Data on scheduled visits may still be captured on paper source documents if electronic methods fail in the field. Immunological and PCR data will be transferred to an electronic database for analysis without any volunteer identifier apart from the unique volunteer number.

### 14.3. Archiving

The investigator must keep the consent forms and trial master file for at least 10 years after the completion or discontinuation of the trial. The anonymized electronic databases will be maintained beyond this period.

## 15. FINANCING AND INSURANCE

### 15.1. Budget

| | USD $ | Ksh |
|---|---|---|
| a) Personnel, salaries and benefits disbursements | 104,574.00 | 10,561,974.00 |
| b) Patient costs, travel, food, accommodation and/or supplies | 132,060.00 | 13,338,000.00 |
| c) Equipment | 2,940 | 297,000 |
| d) Supplies | | |
| Laboratory consumables: | 222,718.00 | 22,494,600.00 |
| Clinical consumables: | 4,278.00 | 432,000.00 |
| e) Travel and accommodation | | |
| local | 7,750 | 782,000 |
| international | 39,110 | 3,950,000 |
| f) Transportation, vehicle repairs etc | 46,540 | 4,700,000 |
| g) Operating expenses postage, printing etc. | 14,850 | 1,500,000 |
| h) Contingency fees (15% of above) | 86,222.00 | 8,708,400 |
| Total | 661,042.00 | 66,763,974.00 |

### 15.2. Justification of the Budget

This work has been funded by the European Development for Clinical Trials Partnership (EDCTP). This study is part of a larger body of work funded by EDCTP with KEMRI-Wellcome Trust Research Programme as a collaborator and thus will not incur any consultancy fees or additional administrative overheads.

Costs for patients and supplies (equipment, laboratory and clinical consumables) are estimates based on expenses incurred by a similar project recruiting the same number of participants at KEMRI-CGMRC. The costs for participant disbursements are based on the existing guideline on payment and benefits within KEMRI-CGMRC. The costs for travel and accommodation are based on estimates of travel within Kilifi County (local) and on the possible number of visits to collaborating sites and travel for conferences to present the research findings (international). Vehicle and other transportation costs are based on costs provided by the transport department

at KEMRI-CGMRC. Operating expenses are based on costs provided by the purchasing and supplies department at KEMRI-CGMRC.
This study will provide a platform for further studies to be conducted using the samples to be stored. Additional ethics approval will be obtained for sub-analyses if different from primary, secondary, and tertiary objectives. It is envisaged that the additional sub-studies will provide training for potential PhD students and entry-career post-doctoral candidates.

### 15.3. Insurance

The University of Oxford has a specialist insurance policy in place which would operate in the event of any participant suffering harm because of their involvement in the research. Sanaria Inc. has product insurance for non-negligent harm arising because of exposure to PfSPZ Challenge. Local insurance will also be taken for participants enrolled in the study.

## 16. TRIAL MANAGEMENT

The trial will be registered by University of Oxford through the Clinical Trials and Research Governance team at University of Oxford on the clinical trial registry before participant enrollment into the study. A DSMB will be convened on behalf of the sponsor and will consist of 5 individuals who cover clinical, statistical expertise including at least 2 DSMB members based in Kenya. The DSMB will receive reports of all SAEs and SUSARs as well as volunteers lost to follow up during CHMI. The DSMB will be empowered to stop some or all trial procedures by recommendation to the Sponsor, PI. If such a recommendation is made, then SERU will be informed within 3 working days of the recommendation. The DSMB Charter will be drawn before participant enrollment. The investigators will be responsible for reportinga summary of safety data at the end of the trial to the Kenya Pharmacy and Poisons Board and will report SUSARs and SAEs deemed causally related to the study vaccine to the Pharmacy and Poisons Board as stated above (see section 10).

The University of Oxford will sponsor the study and provide insurance for the trial. Regular monitoring will be performed according to International Council for Harmonisation (ICH) Good Clinical Practice (GCP) and a Monitoring Plan. Monitors will check whether the clinical trial is conducted, and data are generated, documented and reported in compliance with the protocol, GCP and the applicable regulatory requirements. The site team led by the PI will be responsible for local submissions to the regulators and all the staff will have good clinical practice training prior to study start.

## 17. REPORTING, DISSEMINATION AND NOTIFICATION OF RESULTS

Results will be published in an open-access journal. Anonymized data on PCR values and immunological data will be made available with these publications. We will feedback individual results with clinical relevance to participants in real-time. Summaries of the outcomes of the trial will be provided during community meetings in the areas from which participants are recruited. It is not anticipated that substantial information in this form will be available until at least the second year of the trial, and this will be made clear during initial meetings to avoid unrealistic expectations regarding the rapidly of feedback.

## 18. REFERENCES

1. WHO. World Malaria Report. 2016.

2. Gething PW, Casey DC, Weiss DJ, Bisanzio D, Bhatt S, Cameron E, et al. Mapping Plasmodium falciparum Mortality in Africa between 1990 and 2015. The New England journal of medicine. 2016;375(25):2435-45.

3. Ashley EA, Dhorda M, Fairhurst RM, Amaratunga C, Lim P, Suon S, et al. Spread of artemisinin resistance in Plasmodium falciparum malaria. The New England journal of medicine. 2014;371(5):411-23.

4. Dondorp AM, Nosten F, Yi P, Das D, Phyo AP, Tarning J, et al. Artemisinin resistance in Plasmodium falciparum malaria. The New England journal of medicine. 2009;361(5):455-67.

5. Sutherland CJ, Lansdell P, Sanders M, Muwanguzi J, van Schalkwyk DA, Kaur H, et al. pfk13-Independent Treatment Failure in Four Imported Cases of Plasmodium falciparum Malaria Treated with Artemether-Lumefantrine in the United Kingdom. Antimicrobial agents and chemotherapy. 2017;61(3).

6. Maxmen A. Malaria surge feared. Nature. 2012;485(7398):293.

7. Group MVF. Malaria VaccineTechnology Roadmap. 2013.

8. Efficacy and safety of RTS,S/AS01 malaria vaccine with or without a booster dose in infants and children in Africa: final results of a phase 3, individually randomised, controlled trial. Lancet (London, England). 2015;386(9988):31-45.

9. Machingaidze S, Wiysonge CS, Hussey GD. Strengthening the expanded programme on immunization in Africa: looking beyond 2015. PLoS medicine. 2013;10(3):e1001405.

10. Olotu A, Lusingu J, Leach A, Lievens M, Vekemans J, Msham S, et al. Efficacy of RTS,S/AS01E malaria vaccine and exploratory analysis on anti-circumsporozoite antibody titres and protection in children aged 5-17 months in Kenya and Tanzania: a randomised controlled trial. The Lancet infectious diseases. 2011;11(2):102-9.

11. Agnandji ST, Lell B, Soulanoudjingar SS, Fernandes JF, Abossolo BP, Conzelmann C, et al. First results of phase 3 trial of RTS,S/AS01 malaria vaccine in African children. The New England journal of medicine. 2011;365(20):1863-75.

12. Moorthy VS, Pinder M, Reece WHH, Watkins K, Atabani S, Hannan C, et al. Safety and immunogenicity of DNA/modified vaccinia virus ankara malaria vaccination in African adults. The Journal of infectious diseases. 2003;188(14551895):1239-44.

13. McConkey SJ, Reece WH, Moorthy VS, Webster D, Dunachie S, Butcher G, et al. Enhanced T-cell immunogenicity of plasmid DNA vaccines boosted by recombinant modified vaccinia virus Ankara in humans. Nature medicine. 2003;9(6):729-35.

14. Lalvani A, Aidoo M, Allsopp CE, Plebanski M, Whittle HC, Hill AV. An HLA-based approach to the design of a CTL-inducing vaccine against Plasmodium falciparum. Research in immunology. 1994;145(6):461-8.

15. O'Hara GA, Duncan CJ, Ewer KJ, Collins KA, Elias SC, Halstead FD, et al. Clinical assessment of a recombinant simian adenovirus ChAd63: a potent new vaccine vector. J Infect Dis. 2012;205(5):772-81.

16. Reyes-Sandoval A, Wyllie DH, Bauza K, Milicic A, Forbes EK, Rollier CS, et al. CD8+ T effector memory cells protect against liver-stage malaria. J Immunol. 2011;187(3):1347-57.

17. Dunachie SJ, Walther M, Epstein JE, Keating S, Berthoud T, Andrews L, et al. A DNA prime-modified vaccinia virus ankara boost vaccine encoding thrombospondin-related

adhesion protein but not circumsporozoite protein partially protects healthy malaria-naive adults against Plasmodium falciparum sporozoite challenge. Infection and immunity. 2006;74(10):5933-42.

18. Webster DP, Dunachie S, Vuola JM, Berthoud T, Keating S, Laidlaw SM, et al. Enhanced T cell-mediated protection against malaria in human challenges by using the recombinant poxviruses FP9 and modified vaccinia virus Ankara. Proceedings of the National Academy of Sciences of the United States of America. 2005;102(13):4836-41.

19. Ogwang C, Kimani D, Edwards NJ, Roberts R, Mwacharo J, Bowyer G, et al. Prime-boost vaccination with chimpanzee adenovirus and modified vaccinia Ankara encoding TRAP provides partial protection against Plasmodium falciparum infection in Kenyan adults. Science translational medicine. 2015;7(286):286re5.

20. Cox RJ, Pedersen G, Madhun AS, Svindland S, Saevik M, Breakwell L, et al. Evaluation of a virosomal H5N1 vaccine formulated with Matrix M adjuvant in a phase I clinical trial. Vaccine. 2011;29(45):8049-59.

21. Roestenberg M, Bijker EM, Sim BK, Billingsley PF, James ER, Bastiaens GJ, et al. Controlled human malaria infections by intradermal injection of cryopreserved Plasmodium falciparum sporozoites. The American journal of tropical medicine and hygiene. 2013;88(1):5-13.

22. Sheehy SH, Douglas AD, Draper SJ. Challenges of assessing the clinical efficacy of asexual blood-stage Plasmodium falciparum malaria vaccines. Human vaccines & immunotherapeutics. 2013;9(9):1831-40.

23. Shekalaghe S, Rutaihwa M, Billingsley PF, Chemba M, Daubenberger CA, James ER, et al. Controlled human malaria infection of Tanzanians by intradermal injection of aseptic, purified, cryopreserved Plasmodium falciparum sporozoites. The American journal of tropical medicine and hygiene. 2014;91(3):471-80.

24. Gomez-Perez GP, Legarda A, Munoz J, Sim BK, Ballester MR, Dobano C, et al. Controlled human malaria infection by intramuscular and direct venous inoculation of cryopreserved Plasmodium falciparum sporozoites in malaria-naive volunteers: effect of injection volume and dose on infectivity rates. Malar J. 2015;14:306.

25. Mordmuller B, Supan C, Sim KL, Gomez-Perez GP, Ospina Salazar CL, Held J, et al. Direct venous inoculation of Plasmodium falciparum sporozoites for controlled human malaria infection: a dose-finding trial in two centres. Malar J. 2015;14:117.

26. Hodgson SH, Juma E, Salim A, Magiri C, Kimani D, Njenga D, et al. Evaluating controlled human malaria infection in Kenyan adults with varying degrees of prior exposure to Plasmodium falciparum using sporozoites administered by intramuscular injection. Frontiers in microbiology. 2014;5:686.

27. Douglas AD, Edwards NJ, Duncan CJ, Thompson FM, Sheehy SH, O'Hara GA, et al. Comparison of modeling methods to determine liver-to-blood inocula and parasite multiplication rates during controlled human malaria infection. J Infect Dis. 2013;208(2):340-5.

28. Douglas AD, Andrews L, Draper SJ, Bojang K, Milligan P, Gilbert SC, et al. Substantially reduced pre-patent parasite multiplication rates are associated with naturally acquired immunity to Plasmodium falciparum. J Infect Dis. 2011;203(9):1337-40.

29. Sheehy SH, Spencer AJ, Douglas AD, Sim BK, Longley RJ, Edwards NJ, et al. Optimising Controlled Human Malaria Infection Studies Using Cryopreserved P. falciparum Parasites Administered by Needle and Syringe. PloS one. 2013;8(6):e65960.

30. Nieman AE, de Mast Q, Roestenberg M, Wiersma J, Pop G, Stalenhoef A, et al. Cardiac complication after experimental human malaria infection: a case report. Malar J. 2009;8:277.

31. van Meer MP, Bastiaens GJ, Boulaksil M, de Mast Q, Gunasekera A, Hoffman SL, et al. Idiopathic acute myocarditis during treatment for controlled human malaria infection: a case report. Malar J. 2014;13:38.

32. Sauerwein RW, Roestenberg M, Moorthy VS. Experimental human challenge infections can accelerate clinical malaria vaccine development. Nat Rev Immunol. 2011;11(1):57-64.

33. Lees R. TREATMENT OF GENERAL PARALYSIS OF THE INSANE BY INDUCED MALARIA: NOTE ON FIFTY CASES. Br Med J. 1931;2(3685):336-9.

34. Trager W, Jensen JB. Human malaria parasites in continuous culture. Science. 1976;193(4254):673-5.

35. Ifediba T, Vanderberg JP. Complete in vitro maturation of Plasmodium falciparum gametocytes. Nature. 1981;294(5839):364-6.

36. Campbell CC, Collins WE, Nguyen-Dinh P, Barber A, Broderson JR. Plasmodium falciparum gametocytes from culture in vitro develop to sporozoites that are infectious to primates. Science. 1982;217(4564):1048-50.

37. Chulay JD, Schneider I, Cosgriff TM, Hoffman SL, Ballou WR, Quakyi IA, et al. Malaria transmitted to humans by mosquitoes infected from cultured Plasmodium falciparum. The American journal of tropical medicine and hygiene. 1986;35(1):66-8.

38. Herrington DA, Clyde DF, Losonsky G, Cortesia M, Murphy JR, Davis J, et al. Safety and immunogenicity in man of a synthetic peptide malaria vaccine against Plasmodium falciparum sporozoites. Nature. 1987;328(6127):257-9.

39. Ballou WR, Hoffman SL, Sherwood JA, Hollingdale MR, Neva FA, Hockmeyer WT, et al. Safety and efficacy of a recombinant DNA Plasmodium falciparum sporozoite vaccine. Lancet. 1987;1(8545):1277-81.

40. Moorthy V. Standardization of Design and Conduct of P. falciparum Sporozoite Challenge Trials. 2011.

41. Howie SR. Blood sample volumes in child health research: review of safe limits. 2010 10th September 2010.

# 19. APPENDICES

## 19.1. Roles of Investigators

| | MK | MH | PB | JM | ON | FO | MD | AL | RR | KE | AH |
|---|---|---|---|---|---|---|---|---|---|---|---|
| Study design | ■ | ■ | ■ | ■ | ■ | ■ | ■ | ■ | ■ | ■ | ■ |
| Clinical | | ■ | ■ | ■ | ■ | | | | | | |
| Laboratory | ■ | | | | | ■ | | | | ■ | |
| Supervision | ■ | ■ | ■ | | | ■ | | | | ■ | ■ |
| Study analysis | ■ | ■ | ■ | ■ | ■ | ■ | ■ | ■ | ■ | ■ | ■ |
| Manuscript write up | ■ | ■ | ■ | ■ | ■ | ■ | ■ | ■ | ■ | ■ | ■ |

MK: Melissa Kapulu BSc MSc DPhil; MH: Mainga Hamaluba MBBS PhD; PB: Philip BejonMBBS PhD; JM: Janet Musembi CO (clinical officer); OM: Omar Ngoto CO (clinical officer);FO: Faith Osier MBChB PhD; MD: Mehreen Datoo MBBS; AL: Alison Lawrie BSc PhD; RR:Rachel Roberts BSc MSc; KE: Katie Ewer BSc PhD; and AH: Adrian Hill MBBS DPhil

## 19.2. CVs of non-KEMRI investigators

## 19.3. Ethics Certificates of all investigators

## 19.4. Informed Consent documents

## 19.5. COVID-19 Mitigation Plan

## 19.6. COVID-19 Risk Information documents

KEMRI | Wellcome Trust

# Safety, immunogenicity, and efficacy of R21 / Matrix-M and ChAd63/MVA-ME-TRAP in the context of controlled human malaria infection: A phase IIb Trial in Kenyan Adults.

## Statistical Analysis Plan (SAP)

### ADMINISTRATIVE INFORMATION

This document has been written based on information contained in the study protocol Version 1.5 6April2022.

| | |
|---|---|
| Protocol Number | KEMRI/SERU/CGMR-C/158/3844 |
| Trial registration Number | NCT03947190 |
| Study Code | VAC074 |
| SAP Version Number | 1.1 |
| Date | 2nd December 2022 |

**Roles and Responsibility – Signatures**

| | NAME | ROLE | SIGNATURE | DATE |
|---|---|---|---|---|
| Written by: | Hillary K. Kiprono | Data Manager | | 2nd December 2022 |
| Reviewed by: | Philip Bejon | Statistician | | |
| Quality Authority by: | Lilian Mwango | Quality Assurance | | |
| Approved by: | Melissa Kapulu | Principal Investigator | | |

**SAP Revision History**

**Version 1.0: Summary of changes**

| Protocol version | Updated SAP version no. | Section number changed | Description of and reason for change | Date changed |
|---|---|---|---|---|
| 1.0 | 1.0 | NA | NA - Initial version | 3rd July 2019 |
| 1.5 | 1.1 | | Standardize the plan to include components such as statistical considerations and planned analysis. | 2nd December 2022 |
| | | | | |

## ABBREVIATIONS

| | |
|---|---|
| ATP: | Protocol defined cohort. |
| CHMI: | Controlled Human Malaria Infection |
| DVI: | Direct Venous inoculation |
| DSMB: | Data Safety Monitoring Board |
| ID: | Intradermal |
| ITT: | Intent-to-treat |
| HR: | Hazard Rate |
| PCR: | polymerase chain reaction |
| PfSPZ: | *P. falciparum* sporozoite challenge |
| SAP: | Statistical Analysis Plan. |
| SAE: | Serious Adverse Event |
| SUSAR: | Suspected Unexpected Serious Adverse Event |

## Table of Contents

Administrative Information ........ 1
Abbreviations ........ 3
1. Introduction ........ 6
2. Purpose and the scope of the plan. ........ 7
3. Hypothesis, objectives and endpoints ........ 7
3.1. Hypothesis ........ 7
3.2. Objectives. ........ 7
3.2.1. Primary Objectives ........ 7
3.2.2. Secondary Objective ........ 9
3.2.3. Tertiary objective. ........ 10
4. Study Design Overview ........ 10
4.1. Overview ........ 10
4.2. Target Population ........ 12
5. Analysis sets. ........ 12
5.1. Intent-to-Treat (ITT) Analysis Set. ........ 12
5.2. Protocol-defined cohort (ATP) ........ 12
5.3. Groups ........ 13
6. Statistical considerations. ........ 13
6.1. General Principles. ........ 13
6.2. Sample size. ........ 13
6.3. Randomization and blinding. ........ 13
6.4. Statistical interim analysis. ........ 14
6.5. Timing of the final analysis. ........ 14
6.6. Timing of the outcome assessment. ........ 14
6.7. Handling of dropouts and Missing Data. ........ 14
6.8. Adherence and protocol deviations. ........ 14
7. Planned Analysis. ........ 14
7.1. Flow of study design (consort diagram). ........ 14
7.2. Demographic and Baseline characteristics. ........ 14
7.3. Safety Analysis. ........ 15

7.4. Efficacy ........ 15
7.5. Immunogenicity ........ 15
7.6. Immune response and risk on malaria infection ........ 16
8. Validation ........ 16
9. Statistical tables to be generated. ........ 16
9.1. Baseline characteristics. ........ 16
9.2. Safety Analysis. ........ 16
9.3. Efficacy ........ 17
10. Statistical listing to be generated. ........ 17
11. Statistical graphs to be generated ........ 17
12. Template Tables ........ 18
12.1. Solicited Adverse Events ........ 18
12.2. Serious Adverse Events ........ 19
12.3. Laboratory Safety Assessments ........ 19
12.4. Immunogenicity ........ 19
12.5. Efficacy ........ 20

# 1. INTRODUCTION

This Statistical and Reporting Analysis Plan (SAP) describes the descriptive and inferential statistical analyses of the efficacy, safety and immunological data collected under VAC 074. It does not cover tertiary endpoints and analysis.

We plan to evaluate the immunogenicity and efficacy of candidate malaria vaccines incorporating the sporozoite antigen R21 (*P. falciparum* circumsporozoite protein co-expressed with hepatitis B antigen) administered as protein-in-adjuvant with the Matrix M and viral-vectored vaccines expressing the liver antigen TRAP (*P. falciparum* multiple epitope thrombospondin adhesion protein, ME-TRAP expressed in Chimpanzee Adenovirus 63 and Modified Vaccinia Ankara administered in a heterologous prime-boost regimen). There is evidence of safety and immunogenicity utilising these two vaccines. We plan to conduct a phase IIb trial in malaria-exposed individuals to assess the immunogenicity and efficacy of the two vaccines in the context of controlled human malaria infection. *P. falciparum* sporozoite challenge, PfSPZ Challenge.

Healthy adults aged between 18-45 years will be recruited to participate in the study after a process of information giving and sensitisation about the study. Those that provide informed consent to participate in the study will be screened to ensure they are in good health based on clinical assessment and laboratory results. Each participant will have blood tests undertaken, and physical and clinical examination to ensure suitability prior to vaccination and PfSPZ Challenge. There will be follow-up after exit from residence for up to 90 days after PfSPZ Challenge.

A total of up to 80 volunteers will be enrolled for challenge and divided into four groups as follows: 24 participants to receive R21/Matrix M (R21/MM) with intradermal PfSPZ Challenge; 24 participants to receive viral-vectored ME-TRAP with intradermal PfSPZ Challenge; 14 participants to receive R21/MM with direct venous inoculation PfSPZ Challenge; and 18 participants comprising of the control group with intradermal PfSPZ Challenge. Blood tests and clinical assessments will be conducted to screen out participants with health conditions that may impact participation in the study.

Blood will also be taken prior to each vaccination as well as after PfSPZ Challenge administration until diagnosis with malaria. A set threshold for malaria diagnosis will be met and once this is achieved, participants will be treated with the recommended anti-malaria drug treatment. Blood samples to assess immunogenicity will be taken prior to vaccination, and throughout the study to assess vaccine-induced immune responses. Efficacy will be assessed in relation to the control group with serial quantitative polymerase chain reaction (PCR) monitoring of parasitaemia during PfSPZ Challenge.

## 2. PURPOSE AND THE SCOPE OF THE PLAN.

This statistical analysis plan (SAP) provides a detailed and comprehensive description of the planned methodology and analysis to be used for the main paper(s) reporting results for this Phase IIb study; VAC074 – Protocol number KEMRI/SERU/CGMR-C/158/3844.

The results reported in these papers should follow the strategy set out in this document. Subsequent analyses of a more explanatory nature will not be bound by this strategy, though they are expected to follow the broad principles laid down here. This SAP will be consumed by the data management team, study statistician and investigators involved in the analysis of the study. This document will be updated when deemed necessary, versioned and reasons for changes documented on the version history.

## 3. HYPOTHESIS, OBJECTIVES AND ENDPOINTS

### 3.1. Hypothesis

There is no difference in the rate of *P. falciparum* infection (by PCR monitoring post CHMI) between the vaccination groups.

### 3.2. Objectives.

#### 3.2.1. Primary Objectives

| Primary Objectives | Primary Endpoints |
|---|---|
| **Safety** | |
| (a) To assess the safety and tolerability of adjuvanted R21/MM and heterologous prime boost regime of ChAd63-MVA ME-TRAP in healthy adult volunteers. | The specific endpoints for safety and reactogenicity will be actively and passively collected on adverse events on the following parameters.<br>- Occurrence of solicited local reactogenicity signs and symptoms for 7 days following vaccination.<br>- Occurrence of systemic reactogenicity signs and symptoms for 7 days following vaccination.<br>- Occurrence of unsolicited adverse events for 28 days following the vaccination. |

| | |
|---|---|
| (b) To assess the safety of intradermal sporozoite infection dose in semi-immune healthy adult volunteers. | - Occurrence of serious adverse events during the whole study duration including safety with regards to P*f*SPZ challenge administration.<br>- Change from baseline for safety laboratory measures.<br>The severity of these will be graded based on the definitions outlined in the protocol (Table 9-12) |
| **Efficacy** | |
| (c) To assess the efficacy (occurrence of P. falciparum parasitaemia, (assessed by qPCR) of adjuvanted R21 and heterologous prime- boost regime of ChAd63-MVA ME-TRAP against malaria sporozoite challenge, in healthy adult volunteers. | Diagnosis of malaria infection will be made and treatment with anti-malarial commenced immediately under the following criteria.<br>1 The density of infection >500 parasites per $\mu$L.<br>2 The volunteer develops symptoms or signs of illness, and an immediate blood film examination shows any evidence of detectable malaria parasites.<br>3 The volunteer reaches day 21 of monitoring, at which point CHMI will be completed.<br>Primary efficacy analysis will be based on the time to meeting an endpoint, as indicated by criteria 1 or 2 above. Criteria 3 will be considered as exiting the study without meeting an endpoint. |

### 3.2.2. Secondary Objective

| Secondary Objectives | Secondary Endpoints |
|---|---|
| **Immunology** | |
| 1. To assess humoral immunogenicity generated in individuals of adjuvanted R21(R21/MM) at different time points.<br><br>2. To assess cell-mediated immunogenicity by IFN-γ ELISPOT generated in individuals of heterologous prime-boost regime of ChAd63-MVA ME-TRAP at different time points. | - Comparison of Immunogenicity (antibody responses) of the R21/MM vaccination doses and the longevity of responses.<br>- Analysis of these immunological responses as potential correlates of efficacy in the efficacy analysis based on time to meeting an endpoint as per the primary analysis in Cox regression.<br><br>Antigen-specific T-cells responses to vaccination measured by ELISPOT. |
| **Efficacy** | |
| 3. To assess any differences in efficacy estimates with ID versus DVI challenge in individuals receiving R21/MM by assessing parasite density dynamics by qPCR. | - Time to first collection sample with ≥10,000parasites/μl or composite symptoms and PCR > 1000p/ml.<br>- Time to first collection sample with PCR >1,000p/μl (excluding endpoints with symptoms and lower PCR densities).<br>- Time to first collection sample with PCR >500p/μl (excluding endpoints with symptoms and lower PCR densities)<br>- Time to first collection sample with PCR >20p/μl (excluding endpoints with symptoms and lower PCR densities) |
| 4. To measure and compare the parasite growth rates and liver to blood inoculum in individuals receiving DVI versus ID sporozoite challenge in relation to naturally acquired immunity. | Mean total number of parasites 7.5 days after CHMI by vaccination groups. |

### 3.2.3. Tertiary objective.

| Tertiary Objectives | Tertiary Endpoints |
|---|---|
| **Immunology** | |
| To evaluate cell-mediated immunogenicity using flow cytometry with intracellular cytokine staining and other exploratory immunological end points. | - 1. ELISA to quantify antibodies to the vaccine components CS, NANP, TRAP and HBsAb.<br>2. Flow cytometry assays with intracellular cytokine staining to enumerate and functionally characterise immune cell populations such as effector and memory T cells (e.g., CD4+ and CD8+), T follicular helper cells, regulatory T cells, B cells, plasma cells and dendritic cells<br>3. Assays to determine antibody function in vitro<br>4. ELISPOT for enumeration of antibody-secreting cells (e.g., B and plasma cells) Antibody levels and frequencies of cells will be compared by non-parametric tests before and after vaccination. |

# 4. STUDY DESIGN OVERVIEW

## 4.1. Overview

This is a Phase IIb Sporozoite Infection study to assess the safety, immunogenicity, and protective efficacy of two malaria vaccine candidate antigens: R21/Matrix M and ChAd63-MVA encoding ME-TRAP. It is an open label, randomised controlled human malaria infection (CHMI) vaccine efficacy study. CHMI will be delivered either intradermally (ID) by inoculation of 22,500 PfSPZ Challenge or intravenously (DVI) by inoculation of 3,200 PfSPZ Challenge. The trial design is described in Table below.

We will recruit a total of 80 healthy adults who will be enrolled into 4 groups by randomisation. All eligible volunteers will be asked to attend on the day of enrolment including those in the control group where they will all be randomized to each respective group. Vaccination will be carried out over two days and the volunteers in the control group also spread over this time frame. Volunteers will further be sub-grouped into two cohorts per group who will be vaccinated and challenged sequentially over time. The sequence

adopted will ensure that COVID-19 risk is mitigated. The vaccinations and CHMI studies will take place in Kilifi at KEMRI-CGMRC and Pwani University.

CHMI, in a minimum number of 64 volunteers, will comprise the intradermal injection (ID) or the direct venous injection (DVI) of PfSPZ Challenge, which leads to a blood-stage malaria infection after 6.5 days of incubation in the liver. Twice daily blood tests will be done to monitor the density of infection and anti-malarial treatment will be given either: a) when the density of infection rises past a threshold of 500 parasites per µl (a threshold substantially lower than 2,500 parasites per µl at which clinical illness becomes more common in children in Kenya); b) if a volunteer develops symptoms or signs of illness and an immediate blood film examination shows evidence of detectable malaria parasites; or c) the volunteer reaches day 21 of monitoring, at which point CHMI will be completed or the participant is withdrawn. During the duration of CHMI, volunteers will be accommodated at a facility where they will stay for the entire duration of malaria infection. All volunteers will be provided with and required to use an ITN for the duration of the study.

| Week | 0 | 4 | 8 | 12 |
|---|---|---|---|---|
| **Group 1**<br>**N=24** | R21/ Matrix M<br>10µg /50µg | R21/ Matrix M<br>10µg /50µg | R21/ Matrix M<br>10µg /50µg | CHMI (ID)<br>N=20* |
| **Group 2**<br>**N=24** | ChAd63 ME-TRAP<br>$5x10^{10}$ vp | | MVA ME-TRAP<br>$2x10^{8}$ pfu | CHMI (ID)<br>N=20* |
| **Group 3**<br>**N=14** | R21/ Matrix M<br>10µg /50µg | R21/ Matrix M<br>10µg /50µg | R21/ Matrix M<br>10µg /50µg | CHMI (DVI)<br>N=10* |
| **Group 4**<br>**N=18** | | | | CHMI (ID)<br>N=14* |

*Indicates minimum number of volunteers required to be enrolled for CHMI.

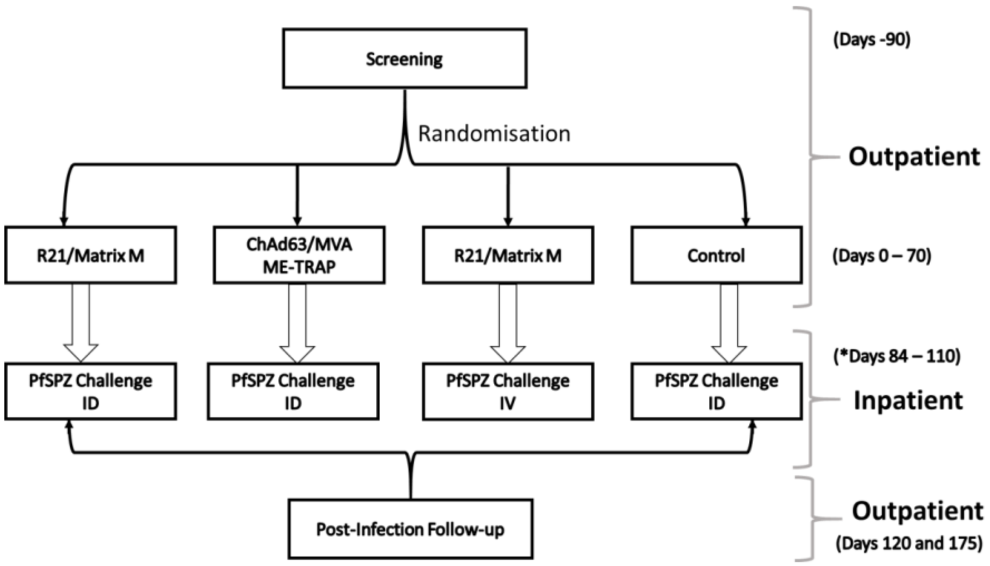


Figure 1: Schematic trial design.

### 4.2. Target Population

The target volunteer population will be healthy, adult's (aged 18-45 years) volunteers' residents from Ngerenya sub-location, Kilifi-North in Kilifi County (naïve region for malaria exposure). Inclusion and exclusion criteria are well defined in the protocol (see section 4: subsection 4.1 and 4.2).

## 5. ANALYSIS SETS.

This section defines each of the analysis sets that will be utilized. The use of each analysis set will be discussed in Section 7.

### 5.1. Intent-to-Treat (ITT) Analysis Set.

The ITT analysis set will include all subjects in the study who received at least one dose of the candidate malaria vaccine or comparator and for whom data for the observation in question are available.

### 5.2. Protocol-defined cohort (ATP)

The protocol-defined cohort for analysis of efficacy and immunogenicity will include all evaluable volunteers (i.e., those meeting all eligibility criteria, complying with the procedures defined in the protocol), for whom data concerning efficacy endpoints measures are available.

## 5.3. Groups

We will initially exclude group 3 (i.e., those receiving DVI sporozoites) on the basis that we are testing whether consistent protection is seen in this group. After conducting the primary analysis, we will compare the outcome of group 3 to the main groups (i.e., 1 and 4, and historical controls with DVI and no vaccination from the same location of residence in Kilifi) to describe the apparent impact of DVI on protection.

# 6. STATISTICAL CONSIDERATIONS.

## 6.1. General Principles.

Descriptive statistics such as geometric mean, arithmetic mean, median (interquartile range), standard deviation, and range will be used to summarize continuous variable. Categorical variables will be summarized by frequency and percentages.

Unless otherwise specified, all statistical tests will be performed using a two-sided alpha 0.05. Confidence interval will be calculated at two-sided 95% confidence level.

To facilitate data review for study progress report, only pertinent data listing will be generated. All statistical tables, tests, listing and figures will be produced using either R (version 4.2.1) or STATA (version 17) or GraphPad Prism for Windows.

## 6.2. Sample size.

This is a Phase IIb study, there will be a minimum of 64 participants. The number of participants being enrolled into the study is contingent on practical issues and is comparable to previous sporozoite challenge studies done as reported in the literature. Using a comparison of proportions assuming p=0.05 and 100% infection rates in a control arm there is 90% power to detect 40% efficacy in the n=20 groups, and 80% power to detect 50% efficacy in the control group n=14, and 80% power to detect 60% efficacy in the n=10 group.

## 6.3. Randomization and blinding.

This is a randomized, open-label clinical trial study that will enrol 80 volunteers. Randomization will be done in two blocks of 40 each and will use an unequal group allocation (12:12:7:9) as specified in the protocol. Randomization list in the form of a password protected spreadsheet will be generated using STATA. The data manager will then set up randomization in Redcap randomization module. Redcap conceals allocation until the vaccination teams clicks on randomize button then allocation will show-up to reveal the participant`s randomization arm that cannot be undone. Participants will be allocated to one of the four allocation arms.

### 6.4. Statistical interim analysis.

There is no planned interim analysis in the study protocol but real-time reporting of SAEs and SUSAR may lead to interim safety analysis if requested by the DSMB.

### 6.5. Timing of the final analysis.

Final analysis will be conducted when the last study participant last visit (LPLV) is completed (inclusive of cohort 2), and all data cleaned, and database locked.

### 6.6. Timing of the outcome assessment.

The study procedures and timings are shown in the protocol version 1.5 dated 6Apr2022; table 3, table 5, table 6 and table 8.

### 6.7. Handling of dropouts and Missing Data.

During data collection process, efforts will be made to minimize missing data occurrence. Should missing data occur, the number (percentage) of incomplete data will be reported. If possible, reasons why the data are missing should also be reported. Missing data imputation approaches will be considered for the intent-to-treat analysis.

### 6.8. Adherence and protocol deviations.

Protocol violations will be reported to the sponsor, regulatory and ethics committees as specified in the guidelines. A final Protocol Deviation Listing will be generated and reviewed by the sponsor personnel prior to freezing the database to ensure that all important deviations, including those that may lead to exclusion from analysis, are captured and summarized.

## 7. PLANNED ANALYSIS.

### 7.1. Flow of study design (consort diagram).

Flow of study design and volunteer recruitment will be presented. The flow chart will include number of screened for eligibility, number excluded and reasons for exclusion, number randomized to vaccination groups, days of follow-up for vaccination (according to schedule of procedures), number who dropped-out before being challenged and the number of volunteers who were challenged.

### 7.2. Demographic and Baseline characteristics.

Demographics and baseline characteristics, include age (years), gender, baseline height, weight, body mass index and PCR at pre-vaccination will be presented by groups to ensure comparability using the ITT analysis set.

## 7.3. Safety Analysis.

Safety analysis will be done for the intent-to-treat analysis set. This will include all solicited and unsolicited local and systemic vaccine - linked adverse events (AEs) including clinically significant laboratory abnormalities. This will be described as follows:

i. Tabulation of solicited adverse events by vaccination group, showing maximum severity and duration.

ii. A listing of unsolicited adverse events post vaccination up to 1 month post vaccination.

iii. A listing of all SAEs during the study period.

iv. Tabulation of the median and inter-quartile ranges for laboratory safety assessments (i.e., haemoglobin, full blood counts, and biochemistry results).

v. Changes in safety laboratory safety assessment by time and vaccination group.

vi. A tabulation of out-of-range laboratory abnormalities.

A separate tabulation of solicited adverse events and unsolicited adverse events during the challenge period, which will not be attributed to vaccination in the absence of a clear clinical evaluation indicating that vaccination was a cause.

## 7.4. Efficacy

Efficacy will be analysed both ITT and ATP. PCR positivity will be defined by threshold > 500 parasites per $\mu l$ as the primary endpoint but including other thresholds for comparison as above (secondary endpoints). Participants who will not meet this threshold after 21 days of follow-up or early termination will be considered right censored. Kaplan-Meier survival curves or cumulative hazard rates will be shown. Log-likelihood tests of survival functions will be conducted on PCR positivity meeting treatment thresholds. Primary comparisons will be between group 1 vs control, group 2 vs control and group 3 vs controls. Other comparisons will include DVI vs ID (i.e., group 1 vs group 3). Median time to treatment will be presented with associated 95% linear confidence interval. Both crude and adjusted cox proportional hazard rate will be presented to show magnitude of the relative risk. Vaccine efficacy (VE) will be calculated as 1- hazard rate (HR).

## 7.5. Immunogenicity

ATP cohorts will be analysed, using ELISA, immediate ex vivo and cultured ELISpots, and ICS data. Groups will be described using geometric means and medians of T cell numbers and antibody levels with 95% confidence intervals. Comparisons of immunogenicity will not be formally adjusted for multiple comparisons (e.g., using a Bonferroni adjustment) but the multiple comparisons made will be considered in the interpretation of significance levels.

We will describe the geometric mean and median spots per million PBMC by vaccine group and time point, using summed ex vivo ELISPOT responses to overlapping pools of ME-TRAP peptides, and the geometric mean and median serological responses to CS antigen. The significance test to test pair-wise comparisons over time and by vaccination group will be the students T test on log translated data. More detailed exploratory immunological analyses may be conducted later.

### 7.6. Immune response and risk on malaria infection

The risk of malaria infection as a function of immune response will be evaluated by analysing post vaccination ELISPOT and antibody results using Cox regression and logistic regression.

The ELISPOT and antibody results will be log-transformed to achieve normality of distribution, and then applied as continuous variables in models, with and without adjusting by other covariates (see above).

The hazard rate or incidence rate ratio with 95%CIs for endpoint frequency per 10-fold variation in post-vaccination responses in vaccinated participants. Kaplan-Meier graphs will be produced according to response tertiles.

## 8. VALIDATION

The safety, efficacy and immunogenicity analyses will be validated by a senior trial statistician or an appropriately qualified delegate.

## 9. STATISTICAL TABLES TO BE GENERATED.

### 9.1. Baseline characteristics.

Table 1.1 Summary of screening and enrolment.
Table 1.2 Summary of Demographics and Baseline characteristics by vaccine groups.

### 9.2. Safety Analysis.

Adverse events.

Table 2.1 Summary of adverse events in safety population according to severity post vaccination (Local and systemic 7 days post vaccination).

Table 2.2 Summary of adverse post challenge by group.

Table 2.3 Outcome of adverse events post challenge by group, safety population.

Table 2.4 Summary of adverse events in safety population according to severity post vaccination (Unsolicited general adverse events).

## 9.3. Efficacy

Table 3.1 Time to first sample with parasitaemia > 500 parasites/$\mu l$: Cox proportional hazard regression model (Adjusted and unadjusted HR; 95%CI; VE (1-HR)).

# 10. STATISTICAL LISTING TO BE GENERATED.

*Subject Data Listings*

Listing 1.1 Subjects who are screen-failures

Listing 1.2 Subjects who discontinued study after enrolment

Listing 1.3 Listing of protocol major deviations

Listing 1.4 Listing of protocol minor deviations

*Safety Listings*

Listing 2.1 Listing of Serious adverse events (SAEs)

Listing 2.2 Listing of Suspected unexpected serious adverse reactions (SUSARs)

Listing 2.3 Listing of adverse events (AEs)

Listing 2.4 Listing of death narratives.

# 11. STATISTICAL GRAPHS TO BE GENERATED

Figure 1.1 Study flowchart by cohort groups.

Figure 1.2 Kaplan-Meier estimates of the time to first episode of clinical malaria primary endpoint by groups.

Figure 1.3 Kaplan-Meier estimates of the time to first episode of clinical malaria primary endpoint by either DVI or ID

# 12. TEMPLATE TABLES

## 12.1. Solicited Adverse Events

| | Group 1<br>R21/Matrix M<br>N (%) | | | Group 2<br>ChAd63 ME-TRAP<br>N (%) | | | Group 3<br>R21/Matrix M<br>N (%) | | |
|---|---|---|---|---|---|---|---|---|---|
| | Grade | | | Grade | | | Grade | | |
| Local AE | | | | | | | | | |
| Adverse Events | 1 | 2 | 3 | 1 | 2 | 3 | 1 | 2 | 3 |
| Erythema | — | — | — | — | — | — | — | — | — |
| Induration | — | — | — | — | — | — | — | — | — |
| Pain at Injection site | — | — | — | — | — | — | — | — | — |

| Systemic AE | | | | | | | | | | | | |
|---|---|---|---|---|---|---|---|---|---|---|---|---|
| | Grade | | | | Grade | | | | Grade | | | |
| Adverse Events | 0 | 1 | 2 | 3 | 0 | 1 | 2 | 3 | 0 | 1 | 2 | 3 |
| Arthralgia | — | — | — | — | — | — | — | — | — | — | — | — |
| Chills | — | — | — | — | — | — | — | — | — | — | — | — |
| Fatigue | — | — | — | — | — | — | — | — | — | — | — | — |
| Fever | — | — | — | — | — | — | — | — | — | — | — | — |
| Headache | — | — | — | — | — | — | — | — | — | — | — | — |
| Malaise | — | — | — | — | — | — | — | — | — | — | — | — |
| Myalgia | — | — | — | — | — | — | — | — | — | — | — | — |

## 12.2. Serious Adverse Events

| SAE | Group | Relatedness |
|---|---|---|
| Event term + short description | R21/TRAP/Control | Likely/probable/possible/unlikely, and relatedness to vaccine and/or challenge |

## 12.3. Laboratory Safety Assessments

| Parameter | Median (IQR) R21 | Median (IQR) TRAP | Median (IQR) Control |
|---|---|---|---|
| Hb | | | |
| Platelets | | | |
| Creatinine | | | |

| Parameter | % Out-of-range (R21) | % Out-of-range (TRAP) | % Out-of-range (Control) |
|---|---|---|---|
| Hb | | | |
| Platelets | | | |
| Creatinine | | | |

## 12.4. Immunogenicity

| Timepoint | Mean (95%CI) R21 | Mean (95%CI) ME-TRAP | Control |
|---|---|---|---|
| Day 0 | | | |
| Day 14 | | | |
| Day 63 etc. | | | |

## 12.5. Efficacy

| | R21 | | | | TRAP | | | |
|---|---|---|---|---|---|---|---|---|
| | **Subjects (N)** | **No. of events** | **Vaccine Efficacy against control (95%CI)** | **P value** | **Subjects (N)** | **No. of events** | **Vaccine Efficacy against control (95%CI)** | **P value** |
| **Primary Endpoint** | | | | | | | | |
| ATP (Unadjusted / Adjusted) | | | | | | | | |
| | | | | | | | | |
| **Secondary Endpoint** | | | | | | | | |
| ATP (Unadjusted / Adjusted) | | | | | | | | |
| | | | | | | | | |
